# Supplementary material for: Can sterilization of disposable face masks be an alternative for imported face masks? A nationwide field study including 19 sterilization departments and 471 imported brand types during COVID-19 shortages
Source: PLoS One. 2021 Sep 14;16(9):e0257468. doi: 10.1371/journal.pone.0257468 (PMC8439445; doi:10.1371/journal.pone.0257468)

**Meetrapport/measurement report nr.**	470	**DATE**		28-7-2021	**Lighthouse 3100 output**
Client name	NL Mask BV	**Mouth Mask Branc**	CO-01 FFP2 NR	FFP2	
adress	wegalaan 11, 9742 NA, Groningen	**Mouth Mask Type**	Corodesa	Sample 2	New, imported mask
email	info@nlmask.com	**sterilsed/new**	New	New	
Phone	Samples Corodesa CO-01		CE 2163		

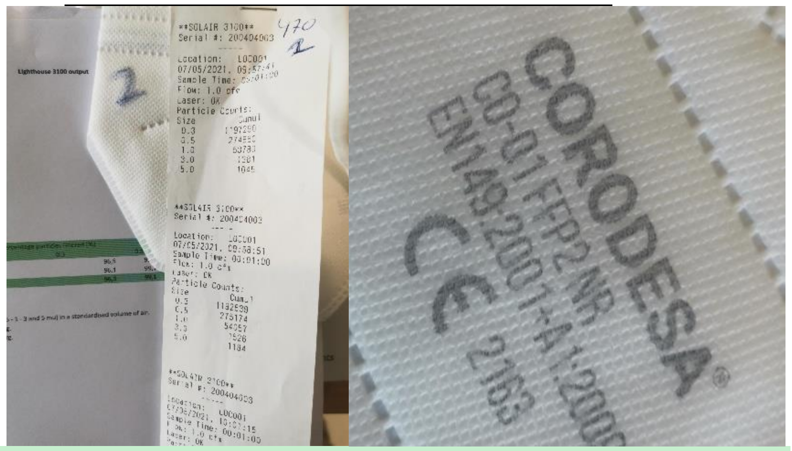

GreenCycl BV	**Operator Name**	BvS
3454 PV De Meern	**time**	16:48
info@greencycl.nl	**FOTO nr**	
Tel.: 030 – 602 38 30		

Particle measurements

benchmarking

Warning: The user of this mask may experience severe difficulty breathing due to the mask restricting the airflow. The mask has a pressure drop [dPa] of 308,0. A pressure drop [dPA] > 600 may give sam breathing difficalty.

**Flow**	**1F^3**									
**measurement time**	**1min**									
particle size	*0.3*	*0.5*	*1.0*	*3.0*	*5.0*					
environment particles 1	1543224	268616	50105	406	298					
environment particles 2	1543224	268616	50105	406	298					
avarage	**1543224**	**268616**	**50105**	**406**	**298**					
particles counted	0.3 mu	0.5 mu	1.0 mu	3 mu	5 mu	percentage particles filtered [%]				
**Sample II**						0.3	0.5	1.0	3.0	5.0
sample 4	50213	2790	226	0	0	97	99	100	100	100
sample 9	33356	1774	188	0	0	98	99	100	100	100
sample 2	59667	3287	316	0	0	96	99	99	100	100
sample 3	51792	2866	255	0	0	97	99	99	100	100
sample 1	61712	3542	314	6	3	96	99	99	99	99
sample 10	34399	1792	175	0	0	98	99	100	100	100
sample 6	43998	3736	64	1	1	97	99	100	100	100
sample 7	38336	2020	177	0	0	98	99	100	100	100
sample 5	44545	2454	231	0	0	97	99	100	100	100
sample 8	43998	3736	64	1	1	97	99	100	100	100
**mean**	**46511.5**	**2530.5**	**252.0**	**0.0**	**0.0**	**97.0**	**99.1**	**99.5**	**100.0**	**100.0**
**SD**						**0.6**	**0.3**	**0.2**	**0.5**	**0.3**
**Percentage from mean**						**0.6**	**0.3**	**0.2**	**0.5**	**0.3**

Pressure drop measurements

benchmarking

Warning: The user of this mask may experience severe difficulty breathing due to the mask restricting the airflow. The mask has a pressure drop [dPa] of 641,2. A pressure drop [dPA] > 600 may give breathing difficalty.

**Flow**	**1F^3**	
**measurement time**	**1min**	From LJLogM
sample 6	634.7	0.66
sample 1	634.7	0.68
sample 2	644.0	0.65
sample 8	644	0.67
sample 10	644	0.66
sample 5	634.7	0.66
sample 7	644.0	0.66
sample 4	644.0	0.67
sample 9	644	0.65
sample 3	644.0	0.66
**mean**	**641.2**	**0.7**
**SD**	**4.5**	**0.0**

Important note:

- Measurements are done with dry environmental particles according to our own standards. The percentages indicate the percentage of particles filtered per particle size category (0.3 0.5 1 and 5 mu) in a standardised volume of air. Measurements are done at 28 liter/min. More information can be found on www.greencycl.org..
- Each sample is 1 mask. All measurements will be published anonymously on www.greencycl.org.
- Sample 1&2 represent 2 different locations on the first masker

**Meetrapport/measurement report nr.**	467	**DATE**		28-7-2021	**Lighthouse 3100 output**
Client name	JBZ	**Mouth Mask Branc**	GB2626-2006 *	KN95	
adress	Rijnzathe 2	**Mouth Mask Type**	KN95 Chinese	Sample 1	New, imported mask
email		**sterilsed/new**	New	New	
Phone					

*

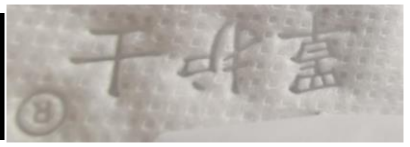

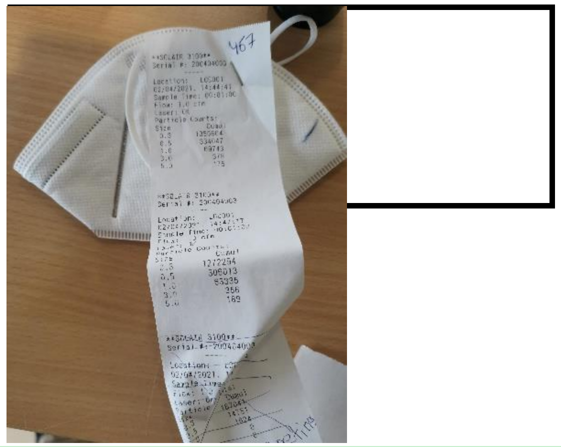

GreenCycl BV	**Operator Name**	BvS
3454 PV De Meern	**time**	16:22
info@greencycl.nl	**FOTO nr**	
Tel.: 030 – 602 38 30		

Particle measurements

benchmarking					
**Flow**	**1F^3**				
**measurement time**	**1min**				
particle size	*0.3*	*0.5*	*1.0*	*3.0*	*5.0*
environment particles 1	1404370	292847	57379	484	362
environment particles 2	1404370	292847	57379	484	362
avarage	**1404370**	**292847**	**57379**	**484**	**362**

Warning: The user of this mask may experience severe difficulty breathing due to the mask restricting the airflow. The mask has a pressure drop [dPa] of 308,0. A pressure drop [dPA] > 600 may give sam breathing difficalty.

particles counted	0.3 mu	0.5 mu	1.0 mu	3 mu	5 mu	percentage particles filtered [%] 0.3	0.5	1.0	3.0	5.0
**Sample II**										
sample 8	181594	14771	1663	0	0	87	95	97	100	100
sample 9	188908	15166	1590	0	0	87	95	97	100	100
sample 2	178964	14165	1511	5	4	87	95	97	99	99
sample 4	172930	14324	1510	0	0	88	95	97	100	100
sample 10	182867	15079	1631	0	0	87	95	97	100	100
sample 6	168435	13564	1451	0	0	88	95	97	100	100
sample 7	187777	15334	1620	0	0	87	95	97	100	100
sample 1	134797	14314	1376	0	0	90	95	98	100	100
sample 5	172477	13851	1402	0	0	88	95	98	100	100
sample 3	180156	14564	1552	0	0	87	95	97	100	100
**mean**	**183936.0**	**14665.5**	**1550.5**	**2.5**	**2.0**	**86.9**	**95.0**	**97.3**	**99.5**	**99.4**
**SD**						**1.1**	**0.2**	**0.2**	**0.3**	**0.3**
**Percentage from mean**						**1.3**	**0.2**	**0.2**	**0.3**	**0.4**

Pressure drop measurements

Warning: The user of this mask may experience severe difficulty breathing due to the mask restricting the airflow. The mask has a pressure drop [dPa] of 392,9. A pressure drop [dPA] > 600 may give breathing difficalty.

benchmarking		
**Flow**	**1F^3**	
**measurement time**	**1min**	From LJLogM
sample 7	392.0	0.66
sample 10	392	0.66
sample 2	382.7	0.65
sample 5	392.0	0.66
sample 4	401.3	0.67
sample 6	392.0	0.66
sample 1	401.3	0.68
sample 8	401	0.67
sample 9	383	0.65
sample 3	392.0	0.66
**mean**	**393.2**	**0.7**
**SD**	**6.9**	**0.0**

Important note:

- Measurements are done with dry environmental particles according to our own standards. The percentages indicate the percentage of particles filtered per particle size category (0.3 0.5 1 and 5 mu) in a standardised volume of air. Measurements are done at 28 liter/min. More information can be found on www.greencycl.org..
- Each sample is 1 mask. All measurements will be published anonymously on www.greencycl.org.
- Sample 1&2 represent 2 different locations on the first masker

**Meetrapport/measurement report nr.**	470	**DATE**		28-7-2021	**Lighthouse 3100 output**
Client name	Dromedary Industries	**Mouth Mask Branc**	MCZ-KZ	FFP2	
adress	Duindistelstraat 11, 8300 Knokke-Zoute	**Mouth Mask Type**	Mezorrison	Sample 3	New, imported mask
email	franzjr@dromedarvindunstries.com	**sterilsed/new**	New FFP2	New	
Phone	N/A		CE 0370		

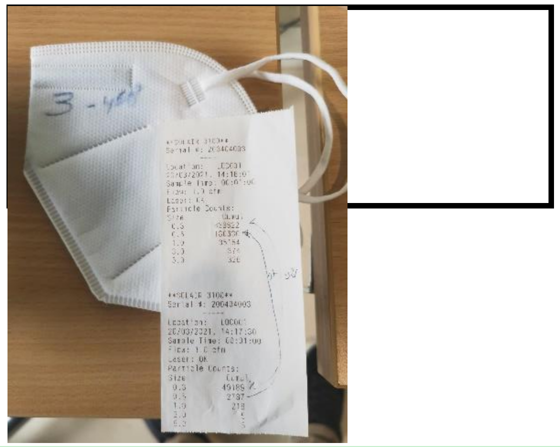

GreenCycl BV	**Operator Name**	BvS
3454 PV De Meern	**time**	17:38
info@greencycl.nl	**FOTO nr**	
Tel.: 030 – 602 38 30		

Particle measurements

benchmarking

Flow **1F^3**

measurement time **1min**

Warning: The user of this mask may experience severe difficulty breathing due to the mask restricting the airflow. The mask has a pressure drop [dPa] of 308,0. A pressure drop [dPA] > 600 may give sam breathing difficalty.

particle size	*0.3*	*0.5*	*1.0*	*3.0*	*5.0*					
environment particles 1	1464709	307809	59699	806	593					
environment particles 2	1464709	307809	59699	806	593					
avarage	**1464709**	**307809**	**59699**	**806**	**593**					
particles counted	0.3 mu	0.5 mu	1.0 mu	3 mu	5 mu	percentage particles filtered [%]				
**Sample II**						0.3	0.5	1.0	3.0	5.0
sample 5	82562	6938	870	1	0	94	98	99	100	100
sample 10	75997	6347	766	0	0	95	98	99	100	100
sample 2	73306	6052	765	2	0	95	98	99	100	100
sample 3	77823	6415	786	2	2	95	98	99	100	100
sample 8	84054	7161	906	1	0	94	98	98	100	100
sample 1	83485	7004	852	2	2	94	98	99	100	100
sample 6	79882	6892	641	0	0	95	98	99	100	100
sample 7	81314	6916	839	1	0	94	98	99	100	100
sample 9	81256	6914	843	0	0	94	98	99	100	100
sample 4	75088	6442	809	0	0	95	98	99	100	100
**mean**	**74651.5**	**6199.5**	**765.5**	**1.0**	**0.0**	**94.9**	**98.0**	**98.7**	**99.9**	**100.0**
**SD**						**0.3**	**0.1**	**0.1**	**0.1**	**0.1**
**Percentage from mean**						**0.3**	**0.1**	**0.1**	**0.1**	**0.1**

Pressure drop measurements

benchmarking

Flow **1F^3**

measurement time **1min**

Warning: The user of this mask may experience severe difficulty breathing due to the mask restricting the airflow. The mask has a pressure drop [dPa] of 372,4. A pressure drop [dPA] > 600 may give breathing difficalty.

		From LJLogM
sample 4	373.3	0.67
sample 8	364	0.67
sample 2	373.3	0.65
sample 10	373	0.66
sample 1	364.0	0.68
sample 5	373.3	0.66
sample 6	382.7	0.66
sample 7	373.3	0.66
sample 3	382.7	0.66
sample 9	364	0.65
**mean**	372.4	**0.7**
**SD**	**6.9**	**0.0**

Important note:

- Measurements are done with dry environmental particles according to our own standards. The percentages indicate the percentage of particles filtered per particle size category (0.3 0.5 1 and 5 mu) in a standardised volume of air. Measurements are done at 28 liter/min. More information can be found on www.greencycl.org..
- Each sample is 1 mask. All measurements will be published anonymously on www.greencycl.org.
- Sample 1&2 represent 2 different locations on the first masker

		**masker material type**	**label code**	**output**	**pressure [Pa]**		**pressure drop [delta Pa]**
		**atmosphere**	**ref value**	**0.24**	**-9.33**		-9.33
	**Pressure measurement on different surface part per measurement!**						
27 July 2021		470	NL Mask BV Corodesa FFP2			16:48	
Sample 9	1	Corodesa FFP2	0.93	634.7	644.0	#470	
Sample 7		Corodesa FFP2	0.93	634.7	644.0	#470	
Sample 2		Corodesa FFP2	0.93	634.7	644.0	#470	
Sample 5		Corodesa FFP2	0.92	625.3	634.7	#470	
Sample 4		Corodesa FFP2	0.93	634.7	644.0	#470	
Sample 6		Corodesa FFP2	0.92	625.3	634.7	#470	
Sample 10		Corodesa FFP2	0.93	634.7	644.0	#470	
Sample 8		Corodesa FFP2	0.93	634.7	644.0	#470	
Sample 1		Corodesa FFP2	0.92	625.3	634.7	#470	
Sample 3		Corodesa FFP2	0.93	634.7	644.0	#470	
**Mean**				**631.2**	**640.5**		
**SD**					**4.5**		**0.703895673 % of mean**
27 July 2021		467	JBZ KN95 mask			16:22	
Sample 3	2	JBZ KN95	0.66	382.7	392.0	#472	
Sample 10		JBZ KN95	0.66	382.7	392.0	#472	
Sample 2		JBZ KN95	0.65	373.3	382.7	#472	
Sample 1		JBZ KN95	0.67	392.0	401.3	#472	
Sample 4		JBZ KN95	0.67	392.0	401.3	#472	
Sample 8		JBZ KN95	0.67	392.0	401.3	#472	
Sample 6		JBZ KN95	0.66	382.7	392.0	#472	
Sample 7		JBZ KN95	0.66	382.7	392.0	#472	
Sample 9		JBZ KN95	0.65	373.3	382.7	#472	
Sample 5		JBZ KN95	0.66	382.7	392.0	#472	
**Mean**				**383.8**	**393.2**		
**SD**					**6.9**		**1.751622654 % of mean**
27 July 2021		468	Dromedary Industries FFP2			17:38	
Sample 9	3	JBZ KN95	0.63	354.7	364.0	#468	
Sample 1		JBZ KN95	0.63	354.7	364.0	#468	
Sample 10		JBZ KN95	0.64	364.0	373.3	#468	
Sample 3		JBZ KN95	0.65	373.3	382.7	#468	

Sample 7	JBZ KN95	0.64	364.0	373.3	#468
Sample 6	JBZ KN95	0.65	373.3	382.7	#468
Sample 5	JBZ KN95	0.64	364.0	373.3	#468
Sample 8	JBZ KN95	0.63	354.7	364.0	#468
Sample 2	JBZ KN95	0.64	364.0	373.3	#468
Sample 4	JBZ KN95	0.64	364.0	373.3	#468
**Mean**			**365.2**	**374.5**	
**SD**				**6.9**	**1.838931726 % of mean**

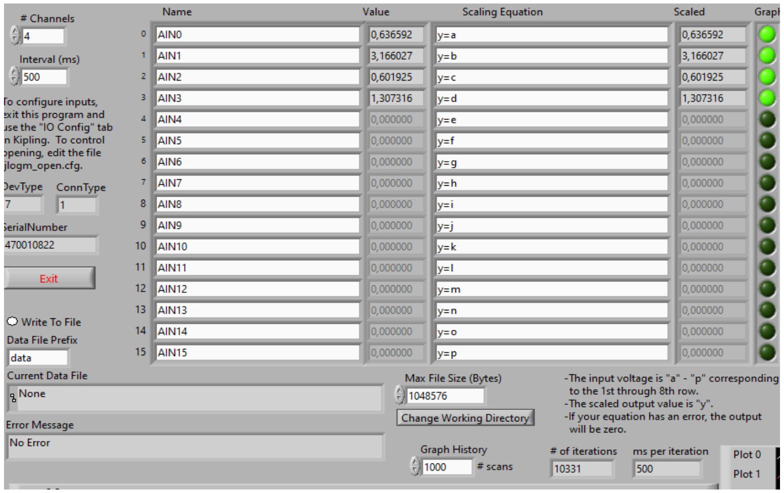

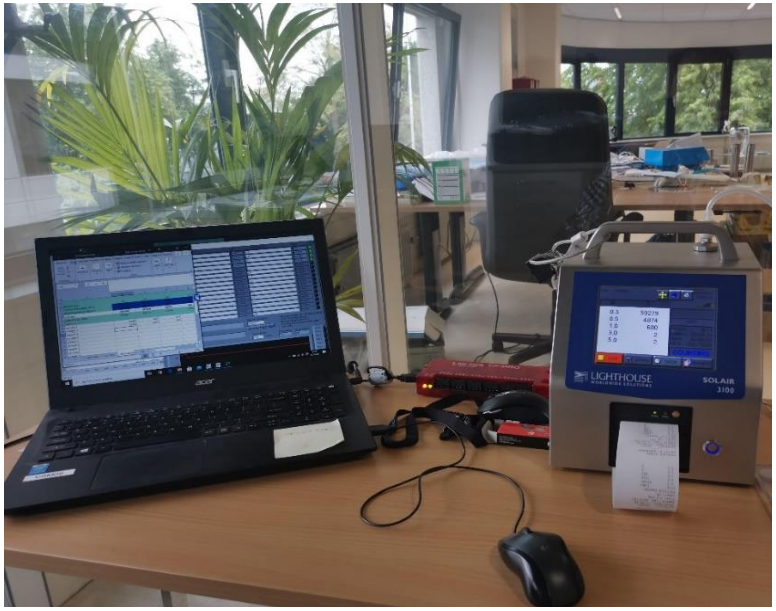

Supplement: S2 File — (PDF) [file pone.0257468.s002.pdf]
