# Supplementary material for: Can sterilization of disposable face masks be an alternative for imported face masks? A nationwide field study including 19 sterilization departments and 471 imported brand types during COVID-19 shortages
Source: PLoS One. 2021 Sep 14;16(9):e0257468. doi: 10.1371/journal.pone.0257468 (PMC8439445; doi:10.1371/journal.pone.0257468)
Supplement: S3 File — (PDF) [file pone.0257468.s003.pdf]

Validation PFE outcome of particle counter setup against continues flow system of Delft

Supplemental file 3

index nr	type of hospital	sterilisation	type of mask	standard	sample nr	data	status	0,3	0,5	1,0	5,0	avarage	Delft/Greencycl Particle test mean value [%]	delft/RIVM continues flow test available emasurements [%]	delft mean (when multiple measurements) mean value [%]	differnce [%] with RIVM data [%]
88	Uni Hospital	121 steam	3M 1862+	FFP2	sample 5	27-3-2020	new	93,3	98,1	99,5	99,8	97,7		99		
89	Uni Hospital	121 steam	3M 1872v+	FFP2	sample 1	27-3-2020	new	95,6	98,8	99,5	98,8	98,2				
90	Uni Hospital	121 steam	3M 1872v+	FFP2	sample 2	27-3-2020	new	97,8	99,5	99,9	100,0	99,3				
91	Uni Hospital	121 steam	3M 1872v+	FFP2	sample 3	27-3-2020	new	96,1	99,0	99,6	100,0	98,7				
92	Uni Hospital	121 steam	3M 1872v+	FFP2	sample 4	27-3-2020	new	95,3	98,8	99,7	100,0	98,4				
93	Uni Hospital	121 steam	3M 1872v+	FFP2	sample 5	27-3-2020	new	92,1	97,6	99,0	99,8	97,1				
122	Uni Hospital	121 steam	3M 1872v+	FFP2	sample 1	30-3-2020	new	89,8	95,4	97,6	100,0	95,7				
123	Uni Hospital	121 steam	3M 1872v+	FFP2	sample 2	30-3-2020	used	91,8	96,6	98,4	100,0	96,7				
124	Uni Hospital	121 steam	3M 1872v+	FFP2	sample 3	30-3-2020	used	92,4	96,9	98,6	100,0	97,0				
125	Uni Hospital	121 steam	3M 1872v+	FFP2	sample 1	2-4-2020	used	90,1	94,9	97,9	99,5	95,6				
126	Uni Hospital	121 steam	3M 1872v+	FFP2	sample 2	2-4-2020	used	90,7	95,2	97,7	100,0	95,9				
135	hospital	121 steam	3M 1872v+	FFP2	sample 1	1-4-2020	used	94,8	96,4	96,9	100,0	97,0				
136	hospital	121 steam	3M 1872v+	FFP2	sample 2	1-4-2020	1xused	98,1	99,3	99,7	99,4	99,1				
137	hospital	3x 121 steam	3M 1872v+	FFP2	sample 1	1-4-2020	1xused	97,5	99,1	99,7	99,4	98,9				
138	hospital	3x 121 steam	3M 1872v+	FFP2	sample 2	1-4-2020	1xused	94,4	96,4	99,6	96,7	96,8				
139	hospital	5x 121 steam	3M 1872v+	FFP2	sample 1	1-4-2020	1xused	93,6	96,7	97,7	99,4	96,9				
140	hospital	5x 121 steam	3M 1872v+	FFP2	sample 2	1-4-2020	1xused	97,1	99,0	99,6	100,0	98,9	97,5		99	**1,5**
158	independent CSA	3x 121 steam	3M 1872v+	FFP2	sample 1	6-4-2020	3x used	80,0	97,3	95,8	86,3	89,9		99		
159	independent CSA	3x 121 steam	3M 1872v+	FFP2	sample 2	6-4-2020	3x used	85,0	94,3	97,6	99,5	94,1	92,0		99	**7,0**
322	Uni Hospital	121 steam	3M 1861+	FFP1	sample 1	22-3-2020	new	NP	94,0	NP	97,6	95,8		94		
323	Uni Hospital	121 steam	3M 1861+	FFP1	sample 2	22-3-2020	new	NP	89,4	NP	96,8	93,1				
324	Uni Hospital	121 steam	3M 1861+	FFP1	sample 3	22-3-2020	new	NP	87,3	NP	98,1	92,7				
325	Uni Hospital	121 steam	3M 1861+	FFP1	sampel 4	22-3-2020	new	NP	95,7	NP	97,9	96,8	94,6		94	**0,6**
328	Uni Hospital	3x 121 steam	3M 1861+	FFP1	sample 1	22-3-2020	new	NP	94,0	NP	97,6	95,8		88		
329	Uni Hospital	3x 121 steam	3M 1861+	FFP1	sample 2	22-3-2020	new	NP	89,4	NP	96,8	93,1		95		
330	Uni Hospital	3x 121 steam	3M 1861+	FFP1	sample 3	22-3-2020	new	NP	87,3	NP	98,1	92,7				
331	Uni Hospital	3x 121 steam	3M 1861+	FFP1	sample 4	22-3-2020	new	NP	95,7	NP	97,9	96,8				
332	Uni Hospital	3x 121 steam	3M 1861+	FFP1	sample 1	22-3-2020	new	NP	98,8	NP	98,2	98,5	95,4		91,5	**3,9**
14	Uni Hospital	121 steam	3M Aura 1862+	FFP2	sample 1a	24-3-2020	new	92,2	97,2	99,3	100,0	97,2		98		
15	Uni Hospital	121 steam	3M Aura 1862+	FFP2	sample 1b	24-3-2020	new	92,3	97,3	99,4	99,9	97,2		98		
16	Uni Hospital	121 steam	3M Aura 1862+	FFP2	sample 2a	24-3-2020	new	94,0	98,0	99,6	100,0	97,9		99,5		
17	Uni Hospital	121 steam	3M Aura 1862+	FFP2	sample 2b	24-3-2020	new	94,0	97,9	99,5	100,0	97,9				
36	Uni Hospital	121 steam	3M Aura 1862+	FFP2	sample 1	25-3-2020	1xused	87,3	94,2	97,4	100,0	94,7				
37	Uni Hospital	121 steam	3M Aura 1862+	FFP2	sample 2	25-3-2020	1xused	89,0	94,9	97,7	100,0	95,4				
38	Uni Hospital	121 steam	3M Aura 1862+	FFP2	sample 1	25-3-2020	1xused	86,0	93,1	99,7	100,0	94,7				
39	Uni Hospital	121 steam	3M Aura 1862+	FFP2	sample 2	25-3-2020	1xused	87,7	93,9	97,2	100,0	94,7				
40	Uni Hospital	121 steam	3M Aura 1862+	FFP2	sample 1	25-3-2020	1xused	86,0	93,1	99,7	100,0	94,7				
41	Uni Hospital	121 steam	3M Aura 1862+	FFP2	sample 2	25-3-2020	1xused	87,7	93,9	97,2	100,0	94,7				
42	Uni Hospital	121 steam	3M Aura 1862+	FFP2	sample 1	25-3-2020	1xused	89,3	94,8	97,8	100,0	95,5				
43	Uni Hospital	121 steam	3M Aura 1862+	FFP2	sample 2	25-3-2020	1xused	89,7	95,1	97,9	100,0	95,7				
44	Uni Hospital	121 steam	3M Aura 1862+	FFP2	sample 2	25-3-2020	1xused	87,7	93,9	97,2	100,0	94,7				
45	Uni Hospital	121 steam	3M Aura 1862+	FFP2	sample 1	25-3-2020	1xused	86,0	93,1	99,7	100,0	94,7				
46	Uni Hospital	121 steam	3M Aura 1862+	FFP2	sample 2	25-3-2020	1xused	87,7	93,9	97,2	100,0	94,7				
47	Uni Hospital	121 steam	3M Aura 1862+	FFP2	sample 1	25-3-2020	1xused	89,3	94,8	97,8	100,0	95,5				
48	Uni Hospital	121 steam	3M Aura 1862+	FFP2	sample 2	25-3-2020	1xused	89,7	95,1	97,9	100,0	95,7				
49	independent CSA	121 steam	3M Aura 1862+	FFP2	sample 1	1-4-2020	1xused	97,6	99,3	99,7	99,9	99,1	95,8		98,5	**2,7**
50	independent CSA	2x 121 steam	3M Aura 1862+	FFP2	sample 1	2-4-2020	2xused	97,5	99,3	99,6	100,0	99,1	99,1	97	97	**2,1**
51	independent CSA	3x 121 steam	3M Aura 1862+	FFP2	sample 1	3-4-2020	3xused	93,6	98,0	99,1	99,8	97,6	97,6	98	98	**0,4**
80	Uni Hospital	122 steam	3M Aura 1862+	FFP2	sample 1	27-3-2020	new	99,2	99,8	99,9	100,0	99,7				
81	Uni Hospital	2x 121 steam	3M Aura 1862+	FFP2	sample 1	27-3-2020	new	95,2	98,6	99,5	100,0	98,3	98,3	99	99	**0,7**
127	Uni Hospital	H2O2 sterrad	barrier	FFP2	sample 1a	1-4-2020	used	84,6	94,7	97,8	99,6	94,2		97		
128	Uni Hospital	H2O2 sterrad	barrier	FFP2	sample 1b	1-4-2020	used	86,0	95,2	98,2	100,0	94,8		98		
129	Uni Hospital	H2O2 sterrad	barrier	FFP2	sample 2a	1-4-2020	used	80,6	93,1	97,6	100,0	92,8				
130	Uni Hospital	H2O2 sterrad	barrier	FFP2	sample 2b	1-4-2020	used	80,8	93,3	97,6	100,0	92,9				
131	Uni Hospital	H2O2 sterrad	barrier	FFP2	sample 1a	1-4-2020	used	82,3	93,6	97,4	100,0	93,3				
132	Uni Hospital	H2O2 sterrad	barrier	FFP2	sample 1b	1-4-2020	used	87,2	96,0	98,5	99,9	95,4				
133	Uni Hospital	H2O2 sterrad	barrier	FFP2	sample 2a	1-4-2020	used	81,9	93,5	97,3	99,5	93,0				
134	Uni Hospital	H2O2 sterrad	barrier	FFP2	sample 2b	1-4-2020	used	74,9	89,8	95,5	100,0	90,0	93,3		97,5	**4,2**
30	independent CSA	121 steam	Hakyard Health	FFP1	sample 1	24-3-2020	used	86,8	95,9	99,3	100,0	95,5		97		
31	independent CSA	121 steam	Hakyard Health	FFP1	sample 2	24-3-2020	used	89,4	96,9	99,5	100,0	96,5		98		
75	independent CSA	121 steam	Hakyard Health	FFP1	sample 1	26-3-2020	new	80,3	95,8	99,5	100,0	93,9	95,3		97,5	**2,2**
195	hospital	H2O2 sterrad	Health FFP2 NR	FFP2	sample 1a	3-4-2020	new	60	85	95	100	85,1		91		
196	hospital	H2O2 sterrad	Health FFP2 NR	FFP2	sample 1b	3-4-2020	new	64	88	96	100	86,8				
197	hospital	H2O2 sterrad	Health FFP2 NR	FFP2	sample 2a	3-4-2020	new	93	98	99	100	97,5				
198	hospital	H2O2 sterrad	Health FFP2 NR	FFP2	sample 2b	3-4-2020	new	81	94	98	100	93,2	90,7		91	**0,3**
78	Uni Hospital	H2O2 sterrad	i OP-Air M5101	FFP2	sample 1	27-3-2020	new	90,7	97,2	99,0	99,2	96,5		95		
79	Uni Hospital	H2O2 sterrad	i OP-Air M5101	FFP2	sample 2	27-3-2020	new	89,9	99,7	98,6	99,2	96,8	96,7		95	**1,7**
