# Supplementary material for: Can sterilization of disposable face masks be an alternative for imported face masks? A nationwide field study including 19 sterilization departments and 471 imported brand types during COVID-19 shortages
Source: PLoS One. 2021 Sep 14;16(9):e0257468. doi: 10.1371/journal.pone.0257468 (PMC8439445; doi:10.1371/journal.pone.0257468)
Supplement: S4 File — (PDF) [file pone.0257468.s004.pdf]

particle counter	Solar 3100	Supplemental file 4
interface	particle chamber	www.misit.nl
Flow	1cf/min	28 Liter/min @ 21 m/s
measurement time	1 minute	autor T.Horeman / JvDobbelsteen / D Robertson

ID	client type	sterilisation	Brand name	Grade	sample number	date	status	percentage 0.3mu particles filtered [%]	percentage 0.5mu particles filtered [%]	percentage 1mu particles filtered [%]	percentage 5mu particles filtered [%]
163	independent CSA	121 steam	KN95 CE FFP2 GB2626-2006	FFP2	43927	used	7,905595	16,41173904	30,56385421	97,09208401	37,99331815
162	independent CSA	121 steam	KN95 CE FFP2 GB2626-2006	FFP2	43927	used	16,41529	34,81234999	55,2768955	99,6365105	51,53526196
1	independent CSA	121 steam	pools masker MB20 NRD	FFP2	43914	used	83,43675	94,68837861	98,25421007	98,45389288	93,70830863
2	independent CSA	121 steam	pools masker MB20 NRD	FFP2	43914	used	83,29217	94,6200933	98,14441196	99,77912755	93,95895069
3	independent CSA	121 steam	pools masker MB20 NRD	FFP2	43914	used	83,56057	94,70649512	98,3859678	99,77912755	94,10804096
4	independent CSA	121 steam	pools masker MB20 NRD	FFP2	43914	used	67,35721	84,44627932	94,02423793	98,12258421	85,9875781
5	independent CSA	121 steam	pools masker MB20 NRD	FFP2	43914	used	69,22748	85,12146772	94,20540481	99,22694644	86,94532429
6	independent CSA	121 steam	pools masker MB20 NRD	FFP2	43914	used	67,37659	84,71872376	93,89797011	95,6929873	85,42156762
7	independent CSA	121 steam	Green Klinion	FFP2	43914	used	43,4946	69,670175	88,5864866	99,55825511	75,32737886
8	independent CSA	121 steam	Green Klinion	FFP2	43914	used	51,26509	74,81944459	90,86479735	100	79,23733186
9	independent CSA	121 steam	white	surgical mask	43914	used	69,04653	85,74788089	95,27868132	99,88956378	87,49066381
10	independent CSA	121 steam	white	surgical mask	43914	used	69,88408	85,64057541	95,20731255	99,88956378	87,65538263
11	independent CSA	121 steam	IMG europe green	FFP2	43914	used	68,85753	86,73314032	96,13510657	100	87,93144345
12	independent CSA	121 steam	IMG europe green	FFP2	43914	used	68,96021	86,75474078	96,12412676	100	87,9597687
13	independent CSA	121 steam	IMG europe green	FFP2	43914	used	62,56747	83,39064422	95,33907028	99,88956378	85,29668818
14	Uni Hospital	121 steam	3M Aura 1862+	FFP2	43914	new	92,24512	97,1508304	99,32474163	100	97,18017216
15	Uni Hospital	121 steam	3M Aura 1862+	FFP2	43914	new	92,29054	97,27903955	99,36591592	99,88956378	97,20626547
16	Uni Hospital	121 steam	3M Aura 1862+	FFP2	43914	new	94,04618	97,98000913	99,56904242	100	97,89880672
17	Uni Hospital	121 steam	3M Aura 1862+	FFP2	43914	new	93,96086	97,93541464	99,53335804	100	97,85740818
18	independent CSA	121 steam	B701	FFP2	43914	used	45,38185	71,15781919	90,29110224	100	76,70769375
19	independent CSA	121 steam	B702	FFP2	43914	used	46,60219	73,2314628	91,27379531	100	77,77686189
20	independent CSA	121 steam	Msafe	FFP2	43914	used	89,37372	95,19355052	98,34753846	99,88956378	95,70109217
21	independent CSA	121 steam	Msafe	FFP2	43914	used	88,64665	94,55529194	97,98245975	99,88956378	95,26849097
22	independent CSA	121 steam	My-T-Gear 301	FFP1	43914	used	88,13073	94,24173696	97,63659571	99,88956378	94,9746566
23	independent CSA	121 steam	My-T-Gear 301	FFP1	43914	used	85,61997	92,78684184	97,05192078	99,88956378	93,8370738
26	independent CSA	121 steam	Medicom surgical mask	IIR	43914	used	50,55501	77,49232661	93,66739408	100	80,42868156
27	independent CSA	121 steam	Medicom surgical mask	IIR	43914	used	50,55501	77,38571792	93,34623461	100	80,32173952
28	independent CSA	121 steam	Medicom surgical mask	IIR	43914	used	52,1244	78,31662782	93,5905354	99,77912755	80,95267377
29	independent CSA	121 steam	Medicom surgical mask	IIR	43914	used	51,07814	77,73155745	93,53472136	99,92637585	80,56769828
30	independent CSA	121 steam	Hakyard Health	FFP1	43914	used	86,82747	95,89591368	99,31101687	100	95,50860005
31	independent CSA	121 steam	Hakyard Health	FFP1	43914	used	89,41121	96,94318732	99,48120394	100	96,45890141
34	Uni Hospital	134 steam	3M 1861+	FFP1	43915	new	77,7	88,70225071	93,94337482	100	90,08640638
35	Uni Hospital	134 steam	3M 1861+	FFP1	43915	new	78,36055	88,72856248	93,81029967	100	90,22485186
36	Uni Hospital	121 steam	3M Aura 1862+	FFP2	43915	1xused	87,29014	94,18509806	97,37603112	100	94,71281831
37	Uni Hospital	121 steam	3M Aura 1862+	FFP2	43915	1xused	88,9877	94,89867336	97,70018852	100	95,39664156
38	Uni Hospital	121 steam	3M Aura 1862+	FFP2	43915	1xused	85,9859	93,14630925	99,69972788	100	94,70798474
39	Uni Hospital	121 steam	3M Aura 1862+	FFP2	43915	1xused	87,72289	93,93513622	97,23613161	100	94,72353937
40	Uni Hospital	121 steam	3M Aura 1862+	FFP2	43915	1xused	85,9859	93,14630925	99,69972788	100	94,70798474
41	Uni Hospital	121 steam	3M Aura 1862+	FFP2	43915	1xused	87,72289	93,93513622	97,23613161	100	94,72353937
42	Uni Hospital	121 steam	3M Aura 1862+	FFP2	43915	1xused	89,29094	94,79079509	97,83667585	100	95,47960263
43	Uni Hospital	121 steam	3M Aura 1862+	FFP2	43915	1xused	89,72099	95,06022765	97,88103423	100	95,66556223
44	Uni Hospital	121 steam	3M Aura 1862+	FFP2	43915	1xused	87,72289	93,93513622	97,23613161	100	94,72353937
45	Uni Hospital	121 steam	3M Aura 1862+	FFP2	43915	1xused	85,9859	93,14630925	99,69972788	100	94,70798474
46	Uni Hospital	121 steam	3M Aura 1862+	FFP2	43915	1xused	87,72289	93,93513622	97,23613161	100	94,72353937
47	Uni Hospital	121 steam	3M Aura 1862+	FFP2	43915	1xused	89,29094	94,79079509	97,83667585	100	95,47960263
48	Uni Hospital	121 steam	3M Aura 1862+	FFP2	43915	1xused	89,72099	95,06022765	97,88103423	100	95,66556223
49	independent CSA	121 steam	3M Aura 1862+	FFP2	43922	1xused	97,62808	99,28695094	99,72020098	99,88655701	99,13044742
50	independent CSA	2x 121 steam	3M Aura 1862+	FFP2	43923	2xused	97,463	99,26827388	99,64389729	100	99,09379232
51	independent CSA	3x 121 steam	3M Aura 1862+	FFP2	43924	3xused	93,58645	97,96065197	99,09963579	99,79541735	97,61053947
52	independent CSA	4x 121 steam	3M Aura 1862+	FFP2	43927	4xused	89,88798	96,69684281	98,85592819	99,38625205	96,20675171
53	independent CSA	121 steam	3M 1872v+	FFP2	43915	used	97,24651	94,79079509	97,83667585	100	97,4684959
54	independent CSA	121 steam	3M 1872v+	FFP2	43915	used	93,16546	97,01992854	98,93539884	100	97,28019617
55	independent CSA	121 steam	kolmi OP-Air M51010 BL	FFP2	43915	new	10,3785	63,92813728	85,4982214	99,88655701	64,92285285
56	independent CSA	121 steam	kolmi OP-Air M51010 BL	FFP2	43915	new	8,018239	61,7726768	84,79531166	99,88655701	63,61819621
57	independent CSA	121 steam	kolmi OP-Air M51010 BL	FFP2	43915	new	25,85959	61,04173574	81,87789502	99,88655701	67,16644555
58	independent CSA	121 steam	kolmi OP-Air M51010 BL	FFP2	43915	new	21,93738	56,41875714	78,80351796	100	64,28991395
59	independent CSA	121 steam	kolmi OP-Air M51010 BL	FFP2	43915	new	23,58228	59,24779902	79,75892926	100	65,64725304
60	independent CSA	121 steam	kolmi OP-Air M51010 BL	FFP2	43915	new	20,77988	61,05594409	82,30100574	100	66,03420797
61	independent CSA	121 steam	kolmi OP-Air M51010 BL	FFP2	43915	new	25,03875	63,20351104	83,24618049	100	67,87211114
62	independent CSA	121 steam	kolmi OP-Air M52010S WH	FFP2	43915	new	23,68812	60,52444627	81,48890614	100	66,42536818
63	independent CSA	121 steam	kolmi OP-Air M52010S WH	FFP2	43915	new	24,688	61,75110115	81,84377319	99,88655701	67,0423568
64	independent CSA	121 steam	SD1069	FFP2	43916	new	67,19078	88,07619777	95,83666175	99,5908347	87,67361763
65	independent CSA	121 steam	SD1069	FFP2	43916	new	67,57676	88,1421919	95,96190038	99,79541735	87,869067
66	independent CSA	121 steam	RSG 200425	FFP2	43916	new	60,90982	98,39561342	92,81181244	100	88,0293113
67	independent CSA	121 steam	RSG 200426	FFP2	43916	new	64,0481	85,87070217	93,30018187	100	85,80474624
68	independent CSA	121 steam	RSG 200427	FFP2	43916	new	77,31093	88,69837265	93,20024409	99,53198128	89,68538322
69	independent CSA	121 steam	RSG 200428	FFP2	43916	new	67,75758	84,33761612	92,04951617	99,06396256	85,80216961
70	independent CSA	134 steam	3M 1861+	FFP1	43916	new	77,65753	92,0063798	96,17952664	100	91,46085991
71	independent CSA	134 steam	3M 1861+	FFP2	43916	new	61,27301	84,23674454	92,2980071	100	84,45194014
72	independent CSA	134 steam	3M 1861+	FFP2	43916	new	75,60598	91,70941099	96,30670618	99,88144635	90,87588474
73	independent CSA	121 steam	RSG 209220	FFP2	43916	new	58,23328	88,19548979	96,08795737	99,88144635	85,59954214
74	independent CSA	121 steam	RSG 209220	FFP2	43916	new	57,52112	87,73808232	96,13882919	99,88144635	85,31986949
75	independent CSA	121 steam	Hakyard Health	FFP1	43916	new	80,25712	95,82484122	99,47845898	100	93,89010537
76	independent CSA	122 steam	surgical mask white	I	43916	new	23,15598	61,92257979	92,08630131	99,77912755	69,23599662
77	independent CSA	122 steam	surgical mask white	I	43916	new	21,13725	59,37093903	91,43025761	99,77912755	67,9293923
78	Uni Hospital	H2O2 sterrad	kolmi OP-Air M51010 BL	FFP2	43917	new	90,72331	97,24895056	98,99809096	99,18166939	96,5380051
79	Uni Hospital	H2O2 sterrad	kolmi OP-Air M51010 BL	FFP2	43917	new	89,85145	99,66873535	98,63929921	99,18166939	96,83528725
80	Uni Hospital	122 steam	3M Aura 1862+	FFP2	43917	new	99,15371	99,7955476	99,93230344	100	99,72039094
81	Uni Hospital	2x 121 steam	3M Aura 1862+	FFP2	43917	new	95,1734	98,61800527	99,47873651	100	98,31753473
82	Uni Hospital	3x 121 steam	3M Aura 1862+	FFP2	43917	new	93,66717	98,0581335	99,21471994	99,79541735	97,68386024
83	Uni Hospital	121 steam	3M Aura 1862+	FFP2	43917	new	93,6724	98,12197096	99,3433434	100	97,78442905
84	Uni Hospital	121 steam	3M Aura 1862+	FFP2	43917	new	91,27413	97,28949598	98,92362474	97,74959083	96,30921068
85	Uni Hospital	121 steam	3M Aura 1862+	FFP2	43917	new	95,28102	98,83971104	99,66151722	100	98,44556215
86	Uni Hospital	121 steam	3M Aura 1862+	FFP2	43917	new	97,59824	99,46514561	99,83075861	100	99,22353643
87	Uni Hospital	121 steam	3M Aura 1862+	FFP2	43917	new	93,66684	98,24533253	99,45165789	100	97,84095711
88	Uni Hospital	121 steam	3M 1862+	FFP2	43917	new	93,26931	98,1262843	99,47873651	99,79541735	97,66743779
89	Uni Hospital	121 steam	3M 1872v+	FFP2	43917	new	95,57076	98,78450026	99,50581513	98,77250409	98,15839388
90	Uni Hospital	121 steam	3M 1872v+	FFP2	43917	new	97,78392	99,52294439	99,8781462	100	99,29625384
91	Uni Hospital	121 steam	3M 1872v+	FFP2	43917	new	96,1386	99,02863541	99,61412963	100	98,69534162
92	Uni Hospital	121 steam	3M 1872v+	FFP2	43917	new	95,25279	98,77414824	99,66828687	100	98,42380525
93	Uni Hospital	121 steam	3M 1872v+	FFP2	43917	new	92,10763	97,58194056	99,01839992	99,79541735	97,12584693
94	hospital	121 steam	kolmi OP-Air M51010 BL	FFP2	43917	new	16,376	46,15827894	77,11179409	100	59,91151918
95	Uni Hospital	H2O2 sterrad	kolmi OP-Air M51010 BL	FFP2	43917	new	90,09194	97,16872241	99,04547855	100	96,57653436
96	Uni Hospital	121 steam	kolmi OP-Air M51010 BL	FFP2	43917	new	14,67537	66,97346195	88,23138014	100	67,47005401
96	Uni Hospital	121 steam	kolmi OP-Air M51010S BL	FFP2	43918	new	25,11907	39,78897948	60,15604568	100	56,26602404
97	Uni Hospital	121 steam	3M FFP2D 5525	FFP2	43917	new	90,09595	94,47512197	94,22020748	100	94,69781871
98	Uni Hospital	121 steam	3M FFP2D 5525	FFP2	43917	new	89,42519	92,80475172	94,75634208	100	94,24657075
99	Uni Hospital	121 steam	3M FFP2D 5525	FFP2	43917	new	90,81042	93,80221212	95,27940023	100	94,97300793
100	Uni Hospital	121 steam	SUP AIR 23200	FFP2	43917	new	86,35774	96,15175767	97,98622614	94,85179407	93,83687847
101	Uni Hospital	121 steam	SUP AIR 23200	FFP2	43917	new	89,13723	97,00761879	98,37851975	98,12792512	95,66282295
102	Uni Hospital	121 steam	SUP AIR 23200	FFP2	43917	new	88,98013	97,0615017	98,65312527	100	96,17368981
103	Uni Hospital	121 steam	MsafeFFP3 4310	FFP3	43917	new	97,1789	99,49500434	99,79077674	99,06396256	98,88216188
104	Uni Hospital	121 steam	MsafeFFP3 4310	FFP3	43917	new	98,33046	99,68547417	99,69924157	96,25585023	98,49275647
105	Uni Hospital	121 steam	MsafeFFP3 4310	FFP3	43917	new	98,34285	99,71680144	99,77770029	100	99,45933909
106	Uni Hospital	121 steam	UVEX Niosh N95	FFP2	43917	new	89,12455	96,2858384	95,21401796	99,53198128	95,0390957
107	Uni Hospital	121 steam	UVEX Niosh N95	FFP2	43917	new	84,49103	91,18450511	93,67099643	100	92,33663373
108	Uni Hospital	121 steam	UVEX Niosh N95	FFP2	43917	new	86,23408	91,99274878	94,75634208	100	93,24579197
110	Uni Hospital	121 steam	kolmi OP-Air M51010S BL	FFP2	43919	new	28,37504	42,97934906	61,24139133	98,12792512	57,6809262
111	Uni Hospital	121 steam	kolmi OP-Air M51010S BL	FFP2	43919	new	16,376	46,15827894	77,11179409	100	59,91151918
114	Uni Hospital	H2O2 sterrad	kolmi OP-Air M52010 WH	FFP2	43920	new	91,23014	96,19826303	98,87529571	100	96,57592535
115	Uni Hospital	H2O2 sterrad	kolmi OP-Air M52010 WH	FFP2	43920	new	87,83137	95,13455039	97,60354188	99,79541735	95,09121979
116	Uni Hospital	H2O2 sterrad	kolmi OP-Air M52010 WH	FFP2	43920	new	90,81531	96,36989149	98,31435573	100	96,3748881
117	Uni Hospital	H2O2 sterrad	kolmi OP-Air M52010 WH	FFP2	43920	new	91,48059	96,68217744	98,4565185	100	96,65482117
118	Uni Hospital	H2O2 sterrad	kolmi OP-Air M52010 WH	FFP2	43920	new	91,23014	96,51827045	98,59191162	100	96,58508118
119	Uni Hospital	H2O2 sterrad	kolmi OP-Air M52010 WH	FFP2	43920	new	87,83137	95,13455039	97,60354188	99,79541735	95,09121979
120	Uni Hospital	H2O2 sterrad	kolmi OP-Air M52010 WH	FFP2	43920	new	88,09261	94,7824097	97,98345081	100	95,21461828
121	Uni Hospital	H2O2 sterrad	kolmi OP-Air M52010 WH	FFP2	43920	new	88,72669	95,05701972	98,06313209	100	95,46170947
122	Uni Hospital	121 steam	3M 1872v+	FFP2	43920	new	89,78388	95,41271657	97,61884071	100	95,70385942
123	Uni Hospital	121 steam	3M 1872v+	FFP2	43920	used	91,75596	96,62794053	98,40097332	100	96,69621971
124	Uni Hospital	121 steam	3M 1872v+	FFP2	43920	used	92,35184	96,8737642	98,57478057	100	96,9500954
125	Uni Hospital	121 steam	3M 1872v+	FFP2	43923	used	90,09632	94,86231862	97,9051851	99,48717949	95,58775111
126	Uni Hospital	121 steam	3M 1872v+	FFP2	43923	used	90,74297	95,24833713	97,72051822	100	95,9279568
127	Uni Hospital	H2O2 sterrad	barrier	FFP2	43922	used	84,61004	94,65425589	97,8032417	99,61340206	94,1702361
128	Uni Hospital	H2O2 sterrad	barrier	FFP2	43922	used	85,95583	95,18275775	98,15501989	100	94,8234009
129	Uni Hospital	H2O2 sterrad	barrier	FFP2	43922	used	80,56223	93,09516387	97,5735053	100	92,8077249
130	Uni Hospital	H2O2 sterrad	barrier	FFP2	43922	used	80,79484	93,3205663	97,55945224	100	92,91871497
131	Uni Hospital	H2O2 sterrad	barrier	FFP2	43922	used	82,29083	93,57679802	97,40270238	100	93,31758345
132	Uni Hospital	H2O2 sterrad	barrier	FFP2	43922	used	87,15608	95,97119083	98,50736215	99,86666667	95,37532561
133	Uni Hospital	H2O2 sterrad	barrier	FFP2	43922	used	81,85457	93,47839472	97,34897195	99,51534733	93,04932117
134	Uni Hospital	H2O2 sterrad	barrier	FFP2	43922	used	74,86487	89,80997749	95,47516428	100	90,03750332
135	hospital	121 steam	3M 1872v+	FFP2	43922	used	94,7919	96,35648317	96,9423054	100	97,02267098
136	hospital	121 steam	3M 1872v+	FFP2	43922	1xused	98,10538	99,31779957	99,69780912	99,39698492	99,12949235

137	hospital	3x 121 steam	3M 1872v+	FFP2	43922	1xused	97,49408	99,12010308	99,66997575	99,39698492	98,92028503
138	hospital	3x 121 steam	3M 1872v+	FFP2	43922	1xused	94,41248	96,42080829	99,6182852	96,68341709	96,78374785
139	hospital	5x 121 steam	3M 1872v+	FFP2	43922	1xused	93,60358	96,7359424	97,74947315	99,39698492	96,87149444
140	hospital	5x 121 steam	3M 1872v+	FFP2	43922	1xused	97,1416	99,00207532	99,58249944	100	98,93154286
141	hospital	121 steam	2R surgical mask with elastics blaauw	2R	43923	new	54,87392	72,97546829	85,19841111	99,52531646	78,14327899
142	hospital	121 steam	2R surgical mask with elastics blaauw	2R	43923	new	54,32915	72,65548943	84,85334831	100	77,95949809
143	hospital	121 steam	2R surgical mask with elastics blaauw	2R	43923	new	49,39129	71,2097211	84,66075513	100	76,31544071
144	hospital	H2O2 sterrad	2R surgical mask with elastics blaauw	2R	43923	new	68,02766	82,90877345	91,35336837	100	85,57244921
145	hospital	H2O2 sterrad	2R surgical mask with elastics blaauw	2R	43923	new	68,44152	83,51187496	91,43762789	100	85,84775572
146	hospital	3x H2O2 sterrad	2R surgical mask with elastics blaauw	2R	43923	new	67,09528	82,4212664	91,62219636	100	85,28468644
146	hospital	3x H2O2 sterrad	2R surgical mask with elastics blaauw	2R	43923	new	67,09528	82,4212664	91,62219636	100	85,28468644
147	hospital	3x H2O2 sterrad	2R surgical mask with elastics blaauw	2R	43923	new	44,34123	65,68240749	81,0375958	99,76265823	72,70597343
147	hospital	3x H2O2 sterrad	2R surgical mask with elastics blaauw	2R	43923	new	44,34123	65,68240749	81,0375958	99,76265823	72,70597343
148	hospital	5x H2O2 sterrad	2R surgical mask with elastics blaauw	2R	43923	new	58,8366	76,88836852	88,07527184	99,76265823	80,89072442
149	hospital	5x H2O2 sterrad	2R surgical mask with elastics blaauw	2R	43923	new	49,40696	68,94864889	82,97556474	98,81329114	75,03611702
150	hospital	H2O2 sterrad	Medline surgical mask	2R	43923	new	42,79271	81,25481386	90,01843816	100	78,51648946
151	hospital	H2O2 sterrad	Medline surgical mask	2R	43923	new	42,39038	65,43224487	81,62976724	100	72,36309918
152	hospital	H2O2 sterrad	Medline surgical mask	2R	43923	new	41,88002	64,34611682	80,41534388	99,75609756	71,59939537
153	hospital	121 steam	3M 1862+	FFP2	43923	new	94,93024	97,62977375	98,51524268	84,87804878	93,98832595
154	hospital	H2O2 sterrad	L+D mask	FFP2	43923	new	12,51552	20,83660944	25,32544051	83,90243902	35,64500119
155	hospital	H2O2 sterrad	L+D mask	FFP2	43923	new	8,928992	15,26215501	17,27364729	77,56097561	29,75644253
156	independent CSA	3x 121 steam	3M Aura 1862+	FFP2	43927	3x used	80,57485	90,9908357	95,64137237	98,30371567	91,37769347
157	independent CSA	3x 121 steam	3M Aura 1862+	FFP2	43927	3x used	86,91594	93,80427667	97,33010472	99,75767367	94,45199824
158	independent CSA	3x 121 steam	3M 1872v+	FFP2	43927	3x used	80,04977	97,2988206	95,79526708	86,34146341	89,8713299
159	independent CSA	3x 121 steam	3M 1872v+	FFP2	43927	3x used	84,99568	94,30459724	97,646015	99,51219512	94,11462272
160	independent CSA	121 steam	KN95 CE FFP2 GB2626-2006	FFP2	43927	used	97,97469	99,5097098	99,7924463	100	99,31921029
161	independent CSA	121 steam	KN95 CE FFP2 GB2626-2006	FFP2	43927	used	96,43058	99,42133973	99,81131482	99,75767367	98,85522589
164	independent CSA	121 steam	ASATEX FMP3V	FFP3	43927	used	90,68231	94,48505346	96,37724457	99,75767367	95,32557118
165	independent CSA	121 steam	Msafe 6204	FFP2	43927	used	80,35053	88,25987345	92,76392339	99,6365105	90,25271008
166	independent CSA	121 steam	INXS	FFP2	43927	used	76,68761	86,61029893	91,41482437	99,75767367	88,61760247
171	hospital	2x 121 steam	Deltaplus M1200C	FFP2	43927	new	75,15795	85,62971852	90,93367716	100	87,93033622
172	hospital	2x 121 steam	Deltaplus M1200C	FFP2	43927	new	76,94028	86,71568841	91,65068084	98,9095315	88,55404613
175	hospital	121 steam	Deltaplus M1200C	FFP2	43927	new	82,35117	89,22932577	92,98562848	100	91,14153182
176	hospital	121 steam	Deltaplus M1200C	FFP2	43927	new	77,09614	87,29696705	92,31107896	99,6365105	89,08517397
179	hospital	121 steam	B702C	FFP2	43924	used	81,48423	84,52345625	91,63181232	100	89,40987514
180	hospital	121 steam	B702C	FFP2	43924	used	81,2976	87,22888937	93,66961225	99,87883683	90,51873464
181	hospital	121 steam	Wilson 9004 FFP2 CE 0194	FFP2	43923	new	51,79991	80,78425481	94,330286	99,47460595	81,59726448
182	hospital	121 steam	Wilson 9004 FFP2 CE 0194	FFP2	43923	new	44,67488	74,480836	91,10123291	99,6497373	77,47667061
183	hospital	2x 121 steam	Wilson 9004 FFP2 CE 0194	FFP2	43923	new	45,15907	76,08385505	92,03554948	99,32773109	78,15155174
184	hospital	2x 121 steam	Wilson 9004 FFP2 CE 0194	FFP2	43923	new	42,19011	73,55779721	91,3946334	100	76,78563469
185	hospital	121 steam	DFND920VNR CE0 194	FFP2	43923	new	89,94361	96,62897721	98,97668176	99,82876712	96,34450788
186	hospital	121 steam	DFND920VNR CE0 195	FFP2	43923	new	92,21868	97,37629608	99,11927529	99,82876712	97,13575405
187	hospital	H2O2 sterrad	3M Aura 1862+	FFP2	43924	new	90,41944	95,88656294	98,24056005	99,41037736	95,98923561
188	hospital	H2O2 sterrad	3M Aura 1862+	FFP2	43924	new	97,18341	99,14262684	99,71811789	99,64622642	98,92259628
189	hospital	H2O2 sterrad	3M Aura 1862+	FFP2	43924	new	70,49732	85,50485249	93,91650697	99,7826087	87,42532089
190	hospital	H2O2 sterrad	3M Aura 1862+	FFP2	43924	new	76,06671	89,00849576	95,47589524	100	90,13777628
191	hospital	2x H2O2 sterrad	3M Aura 1862+	FFP2	43924	new	39,80362	65,18291734	82,30968269	99,44649446	71,68567964
192	hospital	2x H2O2 sterrad	3M Aura 1862+	FFP2	43924	new	43,08001	68,47398484	85,07081574	99,07749077	73,9255743
193	hospital	2x H2O2 sterrad	3M Aura 1862+	FFP2	43924	new	39,98605	65,9590675	83,30147944	99,5923913	72,20974652
194	hospital	2x H2O2 sterrad	3M Aura 1862+	FFP2	43924	new	42,44686	67,94310076	84,83046648	100	73,80510652
195	hospital	H2O2 sterrad	Halyard Health FFP2 NR CE 0086	FFP2	43924	new	59,98669	85,17263223	95,12475248	100	85,0710181
196	hospital	H2O2 sterrad	Halyard Health FFP2 NR CE 0086	FFP2	43924	new	63,59672	87,55754952	96,13861386	100	86,82322177
197	hospital	H2O2 sterrad	Halyard Health FFP2 NR CE 0086	FFP2	43924	new	92,95954	97,99197618	99,39751578	99,80879541	97,53945739
198	hospital	H2O2 sterrad	Halyard Health FFP2 NR CE 0086	FFP2	43924	new	80,97003	93,72983704	98,05636941	100	93,18905807
199	hospital	2x H2O2 sterrad	Halyard Health FFP2 NR CE 0086	FFP2	43924	new	58,84165	84,11702725	94,28510274	100	84,310944
200	hospital	2x H2O2 sterrad	Halyard Health FFP2 NR CE 0086	FFP2	43924	new	60,45078	85,05408823	94,82876712	100	85,08340886
201	hospital	2x H2O2 sterrad	Halyard Health FFP2 NR CE 0086	FFP2	43924	new	60,74487	85,54583578	95,00294744	100	85,32341251
202	hospital	2x H2O2 sterrad	Halyard Health FFP2 NR CE 0086	FFP2	43924	new	65,09339	88,33175512	96,65805106	99,80879541	87,47299909
203	hospital	121 steam	Bei Bei Safety B702 FFP2 CE0194	FFP2	43924	new	68,97367	86,6169243	94,56306635	96,67170953	86,70634219
204	hospital	121 steam	Bei Bei Safety B702 FFP2 CE0194	FFP2	43924	new	69,12813	86,60277663	94,42766379	99,84871407	87,50182154
205	hospital	121 steam	Bei Bei Safety B702 FFP2 CE0194	FFP2	43924	new	69,03448	85,51020408	93,78002174	100	87,08117691
206	hospital	121 steam	Bei Bei Safety B702 FFP2 CE0194	FFP2	43924	new	68,72386	88,04314519	95,29820133	99,8489426	87,97853772
207	hospital	121 steam	3M Aura 1862+	FFP2	43928	new	88,50561	94,44343332	97,55569307	100	95,12618341
208	hospital	121 steam	3M Aura 1862+	FFP2	43928	new	89,19773	94,81350342	97,62917698	100	95,41010372
209	hospital	121 steam	3M Aura 1862+	FFP2	43928	new	95,59038	98,33348901	99,34243224	100	98,31657629
210	hospital	121 steam	3M Aura 1862+	FFP2	43928	new	93,62714	97,29830711	98,84925641	100	97,44367682
211	hospital	2x 121 steam	3M Aura 1862+	FFP2	43928	new	90,02049	95,35218701	97,87566386	100	95,81208611
212	hospital	2x 121 steam	3M Aura 1862+	FFP2	43928	new	89,90606	95,27631781	97,72727273	99,72144847	95,65777576
213	hospital	2x 121 steam	3M Aura 1862+	FFP2	43928	new	91,69526	96,28922616	98,25177494	100	96,55906558
214	hospital	2x 121 steam	3M Aura 1862+	FFP2	43928	new	88,5007	94,42412808	97,68302924	100	95,15196497
215	hospital	2x 121 steam	3M Aura 1862+	FFP2	43928	new	91,69526	96,28922616	98,25177494	100	96,55906558
216	hospital	2x 121 steam	3M Aura 1862+	FFP2	43928	new	88,5007	94,42412808	97,68302924	100	95,15196497
217	hospital	3x 121 steam	3M Aura 1862+	FFP2	43928	new	86,67133	93,5994027	97,1801679	100	94,36272398
218	hospital	3x 121 steam	3M Aura 1862+	FFP2	43928	new	90,37016	95,61685176	97,97302145	100	95,99000822
219	hospital	3x 121 steam	3M Aura 1862+	FFP2	43928	new	91,49041	96,2441135	98,41420029	100	96,53718128
220	hospital	3x 121 steam	3M Aura 1862+	FFP2	43928	new	89,23535	95,02147001	97,82378321	100	95,52014971
221	hospital	3x 121 steam	3M Aura 1862+	FFP2	43928	new	89,27824	95,28996058	97,93655682	99,51100244	95,50393992
222	hospital	3x 121 steam	3M Aura 1862+	FFP2	43928	new	94,70608	97,95493235	99,2223591	100	97,97084289
223	hospital	4x 121 steam	3M Aura 1862+	FFP2	43928	new	93,32301	97,18544983	98,84815232	99,47229551	97,20722624
224	hospital	4x 121 steam	3M Aura 1862+	FFP2	43928	new	87,0379	93,75525003	97,04225864	99,73614776	94,39288901
225	hospital	4x 121 steam	3M Aura 1862+	FFP2	43928	new	81,77288	90,63320996	95,31573405	99,41089838	91,78318137
226	hospital	4x 121 steam	3M Aura 1862+	FFP2	43928	new	81,94189	90,74219346	95,40288318	99,85272459	91,98492219
227	hospital	5x 121 steam	3M Aura 1862+	FFP2	43928	new	87,88783	94,33878102	97,73750646	100	94,99103032
228	hospital	5x 121 steam	3M Aura 1862+	FFP2	43928	new	80,11737	89,47079909	94,74791467	100	91,08402066
229	hospital	5x 121 steam	3M Aura 1862+	FFP2	43928	new	92,46266	96,62786051	98,58988119	99,42028986	96,77517238
230	hospital	5x 121 steam	3M Aura 1862+	FFP2	43928	new	89,65871	95,09433351	97,79414486	100	95,63679654
230	hospital	121 steam	Uvex silv-Air 5220+	FFP2	43928	new	97,78465	98,99878877	99,39293598	100	99,04409362
231	hospital	121 steam	Uvex silv-Air 5220+	FFP2	43928	new	95,48963	97,47363625	98,40691685	99,59128065	97,74036577
232	hospital	121 steam	Uvex silv-Air 5220+	FFP2	43928	new	95,86713	98,62193064	99,41819001	100	98,47681155
233	hospital	121 steam	Uvex silv-Air 5220+	FFP2	43928	new	98,26755	99,5015256	99,84610187	99,78540773	99,35014574
234	hospital	121 steam	Uvex silv-Air 5220+	FFP2	43928	new	97,34687	99,02763962	99,51953755	100	98,97351119
235	hospital	121 steam	Uvex silv-Air 5220+	FFP2	43928	new	97,97192	99,35902451	99,77478323	100	99,27643305
236	hospital	2x 121 steam	Uvex silv-Air 5220+	FFP2	43928	new	95,33001	98,42994077	99,46692234	99,8470948	98,26849137
237	hospital	2x 121 steam	Uvex silv-Air 5220+	FFP2	43928	new	98,01884	99,41504273	99,79674017	99,5412844	99,19297701
238	hospital	2x 121 steam	Uvex silv-Air 5220+	FFP2	43928	new	91,80164	96,99932983	98,76989053	99,59016393	96,79025683
239	hospital	2x 121 steam	Uvex silv-Air 5220+	FFP2	43928	new	96,52404	98,8004021	99,60501072	100	98,73236207
240	hospital	2x 121 steam	Uvex silv-Air 5220+	FFP2	43928	new	95,13185	97,79571093	98,71346349	99,3852459	97,75656742
241	hospital	2x 121 steam	Uvex silv-Air 5220+	FFP2	43928	new	98,50066	99,57779515	99,87209871	100	99,48763953
242	hospital	3x 121 steam	Uvex silv-Air 5220+	FFP2	43928	new	93,5668	97,65167428	98,96142978	100	97,54497495
243	hospital	3x 121 steam	Uvex silv-Air 5220+	FFP2	43928	new	98,66352	99,62435679	99,89357408	100	99,54536288
244	hospital	3x 121 steam	Uvex silv-Air 5220+	FFP2	43928	new	62,67382	84,0603923	93,70034266	100	85,10863809
245	hospital	3x 121 steam	Uvex silv-Air 5220+	FFP2	43928	new	96,41969	98,82826783	99,60085853	100	98,7122043
246	hospital	4x 121 steam	Uvex silv-Air 5220+	FFP2	43928	new	55,20793	80,67301245	92,68745501	100	82,14210039
247	hospital	4x 121 steam	Uvex silv-Air 5220+	FFP2	43928	new	97,13389	99,10583761	99,64005608	99,5157385	98,84888164
248	hospital	4x 121 steam	Uvex silv-Air 5220+	FFP2	43928	new	73,58249	88,93415	95,88049434	99,7983871	89,54888068
249	hospital	4x 121 steam	Uvex silv-Air 5220+	FFP2	43928	new	98,08427	99,39400031	99,82802064	100	99,32657245
250	hospital	5x 121 steam	Uvex silv-Air 5220+	FFP2	43928	new	62,10832	84,36319914	94,57038463	99,86595174	85,22696267
251	hospital	5x 121 steam	Uvex silv-Air 5220+	FFP2	43928	new	95,84892	98,63087362	99,58747495	100	98,51681809
252	hospital	5x 121 steam	Uvex silv-Air 5220+	FFP2	43928	new	48,13365	77,27564272	90,69538057	99,31192661	78,85415003
253	hospital	5x 121 steam	Uvex silv-Air 5220+	FFP2	43928	new	86,95872	95,36191973	98,33552523	100	95,16404048
254	hospital	5x 121 steam	Uvex silv-Air 5220+	FFP2	43928	new	55,24885	83,89794105	94,0243301	99,08256881	83,06342309
255	hospital	5x 121 steam	Uvex silv-Air 5220+	FFP2	43928	new	97,06244	99,10498349	99,99589019	100	99,04082737
256	hospital	121 steam	JSP Martcare 1020A	FFP2	43928	new	88,18718	93,57359532	96,25840398	100	94,50479493
257	hospital	121 steam	JSP Martcare 1020A	FFP2	43928	new	90,08339	94,3625401	96,49225373	100	95,23454632
258	hospital	121 steam	JSP Martcare 1020A	FFP2	43928	new	90,66902	94,99573376	97,54575513	100	95,80262823
259	hospital	121 steam	JSP Martcare 1020A	FFP2	43928	new	88,98093	93,76942384	96,57709682	100	94,83186335
260	hospital	2x 121 steam	JSP Martcare 1020A	FFP2	43928	new	85,39106	92,39343977	96,05088546	100	93,45884705
261	hospital	2x 121 steam	JSP Martcare 1020A	FFP2	43928	new	83,93611	91,44906993	95,53379399	99,74747475	92,66661132
262	hospital	2x 121 steam	JSP Martcare 1020A	FFP2	43928	new	83,76551	90,63556851	93,92995416	100	92,08275938
263	hospital	2x 121 steam	JSP Martcare 1020A	FFP2	43928	new	85,90165	92,94344023	95,94588581	100	93,69774525
264	hospital	3x 121 steam	JSP Martcare 1020A	FFP2	43928	new	82,119	90,30227243	93,88149082	96,2962963	90,64976554
265	hospital	3x 121 steam	JSP Martcare 1020A	FFP2	43928	new	76,49613	85,60792261	90,13474116	97,27095517	87,37743737
266	hospital	3x 121 steam	JSP Martcare 1020A	FFP2	43928	new	77,82819	87,28922162	91,42151553	99,64349376	89,04560584
267	hospital	3x 121 steam	JSP Martcare 1020A	FFP2	43928	new	81,07424	88,63607584	91,69013474	100	90,35011236
268	hospital	121 steam	My-T-Gear 313	FFP3	43928	new	78,65773	87,20070413	91,88781917	99,79338843	89,38491044
269	hospital	121 steam	My-T-Gear 313	FFP3	43928	new	76,47133	83,52870606	88,89912097	99,17355372	87,01817829
270	hospital	121 steam	My-T-Gear 313	FFP3	43928	new	81,20826	87,31457196	90,66632971	99,63963964	89,70719981
271	hospital	121 steam	My-T-Gear 313	FFP3	43928	new	84,89936	88,96515028	91,72773987	99,45945946	91,26292823
272	hospital	2x 121 steam	My-T-Gear 313	FFP3	43928	new	69,97204	82,66831156	89,02344302	99,56709957	85,30772263
273	hospital	2x 121 steam	My-T-Gear 313	FFP3	43928	new	83,16355	89,41348753	92,54342607	90,69264069	88,95327667
274	hospital	2x 121 steam	My-T-Gear 313	FFP3	43928	new	74,0373	83,26407271	88,88656997	98,85993485	86,26196871
275	hospital	2x 121 steam	My-T-Gear 313	FFP3	43928	new	77,89761	85,05298912	89,53007061	97,55700326	87,50941948
276	hospital	3x 121 steam	My-T-Gear 313	FFP3	43928	new	83,73021	91,31779378	95,5844927	100	92,65812375
277	hospital	3x 121 steam	My-T-Gear 313	FFP3	43928	new	83,61304	91,51984913	95,48269427	99,55357143	92,5422877
278	hospital	3x 121 steam	My-T-Gear 313	FFP3	43928	new	81,82426	91,42770803	95,61071909	99,3166287	92,04482926
279	hospital	3x 121 steam	My-T-Gear 313	FFP3	43928	new	80,00404	89,69808522	94,51444892	100	91,05414391
280	general practitioner	121 steam (23min home system)	SW1016	FFP3	43930	used	48,70589	69,66942885	77,44254232	42,56055363	59,59460259
281	general practitioner	121 steam (23min home system)	SW1016	FFP3	43930	used	58,0878	81,7850248	90,71692046	95,5017301	81,52286797
282	general practitioner	121 steam (23min home system)	SW1016	FFP3	43930	used	60,03423	83,43700995	92,73585762	100	84,05177467
283	general practitioner	121 steam (23min home system)	SW1016	FFP3	43930	used	58,37341	81,92554958	91,55997458	97,84946237	82,42709878

284	general practitioner	121 steam (23min home system)	SW1016	FFP3	43930	used	64,84574	86,33019022	92,76467318	100	85,9851516
285	general practitioner	121 steam (23min home system)	SW1016	FFP3	43930	used	64,52487	85,03297542	92,63362719	100	85,54786861
286	general practitioner	121 steam (23min home system)	3M with one-way valve	FFP2	43930	used	92,18913	95,18803882	96,29275988	99,5412844	95,80280319
287	general practitioner	121 steam (23min home system)	3M with one-way valve	FFP2	43930	used	90,30534	94,00519273	95,57455995	100	94,97127379
288	general practitioner	2x 121 steam (23min home system)	3M with one-way valve	FFP2	43930	2xused	76,44349	85,12216241	88,86714596	97,04797048	86,87019305
289	general practitioner	2x 121 steam (23min home system)	3M with one-way valve	FFP2	43930	2xused	78,54157	86,19293217	90,03701025	99,07749077	88,46224965
290	general practitioner	121 steam (23min home system)	3M Aura 1862+	FFP2	43930	used	96,20031	98,50039982	99,34424806	100	98,51123839
291	general practitioner	121 steam (23min home system)	3M Aura 1862+	FFP2	43930	used	96,60227	98,6964761	99,46584444	100	98,69114853
292	general practitioner	121 steam (23min home system)	3M Aura 1862+	FFP2	43930	used	97,80982	99,24299136	99,70263539	100	99,18886227
293	general practitioner	121 steam (23min home system)	3M Aura 1862+	FFP2	43930	used	97,30619	99,02240019	99,57253838	99,7826087	98,92093309
294	general practitioner	121 steam (23min home system)	SH2920V	FFP2	43930	used	90,69033	97,17101471	98,99074533	99,05277402	96,47621616
295	general practitioner	121 steam (23min home system)	SH2920V	FFP2	43930	used	92,93997	97,95857625	99,18117074	97,83491204	96,9786573
296	general practitioner	121 steam (23min home system)	SH2920V	FFP2	43930	used	93,53829	98,04008147	99,22468719	99,00497512	97,45200894
297	general practitioner	121 steam (23min home system)	SH2920V	FFP2	43930	used	91,44134	97,05157605	98,81016351	98,25870647	96,39044719
298	general practitioner	121 steam (23min home system)	SH2920V	FFP2	43930	used	90,97974	97,54859882	99,08282251	97,76785714	96,34475559
299	general practitioner	121 steam (23min home system)	SH2920V	FFP2	43930	used	93,56903	98,54948058	99,50223533	99,10714286	97,6819731
300	general practitioner	121 steam (23min home system)	SH2920V	FFP2	43930	used	94,24289	98,42369339	98,98226229	81,81818182	93,36675691
301	general practitioner	121 steam (23min home system)	SH2920V	FFP2	43930	used	89,94916	96,97267213	99,01134051	95	95,23329195
302	independent CSA	121 steam	Willson 9004	FFP2	43930	new	55,1321	83,76603764	95,56358856	100	83,61543072
303	independent CSA	121 steam	Willson 9004	FFP2	43930	new	53,79773	81,9538011	94,4216234	100	82,54328983
304	independent CSA	121 steam	Willson 9004	FFP2	43930	new	52,33019	82,17496375	94,83197556	100	82,33428314
305	independent CSA	121 steam	Willson 9004	FFP2	43930	new	55,94387	83,85279293	95,12050238	100	83,72929014
306	independent CSA	121 steam	Willson 9004	FFP2	43930	new	44,06984	78,04928989	93,37753974	100	78,87416747
307	independent CSA	121 steam	Willson 9004	FFP2	43930	new	44,99512	77,5808569	92,73144606	100	78,82685458
308	independent CSA	121 steam	Willson 9004	FFP2	43930	new	51,22891	85,61962727	96,65026769	100	83,37470087
309	independent CSA	121 steam	Willson 9004	FFP2	43930	new	49,5208	84,62233978	96,31746491	100	82,61515191
319	Uni Hospital	H2O2 sterrad	Kolmi roze OP-Air pro Oxygen	FFP1 IIR	43912	new	NP	85,43725006	NP	99,95959868	92,69842437
320	Uni Hospital	H2O2 sterrad	Kolmi roze OP-Air pro Oxygen	FFP1 IIR	43912	new	NP	83,50094096	NP	99,81646256	91,65870176
321	Uni Hospital	H2O2 sterrad	3M Aura 1862+	FFP2	43912	new	NP	99,55708049	NP	98,75679355	99,15693702
322	Uni Hospital	121 steam	3M 1861+	FFP1	43912	new	NP	94,01301281	NP	97,6163219	95,81466735
323	Uni Hospital	121 steam	3M 1861+	FFP1	43912	new	NP	89,38471604	NP	96,82099299	93,10285451
324	Uni Hospital	121 steam	3M 1861+	FFP1	43912	new	NP	87,33698585	NP	98,08497726	92,71098156
325	Uni Hospital	121 steam	3M 1861+	FFP1	43912	new	NP	95,73446097	NP	97,88066199	96,80756148
326	Uni Hospital	121 steam	3M Aura 1862+	FFP2	43912	new	NP	96,73796752	NP	98,57325611	97,65561182
327	Uni Hospital	121 steam	3M Aura 1862+	FFP2	43912	new	NP	94,45490227	NP	95,49582957	94,97536592
328	Uni Hospital	3x 121 steam	3M 1861+	FFP1	43912	new	NP	94,01301281	NP	97,6163219	95,81466735
329	Uni Hospital	3x 121 steam	3M 1861+	FFP1	43912	new	NP	89,38471604	NP	96,82099299	93,10285451
330	Uni Hospital	3x 121 steam	3M 1861+	FFP1	43912	new	NP	87,33698585	NP	98,08497726	92,71098156
331	Uni Hospital	3x 121 steam	3M 1861+	FFP1	43912	new	NP	95,73446097	NP	97,88066199	96,80756148
332	Uni Hospital	3x 121 steam	3M 1861+	FFP1	43912	new	NP	98,80187547	NP	98,16577991	98,48382769
333	Uni Hospital	5x 121 steam	3M Aura 1862+	FFP2	43912	new	NP	96,89956346	NP	98,22695905	97,56326126
334	Uni Hospital	5x 121 steam	3M Aura 1862+	FFP2	43912	new	NP	97,84211556	NP	97,04493175	97,44352366
335	Uni Hospital	5x 121 steam	3M Aura 1862+	FFP2	43912	new	NP	96,49942113	NP	95,96448493	96,23195303
336	Uni Hospital	121 steam	3M Aura 1862+	FFP2	43934	used	95,10398	97,9088681	99,09557534	99,13232104	97,81018521
337	Uni Hospital	121 steam	3M Aura 1862+	FFP2	43934	used	96,87049	98,76581302	99,4906114	99,82646421	98,73834393
338	Uni Hospital	121 steam	3M Aura 1862+	FFP2	43934	used	96,55739	98,61833156	99,4542265	98,61171367	98,31041523
339	Uni Hospital	121 steam	3M Aura 1862+	FFP2	43934	used	95,81635	98,25661386	99,37106101	99,82646421	98,31762162
340	Uni Hospital	121 steam	3M Aura 1862+	FFP2	43934	used	89,84354	94,99804975	97,59859658	99,65292842	95,52327885
341	Uni Hospital	121 steam	3M Aura 1862+	FFP2	43934	used	97,38233	99,03826562	99,66214021	99,82646421	98,97729891
342	Uni Hospital	121 steam	3M Aura 1862+	FFP2	43934	used	95,91246	98,35364114	99,13196024	99,47939262	98,21936341
343	Uni Hospital	121 steam	3M Aura 1862+	FFP2	43934	used	97,8207	99,21446716	99,69332727	100	99,18212242
344	Uni Hospital	121 steam	3M Aura 1862+	FFP2	43934	used	95,83606	98,40875264	99,36066532	99,82646421	98,35798518
345	Uni Hospital	121 steam	3M Aura 1862+	FFP2	43934	used	97,26341	98,91562314	99,47501787	99,13232104	98,69659227
346	Uni Hospital	121 steam	3M Aura 1862+	FFP2	43934	used	95,32976	98,05246847	99,12156455	97,04989154	97,38842082
347	Uni Hospital	121 steam	3M Aura 1862+	FFP2	43934	used	97,48463	99,03593696	99,67773374	100	99,04957471
348	Uni Hospital	H2O2 sterrad	MediCom 2092S-WH	IIR FFP2	43934	used	87,78923	94,01664539	97,30787119	99,41348974	94,63181017
349	Uni Hospital	H2O2 sterrad	MediCom 2092S-WH	IIR FFP2	43934	used	92,25061	96,59086035	98,50072501	100	96,83554992
350	Uni Hospital	H2O2 sterrad	MediCom 2092S-WH	IIR FFP2	43934	used	90,17947	95,47000255	98,25449372	100	95,9759922
351	Uni Hospital	H2O2 sterrad	MediCom 2092S-WH	IIR FFP2	43934	used	88,91349	94,7935665	97,59787694	100	95,32623349
352	Uni Hospital	H2O2 sterrad	MediCom 2092S-WH	IIR FFP2	43934	used	91,00595	95,86132244	98,07939591	100	96,2366673
353	Uni Hospital	H2O2 sterrad	MediCom 2092S-WH	IIR FFP2	43934	used	88,55193	94,51743681	97,60882055	100	95,16954623
354	Uni Hospital	H2O2 sterrad	MediCom 2092S-WH	IIR FFP2	43934	used	89,78312	95,36379883	97,9809034	100	95,78195497
355	Uni Hospital	H2O2 sterrad	Kolmi OP-Air Pro Oxygen M51010-BL	FFP2	43934	used	92,24226	96,66765382	98,73054089	99,1202346	96,69017168
356	Uni Hospital	H2O2 sterrad	Kolmi OP-Air Pro Oxygen M51010-BL	FFP2	43934	used	90,24275	95,45529742	97,99731881	100	95,92384057
357	Uni Hospital	H2O2 sterrad	Kolmi OP-Air Pro Oxygen M51010-BL	FFP2	43934	used	91,86057	96,57942303	98,6594074	100	96,77485004
358	Uni Hospital	H2O2 sterrad	Kolmi OP-Air Pro Oxygen M51010-BL	FFP2	43934	used	92,39012	96,68072504	98,71959728	100	96,94761134
359	Uni Hospital	H2O2 sterrad	Kolmi OP-Air Pro Oxygen M51010-BL	FFP2	43934	used	88,15114	94,36384989	97,30787119	100	94,95571488
360	independent CSA	121 steam	3M Aura 1862+	FFP2	43935	2xused	93,84132	97,21055874	98,85218878	99,50124688	97,35132914
361	independent CSA	121 steam	3M Aura 1862+	FFP2	43935	2xused	85,25312	92,21573769	96,38111567	99,6996997	93,38741859
362	independent CSA	121 steam	3M Aura 1862+	FFP2	43935	3xused	91,20346	95,83860128	98,21982252	99,83164983	96,27338408
363	independent CSA	121 steam	3M Aura 1862+	FFP2	43935	3xused	89,17983	94,49509856	97,59022641	99,80806142	95,26830341
364	general practitioner	121 steam (23min home system)	3M Aura 1862+	FFP2	43935	new	96,60742	99,08261851	99,70533266	99,43899018	98,70859151
365	general practitioner	121 steam (23min home system)	3M Aura 1862+	FFP2	43935	new	96,86759	99,13639328	99,68350545	99,06498364	98,68811824
366	general practitioner	121 steam (23min home system)	3M Aura 1862+	FFP2	43935	2xused	93,30998	97,02151994	98,85407146	99,81299673	97,24964123
367	general practitioner	121 steam (23min home system)	3M Aura 1862+	FFP2	43935	2xused	95,59651	98,24951091	99,35064049	99,43899018	98,15891222
368	general practitioner	121 steam (23min home system)	3M Aura 1862+	FFP2	43935	2xused	93,95413	97,38028593	99,02868914	100	97,59077506
369	general practitioner	121 steam (23min home system)	3M Aura 1862+	FFP2	43935	2xused	94,95024	97,86265362	99,09417077	99,81299673	97,93001652
370	general practitioner	121 steam (23min home system)	3M Aura 1862+	FFP2	43935	3xused	95,60527	98,18128919	99,26333165	99,62599345	98,16896984
371	general practitioner	121 steam (23min home system)	3M Aura 1862+	FFP2	43935	3xused	95,10139	97,95896664	99,20876362	100	98,06728053
372	general practitioner	121 steam (23min home system)	3M Aura 1862+	FFP2	43935	3xused	92,03769	96,34973664	98,38478643	99,81299673	96,64630134
373	general practitioner	121 steam (23min home system)	3M Aura 1862+	FFP2	43935	3xused	94,74444	97,765538	99,06688676	99,62599345	97,80071395
374	general practitioner	121 steam (23min home system)	Maco Pharma ZZM05	FFP2	43935	2xused	89,51817	96,50223225	98,9413803	100	96,24044524
375	general practitioner	121 steam (23min home system)	Maco Pharma ZZM06	FFP2	43935	2xused	85,93428	94,74933534	98,46118167	99,62599345	94,69269794
376	general practitioner	121 steam (23min home system)	Maco Pharma ZZM07	FFP2	43935	2xused	84,53576	94,20195636	98,12831671	99,81299673	94,16975683
377	general practitioner	121 steam (23min home system)	SH2920V	FFP2	43935	2xused	89,12437	96,37542012	98,77221942	99,06498364	95,83424858
378	general practitioner	121 steam (23min home system)	SH2920V	FFP2	43935	2xused	89,89068	96,48618009	98,84315785	97,75596073	95,74399347
379	general practitioner	121 steam (23min home system)	SH2920V	FFP2	43935	2xused	90,61017	97,11462252	99,06142996	97,942964	96,18229676
380	general practitioner	121 steam (23min home system)	SH2920V	FFP2	43935	2xused	89,48012	96,69405568	98,85407146	99,62599345	96,16355951
381	independent CSA	121 steam	3M1873V+	FFP2	43936	new	98,36599	99,47796955	99,8226769	100	99,41665924
382	independent CSA	121 steam	Asatex FFP3	FFP3	43936	new	98,4083	99,49169192	99,82698962	100	99,43174655
383	independent CSA	121 steam	KN95 wit	FFP2	43936	new	49,78336	72,87457538	86,97626354	98,97884756	77,15326081
384	independent CSA	121 steam	N wit met bloem	FFP2	43936	new	58,76075	81,82622389	92,85667837	98,43260188	82,96906317
385	hospital	1xH2O2 sterrad	FFP3EGE4015V	FFP2	43936	new	68,04462	87,86346164	95,17176041	99,59266802	87,66812655
386	hospital	2xH2O2 sterrad	FFP3EGE4015V	FFP2	43936	new	57,6112	84,01034929	94,11312849	100	83,93366834
387	hospital	1xH2O2 sterrad	FFP2 3M Aura 1862+	FFP2	43936	new	32,6435	61,82068162	79,25632895	99,92424242	68,41118803
388	hospital	2xH2O2 sterrad	FFP2 3M Aura 1862+	FFP2	43936	new	28,14438	52,85028919	70,94084212	100	62,98387748
389	hospital	3x 121 steam	KN 95 Blauw	FFP2	43936	new	80,22662	88,87091492	93,70825744	96,76190476	89,89192437
390	hospital	3x 121 steam	Witte Chinese 3D GB2626-2006	FFP2	43936	new	98,72519	99,54695672	99,81026108	100	99,52060117
391	hospital	1x 121 steam	Blauwe Chinese KN95 0004-2020031	FFP2	43936	new	80,50945	89,83406608	94,34198543	99,23809524	90,98090039
392	hospital	1x 121 steam	KN 95 Wit Chinese 3D GB2626-2006	FFP2	43936	new	54,98072	73,85237983	86,96986833	100	78,95074236
393	hospital	2x 121 steam	KN 95 Blauw Chinese 0004-20200317	FFP2	43936	new	77,16246	86,74238271	92,52766782	100	89,10812758
394	hospital	2x 121 steam	KN 95 Wit Chinese 3D GB2626-2006	FFP2	43936	new	72,38574	85,37949401	92,5922934	100	87,58938067
395	hospital	3x 121 steam	KN 95 Wit Chinese 3D GB2626-2006	FFP2	43936	new	73,53621	86,49591394	93,83633573	100	88,46711542
396	hospital	121 steam	Medicom Wit 6042002180827	FFP2	43936	new	34,89853	57,24890138	75,20558573	100	66,83825397
397	hospital	121 steam	Medicom Wit NR D Type BR	FFP2	43936	new	35,85928	57,91500059	75,58960434	100	67,34097116
398	hospital	121 steam	Kolmi Wit	FFP2	43936	new	33,73478	53,37107566	71,99379364	99,50980392	64,65236283
399	hospital	121 steam	B702 FFP2	FFP2	43936	used	95,38261	97,53125413	98,32376956	95,99977043	96,80935067
400	hospital	121 steam	SUP AIR 23100 FFP1D	FFP2	43941	used	77,10666	89,16841857	94,76995548	100	90,26125764
401	hospital	121 steam	Jiandi	FFP2	43941	used	51,25359	68,97318303	81,93880314	99,28315412	75,36218154
402	hospital	121 steam	HUIXIN	FFP2	43941	used	26,61834	42,86490418	59,02641713	99,89669421	57,10158981
403	hospital	121 steam	KN95	FFP2	43941	used	47,77596	74,31495768	87,80780023	97,59206799	76,87269766
404	hospital	121 steam	DFND FFP3 230v	FFP2	43941	used	46,12916	73,16358169	87,31996032	97,62362397	76,05908225
405	hospital	121 steam	3M 8833 FFP3 R D	FFP2	43941	used	99,1748	99,64412653	99,81965291	99,72042635	99,58975133
406	hospital	121 steam	Wit GH	FFP2	43941	used	78,83188	84,72946944	88,86438719	96,01607548	87,11045222
407	hospital	121 steam	3M Aura 9332+ Gen 3	FFP3	43941	used	99,00411	99,66458926	99,83932714	99,93010659	99,60953383
408	hospital	121 steam	Drager	FFP2	43941	used	80,96993	90,95369641	95,79299434	99,4408527	91,7893682
409	hospital	121 steam	9591 GB2626-2006KN95	FFP2	43941	used	70,13175	89,38607378	95,5995311	98,46234492	88,39492488
410	hospital	121 steam	GH2626-2006 KN95	KN95 FFP2	43941	used	57,04422	75,27835978	87,65114315	99,86021317	79,95848293
411	Uni Hospital	1xH2O2 sterrad	JSP Martcare 1020A FFP2	FFP2	43943	new	95,44774	97,29926526	97,94686827	99,70674386	97,60015546
412	Uni Hospital	1xH2O2 sterrad	Marco Pharma FFP2	FFP2	43943	new	96,74592	99,21638018	99,94438265	99,77989729	98,92164523
413	Uni Hospital	2xH2O2 sterrad	Marco Pharma FFP2	FFP2	43943	new	97,1528	99,29024989	99,875332	100	99,07959613
414	Uni Hospital	3xH2O2 sterrad	Marco Pharma FFP2	FFP2	43943	new	96,82124	99,22493455	99,83310733	99,9381953	98,95436979
414	Uni Hospital	3xH2O2 sterrad	Marco Pharma FFP2	FFP2	43943	new	96,82124	99,22493455	99,83310733	99,9381953	98,95436979
415	Uni Hospital	1x 121 steam	3M Vflex 1802	FFP2	43943	new	75,57576	86,17886179	92,78251854	99,18918919	88,43158256
416	Uni Hospital	2x 121 steam	3M Vflex 1802	FFP2	43943	new	80,2015	89,43137904	93,9977221	93,17094389	89,20038575
417	Uni Hospital	3x 121 steam	3M Vflex 1802	FFP2	43943	new	73,66858	85,75558393	93,30450016	99,79623026	88,13122428
418	Uni Hospital	1xH2O2 sterrad	Sandel FFP2	FFP2	43943	new	93,19844	97,667403	99,13189119	99,71949509	97,42930755
419	Uni Hospital	1x 121 steam	Maco Pharma FFP2	FFP2	43943	new	94,36536	98,52073279	99,63759343	99,45366275	97,99433668
420	Uni Hospital	2x 121 steam	Maco Pharma FFP2	FFP2	43943	new	90,98921	97,55214491	99,50021968	99,88031119	96,98047218
421	Uni Hospital	3x 121 steam	Maco Pharma FFP2	FFP2	43943	new	89,73837	97,23351471	99,29570277	99,92141454	96,54725098
422	Uni Hospital	1x 121 steam	Sandel FFP2 NR - 95002 EN149:2001 ·	FFP2	43952	new	91,73895	96,81831022	98,65379731	100	96,80276517
423	Uni Hospital	1x 121 steam	Sandel FFP2 NR - 95002 EN149:2001 ·	FFP2	43952	new	89,20705	95,73924418	97,98768839	99,90163934	95,70890638
424	Uni Hospital	1x 121 steam	Meixin MX-2005 EN 149:2001+A1:20	FFP2	43952	new	98,94047	99,69247624	99,6461166	96,04247104	98,58038346
425	Uni Hospital	1x 121 steam	JSP Martcare 1020A FFP2 EN149:200	FFP2	43952	new	92,38653	96,54283514	98,18279256	98,34710744	96,36481504
426	Uni Hospital	1x 121 steam	JSP Martcare 1020A FFP2 EN149:200	FFP2	43952	new	87,45862	94,10524522	97,20234223	100	94,6915524
427	Uni Hospital	1x 121 steam	Meixin MX-2005 EN 149:2001+A1:20	FFP2	43952	new	98,96799	99,34889912	99,37927356	97,28033473	98,74412481
428	Uni Hospital	1x 121 steam	3M aura 1862+ (GIRBES)	FFP2	43952	new	98,28901	99,35076029	99,77131656	100	99,35277114
429	Uni Hospital	1x 121 steam	3M aura 1862+ (GIRBES) MASK 2	FFP2	43952	new	98,25805	99,32117436	99,70567694	100	99,32122639
430	Uni Hospital	1x 121 steam	3M aura 1862+ (GIRBES) MASK 3	FFP2	43952	new	97,44124	98,8586313	99,73743436	99,80582524	98,96078276
431	Uni Hospital	1x 121 steam	3M aura 1862+ (GIRBES) MASK 4	FFP2	43952	new	82,86882	99,39349515	99,65866777	99,34924078	95,3175555

432	Uni Hospital	1x 121 steam	3M aura 1862+ (GIRBES) MASK 5	FFP2	43952	new	98,3414	99,38597554	99,65725477	99,87228608	99,31422806
433	Uni Hospital	1x 121 steam	3M aura 1862+ (GIRBES) MASK 6	FFP2	43952	new	94,68985	97,44977006	98,70803949	99,73718791	97,64621144
434	Uni Hospital	1x 121 steam	3M aura 1862+ (GIRBES) MASK 7	FFP2	43952	new	97,63538	99,08716973	99,47104677	99,84459984	99,00954964
435	Uni Hospital	1x 121 steam	3M aura 1862+ (GIRBES) MASK 8	FFP2	43952	new	98,45226	99,4078319	99,74008737	100	99,40004547
436	Uni Hospital	1x 121 steam	3M aura 1862+ (GIRBES) MASK 9	FFP2	43952	new	98,2864	99,35689943	99,70767666	99,92412747	99,31877488
437	Uni Hospital	1x 121 steam	3M aura 1862+ (GIRBES) MASK 10	FFP2	43952	new	97,42655	98,83197835	99,39011109	99,85228951	98,87523149
438	Uni Hospital	1x 121 steam	3M aura 1862+ (GIRBES) MASK 11	FFP2	43952	new	95,8168	98,02539667	98,87382211	100	98,17900346
439	Uni Hospital	1x 121 steam	3M aura 1862+ (GIRBES) MASK 12	FFP2	43952	new	98,92318	99,62829326	99,83010052	99,89118607	99,56819063
440	Uni Hospital	1x 121 steam	KN95 Staat &Co3 (Sterilized)	FFP2	43958	new	53,31506	71,16055181	79,52139054	99,06976744	75,76669257
441	Uni Hospital	1x 121 steam	KN95 Staat &Co4 (Sterilized)	FFP2	43958	new	15,51218	33,40396018	47,49188258	98,03001876	48,60950971
442	Uni Hospital	1x 121 steam	3M Aura 9320+	FFP2	43958	new	91,31098	94,38839217	96,00184733	95,98662207	94,42196127
443	Uni Hospital	1x 121 steam	3M Aura 9322+ met ademventiel	FFP2	43958	new	93,91226	96,87979423	97,92618697	99,62121212	97,08486401
444	Uni Hospital	1x 121 steam	3M 8822 met ademventiel1	FFP2	43958	new	93,22059	95,18586633	95,59354548	87,39495798	92,84873979
445	Uni Hospital	1x 121 steam	Isolatiemasker Kangya Medical	FFP2	43958	new	59,76721	77,19312281	85,64166667	99,51737452	80,52984259
446	hospital	1x 121 steam	GB262-2006KN95	KN95 FFP2	43958	new	64,73795	88,29735533	95,07373091	100	87,02725789
447	hospital	1x 121 steam	KN95 GB2626-2006	KN95 FFP2	43958	new	61,14693	82,21305245	90,73384961	98,51851852	83,15308685
448	hospital	1x 121 steam	GB2626-2006KN95	KN95 FFP2	43958	new	58,94106	77,63009169	87,15321717	99,66216216	80,84663306
449	hospital	1x 121 steam	1003 2626-2006KN95	KN95 FFP2	43958	new	65,19435	78,6973125	86,18925193	99,31623932	82,34928918
450	hospital	1x 121 steam	243 S5	KN95 FFP2	43958	new	21,71642	41,9605744	57,12844488	98,00724638	54,70317219
451	independent CSA	1x 121 steam	NRW Test Laurens (244 S1)	KN95 FFP2	43958	new	84,72166	92,4573651	94,9720914	91,66666667	90,95444678
452	independent CSA	1x 121 steam	2EX Test Laurens (244 S2)	KN95 FFP2	43958	new	68,21063	82,13627882	88,12926507	99,46808511	84,48606386
453	independent CSA	1x 121 steam	Nieuw Test Laurens (244 S3)	KN95 FFP2	43958	new	87,74855	93,26662782	94,74979822	98,75	93,62874371
454	independent CSA	1x 121 steam	Nieuw Test Laurens (244 S4)	KN95 FFP2	43958	new	41,64207	69,21451206	81,07105924	99,33510638	72,8156879
455	independent CSA	1x 121 steam	Nieuw Test Zellingen (244 S5)	KN95 FFP2	43958	new	95,63387	98,52528005	99,1225617	98,67374005	97,98886233
456	independent CSA	1x 121 steam	2e X test Pelie (244 S6)	KN95 FFP2	43958	new	45,53338	63,91617773	74,01995197	99,59785523	70,76684009
457	independent CSA	1x 121 steam	2e x X (244 S7)	KN95 FFP2	43958	new	36,65227	63,00251706	75,7865054	99,2	68,66032192
458	independent CSA	1x 121 steam	Personeel (244 S8)	KN95 FFP2	43958	new	82,34477	92,96903348	96,21089159	99,79757085	92,83056735
459	independent CSA	1x 121 steam	X (244 S9)	KN95 FFP2	43958	new	89,34398	95,15293636	96,72416076	83,92857143	91,28741292
460	independent CSA	1x 121 steam	Test nieuw argos (244 S10)	KN95 FFP2	43958	new	76,97131	85,56481455	89,09775673	99,71590909	87,8374467
461	independent CSA	1x 121 steam	Nieuw Test Laurens (244 S11)	KN95 FFP2	43958	new	33,04128	64,76237792	78,46246006	98,84615385	68,77806854
462	independent CSA	1x 121 steam	Test 2e keer sterilisatie naamloos (24	KN95 FFP2	43958	new	23,34	46,2739407	61,2988626	96,41509434	56,83197505
463	independent CSA	1x 121 steam	Test nieuw Vivia 2 (244 S13)	KN95 FFP2	43958	new	55,01159	82,14162682	90,23619229	99,26315789	81,66314253
464	independent CSA	1x 121 steam	Nieuw Test Bussum 3 (244 S14)	KN95 FFP2	43958	new	70,76278	86,10557769	91,39596974	99,25093633	86,87881494
465	independent CSA	1x 121 steam	Afheur +2 Vivvum groep (244 S15)	KN95 FFP2	43958	new	35,57006	58,94529694	71,92237631	100	66,6094326
466	independent CSA	1x 121 steam	2e Test Aafje (244 S16)	KN95 FFP2	43958	new	87,13463	93,38288338	95,58490455	99,45799458	93,89010332
467	independent CSA	1x 121 steam	Nieuw Test Argos (244 S17)	KN95 FFP2	43958	new	31,65141	59,17628082	73,38503861	99,375	65,89693178
468	independent CSA	1x 121 steam	2e x Test Aafje (244 S18)	KN95 FFP2	43958	new	84,27536	91,52825706	93,82878972	99,4140625	92,26161616
469	independent CSA	1x 121 steam	2e x test Aafje (244 S19)	KN95 FFP2	43958	new	82,89718	89,51945552	91,6152768	98,4496124	90,62038056
470	independent CSA	1x 121 steam	GB262-2006KN95	KN95 FFP2	43964	new	74,66827	79,12414135	83,06226082	97,85894207	83,67840255
471	independent CSA	1x 121 steam	KN95 GB2626-2006	KN95 FFP2	43964	new	58,45211	78,55219258	88,49248736	99,89384289	81,3476572
472	independent CSA	1x 121 steam	GB2626-2006KN95	KN95 FFP2	43964	new	28,09011	44,52131344	60,57180366	97,82608696	57,75232827
473	independent CSA	1x 121 steam	1003 2626-2006KN95	KN95 FFP2	43964	new	27,2581	47,79913205	64,82461712	100	59,97046124
474	independent CSA	1x 121 steam	243 S5	KN95 FFP2	43964	new	80,6685	92,49861836	96,77585614	99,64664311	92,39740377
475	independent CSA	1x 121 steam	243 S5	KN95 FFP2	43964	new	50,14545	71,79966096	82,97851751	99,77011494	76,17343673
476	independent CSA	1x 121 steam	243 S5	KN95 FFP2	43964	new	87,44977	92,4578482	94,90032901	99,77011494	93,64451449
477	independent CSA	1x 121 steam	243 S5	KN95 FFP2	43964	new	70,54419	78,96482808	85,88235294	99,50331126	83,72367071

H202	73
steam	371

1xSteam

361	independent CSA	121 steam	3M Aura 1862+	FFP2	14-4-2020	2xused	85,3	92,2	96,4	99,7	93,4
38	Uni Hospital	121 steam	3M Aura 1862+	FFP2	25-3-2020	1xused	86,0	93,1	99,7	100,0	94,7
40	Uni Hospital	121 steam	3M Aura 1862+	FFP2	25-3-2020	1xused	86,0	93,1	99,7	100,0	94,7
45	Uni Hospital	121 steam	3M Aura 1862+	FFP2	25-3-2020	1xused	86,0	93,1	99,7	100,0	94,7
36	Uni Hospital	121 steam	3M Aura 1862+	FFP2	25-3-2020	1xused	87,3	94,2	97,4	100,0	94,7
39	Uni Hospital	121 steam	3M Aura 1862+	FFP2	25-3-2020	1xused	87,7	93,9	97,2	100,0	94,7
41	Uni Hospital	121 steam	3M Aura 1862+	FFP2	25-3-2020	1xused	87,7	93,9	97,2	100,0	94,7
44	Uni Hospital	121 steam	3M Aura 1862+	FFP2	25-3-2020	1xused	87,7	93,9	97,2	100,0	94,7
46	Uni Hospital	121 steam	3M Aura 1862+	FFP2	25-3-2020	1xused	87,7	93,9	97,2	100,0	94,7
327	Uni Hospital	121 steam	3M Aura 1862+	FFP2	22-3-2020	new	NP	94,5	NP	95,5	95,0
207	hospital	121 steam	3M Aura 1862+	FFP2	7-4-2020	new	89	94	98	100	95,1
363	independent CSA	121 steam	3M Aura 1862+	FFP2	14-4-2020	3xused	89,2	94,5	97,6	99,8	95,3
37	Uni Hospital	121 steam	3M Aura 1862+	FFP2	25-3-2020	1xused	89,0	94,9	97,7	100,0	95,4
208	hospital	121 steam	3M Aura 1862+	FFP2	7-4-2020	new	89	95	98	100	95,4
42	Uni Hospital	121 steam	3M Aura 1862+	FFP2	25-3-2020	1xused	89,3	94,8	97,8	100,0	95,5
47	Uni Hospital	121 steam	3M Aura 1862+	FFP2	25-3-2020	1xused	89,3	94,8	97,8	100,0	95,5
340	Uni Hospital	121 steam	3M Aura 1862+	FFP2	13-4-2020	used	89,8	95,0	97,6	99,7	95,5
43	Uni Hospital	121 steam	3M Aura 1862+	FFP2	25-3-2020	1xused	89,7	95,1	97,9	100,0	95,7
48	Uni Hospital	121 steam	3M Aura 1862+	FFP2	25-3-2020	1xused	89,7	95,1	97,9	100,0	95,7
362	independent CSA	121 steam	3M Aura 1862+	FFP2	14-4-2020	3xused	91,2	95,8	98,2	99,8	96,3
84	Uni Hospital	121 steam	3M Aura 1862+	FFP2	27-3-2020	new	91,3	97,3	98,9	97,7	96,3
14	Uni Hospital	121 steam	3M Aura 1862+	FFP2	24-3-2020	new	92,2	97,2	99,3	100,0	97,2
15	Uni Hospital	121 steam	3M Aura 1862+	FFP2	24-3-2020	new	92,3	97,3	99,4	99,9	97,2
360	independent CSA	121 steam	3M Aura 1862+	FFP2	14-4-2020	2xused	93,8	97,2	98,9	99,5	97,4
346	Uni Hospital	121 steam	3M Aura 1862+	FFP2	13-4-2020	used	95,3	98,1	99,1	97,0	97,4
210	hospital	121 steam	3M Aura 1862+	FFP2	7-4-2020	new	94	97	99	100	97,4
326	Uni Hospital	121 steam	3M Aura 1862+	FFP2	22-3-2020	new	NP	96,7	NP	98,6	97,7
83	Uni Hospital	121 steam	3M Aura 1862+	FFP2	27-3-2020	new	93,7	98,1	99,3	100,0	97,8
336	Uni Hospital	121 steam	3M Aura 1862+	FFP2	13-4-2020	used	95,1	97,9	99,1	99,1	97,8
87	Uni Hospital	121 steam	3M Aura 1862+	FFP2	27-3-2020	new	93,7	98,2	99,5	100,0	97,8
17	Uni Hospital	121 steam	3M Aura 1862+	FFP2	24-3-2020	new	94,0	97,9	99,5	100,0	97,9
16	Uni Hospital	121 steam	3M Aura 1862+	FFP2	24-3-2020	new	94,0	98,0	99,6	100,0	97,9
342	Uni Hospital	121 steam	3M Aura 1862+	FFP2	13-4-2020	used	95,9	98,4	99,1	99,5	98,2
338	Uni Hospital	121 steam	3M Aura 1862+	FFP2	13-4-2020	used	96,6	98,6	99,5	98,6	98,3
209	hospital	121 steam	3M Aura 1862+	FFP2	7-4-2020	new	96	98	99	100	98,3
339	Uni Hospital	121 steam	3M Aura 1862+	FFP2	13-4-2020	used	95,8	98,3	99,4	99,8	98,3
344	Uni Hospital	121 steam	3M Aura 1862+	FFP2	13-4-2020	used	95,8	98,4	99,4	99,8	98,4
85	Uni Hospital	121 steam	3M Aura 1862+	FFP2	27-3-2020	new	95,3	98,8	99,7	100,0	98,4
345	Uni Hospital	121 steam	3M Aura 1862+	FFP2	13-4-2020	used	97,3	98,9	99,5	99,1	98,7
337	Uni Hospital	121 steam	3M Aura 1862+	FFP2	13-4-2020	used	96,9	98,8	99,5	99,8	98,7
341	Uni Hospital	121 steam	3M Aura 1862+	FFP2	13-4-2020	used	97,4	99,0	99,7	99,8	99,0
347	Uni Hospital	121 steam	3M Aura 1862+	FFP2	13-4-2020	used	97,5	99,0	99,7	100,0	99,0
49	independent CSA	121 steam	3M Aura 1862+	FFP2	1-4-2020	1xused	97,6	99,3	99,7	99,9	99,1
343	Uni Hospital	121 steam	3M Aura 1862+	FFP2	13-4-2020	used	97,8	99,2	99,7	100,0	99,2
86	Uni Hospital	121 steam	3M Aura 1862+	FFP2	27-3-2020	new	97,6	99,5	99,8	100,0	99,2
290	general practitioner	121 steam (23min home system)	3M Aura 1862+	FFP2	9-4-2020	used	96	99	99	100	98,5
291	general practitioner	121 steam (23min home system)	3M Aura 1862+	FFP2	9-4-2020	used	97	99	99	100	98,7
293	general practitioner	121 steam (23min home system)	3M Aura 1862+	FFP2	9-4-2020	used	97	99	100	100	98,9
292	general practitioner	121 steam (23min home system)	3M Aura 1862+	FFP2	9-4-2020	used	98	99	100	100	99,2
372	general practitioner	121 steam (23min home system)	3M Aura 1862+	FFP2	14-4-2020	3xused	92,0	96,3	98,4	99,8	96,6
366	general practitioner	121 steam (23min home system)	3M Aura 1862+	FFP2	14-4-2020	2xused	93,3	97,0	98,9	99,8	97,2
368	general practitioner	121 steam (23min home system)	3M Aura 1862+	FFP2	14-4-2020	2xused	94,0	97,4	99,0	100,0	97,6
373	general practitioner	121 steam (23min home system)	3M Aura 1862+	FFP2	14-4-2020	3xused	94,7	97,8	99,1	99,6	97,8
369	general practitioner	121 steam (23min home system)	3M Aura 1862+	FFP2	14-4-2020	2xused	95,0	97,9	99,1	99,8	97,9
371	general practitioner	121 steam (23min home system)	3M Aura 1862+	FFP2	14-4-2020	3xused	95,1	98,0	99,2	100,0	98,1
367	general practitioner	121 steam (23min home system)	3M Aura 1862+	FFP2	14-4-2020	2xused	95,6	98,2	99,4	99,4	98,2
370	general practitioner	121 steam (23min home system)	3M Aura 1862+	FFP2	14-4-2020	3xused	95,6	98,2	99,3	99,6	98,2
365	general practitioner	121 steam (23min home system)	3M Aura 1862+	FFP2	14-4-2020	new	96,9	99,1	99,7	99,1	98,7
364	general practitioner	121 steam (23min home system)	3M Aura 1862+	FFP2	14-4-2020	new	96,6	99,1	99,7	99,4	98,7
80	Uni Hospital	122 steam	3M Aura 1862+	FFP2	27-3-2020	new	99,2	99,8	99,9	100,0	99,7
428	Uni Hospital	1x 121 steam	3M aura 1862+ (GIRBES)	FFP2	1-5-2020	new	98,28901	99,35076029	99,77131656	100	99,4
437	Uni Hospital	1x 121 steam	3M aura 1862+ (GIRBES) MASK 10	FFP2	1-5-2020	new	97,42655	98,83197835	99,39011109	99,85228951	98,9
438	Uni Hospital	1x 121 steam	3M aura 1862+ (GIRBES) MASK 11	FFP2	1-5-2020	new	95,8168	98,02539667	98,87382211	100	98,2
439	Uni Hospital	1x 121 steam	3M aura 1862+ (GIRBES) MASK 12	FFP2	1-5-2020	new	98,92318	99,62829326	99,83010052	99,89118607	99,6
429	Uni Hospital	1x 121 steam	3M aura 1862+ (GIRBES) MASK 2	FFP2	1-5-2020	new	98,25805	99,32117436	99,70567694	100	99,3
430	Uni Hospital	1x 121 steam	3M aura 1862+ (GIRBES) MASK 3	FFP2	1-5-2020	new	97,44124	98,8586313	99,73743436	99,80582524	99,0
431	Uni Hospital	1x 121 steam	3M aura 1862+ (GIRBES) MASK 4	FFP2	1-5-2020	new	82,86882	99,39349515	99,65866777	99,34924078	95,3
432	Uni Hospital	1x 121 steam	3M aura 1862+ (GIRBES) MASK 5	FFP2	1-5-2020	new	98,3414	99,38597554	99,65725477	99,87228608	99,3
433	Uni Hospital	1x 121 steam	3M aura 1862+ (GIRBES) MASK 6	FFP2	1-5-2020	new	94,68985	97,44977006	98,70803949	99,73718791	97,6
434	Uni Hospital	1x 121 steam	3M aura 1862+ (GIRBES) MASK 7	FFP2	1-5-2020	new	97,63538	99,08716973	99,47104677	99,84459984	99,0
435	Uni Hospital	1x 121 steam	3M aura 1862+ (GIRBES) MASK 8	FFP2	1-5-2020	new	98,45226	99,4078319	99,74008737	100	99,4
436	Uni Hospital	1x 121 steam	3M aura 1862+ (GIRBES) MASK 9	FFP2	1-5-2020	new	98,2864	99,35689943	99,70767666	99,92412747	99,3
						MEAN	93,6	97,3	99,0	99,7	97,4
		2xSteam				SD	4,108017	2,05586331	0,872381898	0,711822753	1,664708284
214	hospital	2x 121 steam	3M Aura 1862+	FFP2	7-4-2020	new	89	94	98	100	95,2
216	hospital	2x 121 steam	3M Aura 1862+	FFP2	7-4-2020	new	89	94	98	100	95,2
212	hospital	2x 121 steam	3M Aura 1862+	FFP2	7-4-2020	new	90	95	98	100	95,7
211	hospital	2x 121 steam	3M Aura 1862+	FFP2	7-4-2020	new	90	95	98	100	95,8
213	hospital	2x 121 steam	3M Aura 1862+	FFP2	7-4-2020	new	92	96	98	100	96,6
215	hospital	2x 121 steam	3M Aura 1862+	FFP2	7-4-2020	new	92	96	98	100	96,6
81	Uni Hospital	2x 121 steam	3M Aura 1862+	FFP2	27-3-2020	new	95,2	98,6	99,5	100,0	98,3
50	independent CSA	2x 121 steam	3M Aura 1862+	FFP2	2-4-2020	2xused	97,5	99,3	99,6	100,0	99,1
						MEAN	91,6	96,2	98,3	100,0	96,5
		2xH2O2				SD	3,201219	1,818233735	0,798637473	0,098482839	1,456200418
191	hospital	2x H2O2 sterrad	3M Aura 1862+	FFP2	3-4-2020	new	40	65	82	99	71,7
193	hospital	2x H2O2 sterrad	3M Aura 1862+	FFP2	3-4-2020	new	40	66	83	100	72,2
194	hospital	2x H2O2 sterrad	3M Aura 1862+	FFP2	3-4-2020	new	42	68	85	100	73,8
192	hospital	2x H2O2 sterrad	3M Aura 1862+	FFP2	3-4-2020	new	43	68	85	99	73,9
						MEAN	41,3	66,9	83,9	99,5	72,9
		1xH2O2				SD	1,677888	1,570434705	1,306649263	0,381456492	1,128697462

189	hospital	H2O2 sterrad	3M Aura 1862+	FFP2	3-4-2020	new	70	86	94	100	87,4
190	hospital	H2O2 sterrad	3M Aura 1862+	FFP2	3-4-2020	new	76	89	95	100	90,1
187	hospital	H2O2 sterrad	3M Aura 1862+	FFP2	3-4-2020	new	90	96	98	99	96,0
188	hospital	H2O2 sterrad	3M Aura 1862+	FFP2	3-4-2020	new	97	99	100	100	98,9
321	Uni Hospital	H2O2 sterrad	3M Aura 1862+	FFP2	22-3-2020	new	98,0	99,6	99,8	98,8	99,0
						MEAN	86,4	93,8	97,4	99,5	94,3
		1xSteam				SD	12,51663	6,28107042	2,630235234	0,476915728	5,271755983
94	hospital	121 steam	kolmi OP-Air M51010 BL	FFP2	27-3-2020	new	16,4	46,2	77,1	100,0	59,9
56	independent CSA	121 steam	kolmi OP-Air M51010 BL	FFP2	25-3-2020	new	8,0	61,8	84,8	99,9	63,6
58	independent CSA	121 steam	kolmi OP-Air M51010 BL	FFP2	25-3-2020	new	21,9	56,4	78,8	100,0	64,3
55	independent CSA	121 steam	kolmi OP-Air M51010 BL	FFP2	25-3-2020	new	10,4	63,9	85,5	99,9	64,9
59	independent CSA	121 steam	kolmi OP-Air M51010 BL	FFP2	25-3-2020	new	23,6	59,2	79,8	100,0	65,6
60	independent CSA	121 steam	kolmi OP-Air M51010 BL	FFP2	25-3-2020	new	20,8	61,1	82,3	100,0	66,0
57	independent CSA	121 steam	kolmi OP-Air M51010 BL	FFP2	25-3-2020	new	25,9	61,0	81,9	99,9	67,2
96	Uni Hospital	121 steam	kolmi OP-Air M51010 BL	FFP2	27-3-2020	new	14,7	67,0	88,2	100,0	67,5
61	independent CSA	121 steam	kolmi OP-Air M51010 BL	FFP2	25-3-2020	new	25,0	63,2	83,2	100,0	67,9
96	Uni Hospital	121 steam	kolmi OP-Air M51010S BL	FFP2	28-3-2020	new	25,1	39,8	60,2	100,0	56,3
110	Uni Hospital	121 steam	kolmi OP-Air M51010S BL	FFP2	29-3-2020	new	28,4	43,0	61,2	98,1	57,7
111	Uni Hospital	121 steam	kolmi OP-Air M51010S BL	FFP2	29-3-2020	new	16,4	46,2	77,1	100,0	59,9
62	independent CSA	121 steam	kolmi OP-Air M52010S WH	FFP2	25-3-2020	new	23,7	60,5	81,5	100,0	66,4
63	independent CSA	121 steam	kolmi OP-Air M52010S WH	FFP2	25-3-2020	new	24,7	61,8	81,8	99,9	67,0
398	hospital	121 steam	Kolmi Wit	FFP2	15-4-2020	new	33,7	53,4	72,0	99,5	64,7
						MEAN	21,2	56,3	78,4	99,8	63,9
		1xH202				SD	6,892601	8,516484613	8,166783744	0,483126767	3,719208149
78	Uni Hospital	H2O2 sterrad	kolmi OP-Air M51010 BL	FFP2	27-3-2020	new	90,7	97,2	99,0	99,2	96,5
95	Uni Hospital	H2O2 sterrad	kolmi OP-Air M51010 BL	FFP2	27-3-2020	new	90,1	97,2	99,0	100,0	96,6
79	Uni Hospital	H2O2 sterrad	kolmi OP-Air M51010 BL	FFP2	27-3-2020	new	89,9	99,7	98,6	99,2	96,8
115	Uni Hospital	H2O2 sterrad	kolmi OP-Air M52010 WH	FFP2	30-3-2020	new	87,8	95,1	97,6	99,8	95,1
119	Uni Hospital	H2O2 sterrad	kolmi OP-Air M52010 WH	FFP2	30-3-2020	new	87,8	95,1	97,6	99,8	95,1
120	Uni Hospital	H2O2 sterrad	kolmi OP-Air M52010 WH	FFP2	30-3-2020	new	88,1	94,8	98,0	100,0	95,2
121	Uni Hospital	H2O2 sterrad	kolmi OP-Air M52010 WH	FFP2	30-3-2020	new	88,7	95,1	98,1	100,0	95,5
116	Uni Hospital	H2O2 sterrad	kolmi OP-Air M52010 WH	FFP2	30-3-2020	new	90,8	96,4	98,3	100,0	96,4
114	Uni Hospital	H2O2 sterrad	kolmi OP-Air M52010 WH	FFP2	30-3-2020	new	91,2	96,2	98,9	100,0	96,6
118	Uni Hospital	H2O2 sterrad	kolmi OP-Air M52010 WH	FFP2	30-3-2020	new	91,2	96,5	98,6	100,0	96,6
117	Uni Hospital	H2O2 sterrad	kolmi OP-Air M52010 WH	FFP2	30-3-2020	new	91,5	96,7	98,5	100,0	96,7
						MEAN	89,8	96,4	98,4	99,8	96,1
						SD	1,438868	1,404887906	0,515359209	0,322884905	0,709256543
