# Supplementary material for: Can sterilization of disposable face masks be an alternative for imported face masks? A nationwide field study including 19 sterilization departments and 471 imported brand types during COVID-19 shortages
Source: PLoS One. 2021 Sep 14;16(9):e0257468. doi: 10.1371/journal.pone.0257468 (PMC8439445; doi:10.1371/journal.pone.0257468)
Supplement: S5 File — (PDF) [file pone.0257468.s005.pdf]

Comparison between CSSD's Supplemental file 5
121 steam sterilisation at different sterilisation departments of the 3M Aura 1862+ffp2 mask

			brand and type	protection level	date arrived	status	percentage 0.3mu particles filtered [%]	percentage 0.5mu particles filtered [%]	percentage 1mu particles filtered [%]	percentage 5mu particles filtered [%]		NR	ID1	ID2	ID3	ID4	ID5
14	Uni Hospital	121 steam	3M Aura 1862+	FFP2	24-3-2020	new	92,2	97,2	99,3	100,0	97,2						
15	Uni Hospital	121 steam	3M Aura 1862+	FFP2	24-3-2020	new	92,3	97,3	99,4	99,9	97,2						
16	Uni Hospital	121 steam	3M Aura 1862+	FFP2	24-3-2020	new	94,0	98,0	99,6	100,0	97,9						
17	Uni Hospital	121 steam	3M Aura 1862+	FFP2	24-3-2020	new	94,0	97,9	99,5	100,0	97,9						
						mean	**93,1**	**97,6**	**99,4**	**100,0**	**97,5**	**4,0**	1,1	1,2	1,3	1,4	1,5
						SD	**1,0**	**0,4**	**0,1**	**0,1**	**0,4**						
36	Uni Hospital	121 steam	3M Aura 1862+	FFP2	25-3-2020	1xused	87,3	94,2	97,4	100,0	94,7						
37	Uni Hospital	121 steam	3M Aura 1862+	FFP2	25-3-2020	1xused	89,0	94,9	97,7	100,0	95,4						
38	Uni Hospital	121 steam	3M Aura 1862+	FFP2	25-3-2020	1xused	86,0	93,1	99,7	100,0	94,7						
39	Uni Hospital	121 steam	3M Aura 1862+	FFP2	25-3-2020	1xused	87,7	93,9	97,2	100,0	94,7						
40	Uni Hospital	121 steam	3M Aura 1862+	FFP2	25-3-2020	1xused	86,0	93,1	99,7	100,0	94,7						
41	Uni Hospital	121 steam	3M Aura 1862+	FFP2	25-3-2020	1xused	87,7	93,9	97,2	100,0	94,7						
42	Uni Hospital	121 steam	3M Aura 1862+	FFP2	25-3-2020	1xused	89,3	94,8	97,8	100,0	95,5						
43	Uni Hospital	121 steam	3M Aura 1862+	FFP2	25-3-2020	1xused	89,7	95,1	97,9	100,0	95,7						
44	Uni Hospital	121 steam	3M Aura 1862+	FFP2	25-3-2020	1xused	87,7	93,9	97,2	100,0	94,7						
45	Uni Hospital	121 steam	3M Aura 1862+	FFP2	25-3-2020	1xused	86,0	93,1	99,7	100,0	94,7						
46	Uni Hospital	121 steam	3M Aura 1862+	FFP2	25-3-2020	1xused	87,7	93,9	97,2	100,0	94,7						
47	Uni Hospital	121 steam	3M Aura 1862+	FFP2	25-3-2020	1xused	89,3	94,8	97,8	100,0	95,5						
48	Uni Hospital	121 steam	3M Aura 1862+	FFP2	25-3-2020	1xused	89,7	95,1	97,9	100,0	95,7						
							**87,9**	**94,2**	**98,0**	**100,0**	**95,0**	**13**	2,1	2,2	2,3	2,4	2,5
							**1,4**	**0,7**	**1,0**	**0,0**	**0,4**						
80	Uni Hospital	122 steam	3M Aura 1862+	FFP2	27-3-2020	new	99,2	99,8	99,9	100,0	99,7						
83	Uni Hospital	121 steam	3M Aura 1862+	FFP2	27-3-2020	new	93,7	98,1	99,3	100,0	97,8						
84	Uni Hospital	121 steam	3M Aura 1862+	FFP2	27-3-2020	new	91,3	97,3	98,9	97,7	96,3						
85	Uni Hospital	121 steam	3M Aura 1862+	FFP2	27-3-2020	new	95,3	98,8	99,7	100,0	98,4						
86	Uni Hospital	121 steam	3M Aura 1862+	FFP2	27-3-2020	new	97,6	99,5	99,8	100,0	99,2						
87	Uni Hospital	121 steam	3M Aura 1862+	FFP2	27-3-2020	new	93,7	98,2	99,5	100,0	97,8						
88	Uni Hospital	121 steam	3M 1862+	FFP2	27-3-2020	new	93,3	98,1	99,5	99,8	97,7						
							**94,8**	**98,6**	**99,5**	**99,6**	**98,1**	**7**	3,1	3,2	3,3	3,4	3,5
							**2,7**	**0,9**	**0,3**	**0,8**	**1,1**						
207	hospital	121 steam	3M Aura 1862+	FFP2	7-4-2020	new	89	94	98	100	95,1						
208	hospital	121 steam	3M Aura 1862+	FFP2	7-4-2020	new	89	95	98	100	95,4						
209	hospital	121 steam	3M Aura 1862+	FFP2	7-4-2020	new	96	98	99	100	98,3						
210	hospital	121 steam	3M Aura 1862+	FFP2	7-4-2020	new	94	97	99	100	97,4						
							**91,7**	**96,2**	**98,3**	**100,0**	**96,6**	**4**	6,1	6,2	6,3	6,4	6,5
							**3,4**	**1,9**	**0,9**	**0,0**	**1,6**						
290	general practitioner	steam (23min home sys	3M Aura 1862+	FFP2	9-4-2020	used	96	99	99	100	98,5						
291	general practitioner	steam (23min home sys	3M Aura 1862+	FFP2	9-4-2020	used	97	99	99	100	98,7						
292	general practitioner	steam (23min home sys	3M Aura 1862+	FFP2	9-4-2020	used	98	99	100	100	99,2						
293	general practitioner	steam (23min home sys	3M Aura 1862+	FFP2	9-4-2020	used	97	99	100	100	98,9						
364	general practitioner	steam (23min home sys	3M Aura 1862+	FFP2	14-4-2020	new	96,6	99,1	99,7	99,4	98,7						
365	general practitioner	steam (23min home sys	3M Aura 1862+	FFP2	14-4-2020	new	96,9	99,1	99,7	99,1	98,7						
							**96,9**	**98,9**	**99,6**	**99,7**	**98,8**	**6**	7,1	7,2	7,3	7,4	7,5
							**0,6**	**0,3**	**0,1**	**0,4**	**0,2**						
336	Uni Hospital	121 steam	3M Aura 1862+	FFP2	13-4-2020	used	95,1	97,9	99,1	99,1	97,8						
337	Uni Hospital	121 steam	3M Aura 1862+	FFP2	13-4-2020	used	96,9	98,8	99,5	99,8	98,7						
338	Uni Hospital	121 steam	3M Aura 1862+	FFP2	13-4-2020	used	96,6	98,6	99,5	98,6	98,3						
339	Uni Hospital	121 steam	3M Aura 1862+	FFP2	13-4-2020	used	95,8	98,3	99,4	99,8	98,3						
340	Uni Hospital	121 steam	3M Aura 1862+	FFP2	13-4-2020	used	89,8	95,0	97,6	99,7	95,5						
341	Uni Hospital	121 steam	3M Aura 1862+	FFP2	13-4-2020	used	97,4	99,0	99,7	99,8	99,0						
342	Uni Hospital	121 steam	3M Aura 1862+	FFP2	13-4-2020	used	95,9	98,4	99,1	99,5	98,2						
343	Uni Hospital	121 steam	3M Aura 1862+	FFP2	13-4-2020	used	97,8	99,2	99,7	100,0	99,2						
344	Uni Hospital	121 steam	3M Aura 1862+	FFP2	13-4-2020	used	95,8	98,4	99,4	99,8	98,4						
345	Uni Hospital	121 steam	3M Aura 1862+	FFP2	13-4-2020	used	97,3	98,9	99,5	99,1	98,7						
346	Uni Hospital	121 steam	3M Aura 1862+	FFP2	13-4-2020	used	95,3	98,1	99,1	97,0	97,4						
347	Uni Hospital	121 steam	3M Aura 1862+	FFP2	13-4-2020	used	97,5	99,0	99,7	100,0	99,0						
							**95,9**	**98,3**	**99,3**	**99,4**	**98,2**	12	4,1	4,2	4,3	4,4	4,5
							**2,1**	**1,1**	**0,6**	**0,8**	**1,0**						
428	Uni Hospital	1x 121 steam	3M aura 1862+ (GIRBES)	FFP2	1-5-2020	new	98,28901	99,35076	99,77132	100	99,4						
429	Uni Hospital	1x 121 steam	3M aura 1862+ (GIRBES) MASK 2	FFP2	1-5-2020	new	98,25805	99,32117	99,70568	100	99,3						
430	Uni Hospital	1x 121 steam	3M aura 1862+ (GIRBES) MASK 3	FFP2	1-5-2020	new	97,44124	98,85863	99,73743	99,80583	99,0						
431	Uni Hospital	1x 121 steam	3M aura 1862+ (GIRBES) MASK 4	FFP2	1-5-2020	new	82,86882	99,3935	99,65867	99,34924	95,3						
432	Uni Hospital	1x 121 steam	3M aura 1862+ (GIRBES) MASK 5	FFP2	1-5-2020	new	98,3414	99,38598	99,65725	99,87229	99,3						
433	Uni Hospital	1x 121 steam	3M aura 1862+ (GIRBES) MASK 6	FFP2	1-5-2020	new	94,68985	97,44977	98,70804	99,73719	97,6						
434	Uni Hospital	1x 121 steam	3M aura 1862+ (GIRBES) MASK 7	FFP2	1-5-2020	new	97,63538	99,08717	99,47105	99,8446	99,0						
435	Uni Hospital	1x 121 steam	3M aura 1862+ (GIRBES) MASK 8	FFP2	1-5-2020	new	98,45226	99,40783	99,74009	100	99,4						
436	Uni Hospital	1x 121 steam	3M aura 1862+ (GIRBES) MASK 9	FFP2	1-5-2020	new	98,2864	99,3569	99,70768	99,92413	99,3						
437	Uni Hospital	1x 121 steam	3M aura 1862+ (GIRBES) MASK 10	FFP2	1-5-2020	new	97,42655	98,83198	99,39011	99,85229	98,9						
438	Uni Hospital	1x 121 steam	3M aura 1862+ (GIRBES) MASK 11	FFP2	1-5-2020	new	95,8168	98,0254	98,87382	100	98,2						
439	Uni Hospital	1x 121 steam	3M aura 1862+ (GIRBES) MASK 12	FFP2	1-5-2020	new	98,92318	99,62829	99,8301	99,89119	99,6						
							**96,4**	**99,0**	**99,5**	**99,9**	**98,7**	12	5,1	5,2	5,3	5,4	5,5
							**4,4**	**0,6**	**0,4**	**0,2**	**1,2**						

For figure taken from left table

means					NR	ID1	ID2	ID3	ID4	ID5	SD				
**93,1**	**97,6**	**99,4**	**100,0**	**97,5**	**4,0**	**1,1**	**1,2**	**1,3**	**1,4**	**1,5**	**0,9**	**0,4**	**0,1**	**0,0**	**0,3**
**87,9**	**94,2**	**98,0**	**100,0**	**95,0**	**13**	**2,1**	**2,2**	**2,3**	**2,4**	**2,5**	**1,3**	**0,7**	**0,9**	**0,1**	**0,4**
**94,8**	**98,6**	**99,5**	**99,6**	**98,1**	**7**	**3,1**	**3,2**	**3,3**	**3,4**	**3,5**	**2,5**	**0,8**	**0,3**	**0,8**	**1,0**
**91,7**	**96,2**	**98,3**	**100,0**	**96,6**	**4**	**6,1**	**6,2**	**6,3**	**6,4**	**6,5**	**3,0**	**1,6**	**0,8**	**0,1**	**1,3**
**96,9**	**98,9**	**99,6**	**99,7**	**98,8**	**6**	**7,1**	**7,2**	**7,3**	**7,4**	**7,5**	**0,5**	**0,3**	**0,1**	**0,4**	**0,2**
**95,9**	**98,3**	**99,3**	**99,4**	**98,2**	**12**	**4,1**	**4,2**	**4,3**	**4,4**	**4,5**	**2,0**	**1,1**	**0,5**	**0,8**	**1,0**
**96,4**	**99,0**	**99,5**	**99,9**	**98,7**	**12**	**5,1**	**5,2**	**5,3**	**5,4**	**5,5**	**4,2**	**0,6**	**0,3**	**0,2**	**1,1**

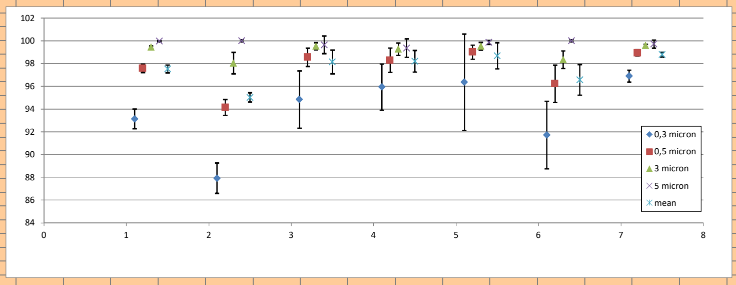

t test mean values		h1	h2	h3	h4	h5	h6	h7
	h1		7,95E-05	0,231016	0,307528	0,00344	0,073238	0,011927
	h2			0,000121	0,140468	3,4E-14	4,52E-08	1,29E-07
	h3				0,067247	0,185237	0,88967	0,335515
	h4					0,064217	0,121429	0,063928
	h5						0,08144	0,793409
	h6							0,303238
	h7							
