# Supplementary material for: Can sterilization of disposable face masks be an alternative for imported face masks? A nationwide field study including 19 sterilization departments and 471 imported brand types during COVID-19 shortages
Source: PLoS One. 2021 Sep 14;16(9):e0257468. doi: 10.1371/journal.pone.0257468 (PMC8439445; doi:10.1371/journal.pone.0257468)
Supplement: S6 File — (PDF) [file pone.0257468.s006.pdf]

new foreign masks tested on particle counter setup	particle counter	Solar 3100	Supplemental file 6
	interface	particle chamber	www.misit.nl
	Flow	1cf/min	26 Liter/min @ 21 m/s
	measurement time	1 minute	autor T.Horeman / JvDobbelsteen / D Robertson

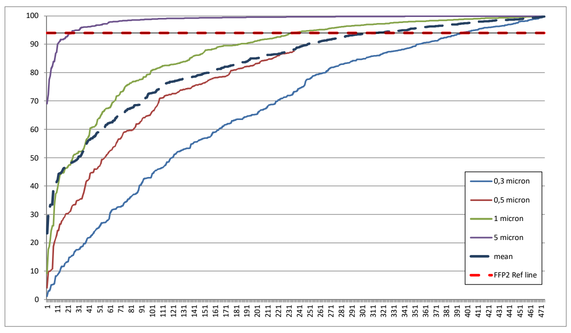

new masks total

Mask name	Mask type	Date	Number of Samples	percentage 0.3mu particles filtered [%]	percentage 0.5mu particles filtered [%]	percentage 1mu particles filtered [%]	percentage 5mu particles filtered [%]	MEAN [%]	Ordered from low to high: percentage 0.3mu particles filtered [%]	percentage 0.5mu particles filtered [%]	percentage 1mu particles filtered [%]	percentage 5mu particles filtered [%]	MEAN [%]	FFP2 filter level [%]	
chinese white no brand FFP2	FFP2	1-4-2020	4	77,7	89,2	94,8	99,9	90,4	1,19	4,18	9,22	69,10	23,35	94,00	1
polish MB20 NRD	FFP2	1-4-2020	3	83,4	94,7	98,3	99,3	93,9	3,02	9,66	17,65	72,34	29,81	94,00	2
China Holmar SD1069	FFP2	1-4-2020	2	95,0	98,9	99,3	100,0	98,3	3,02	10,03	18,68	77,59	30,07	94,00	3
disposable face mask	K95-A/FFP2	1-4-2020	2	42,8	73,8	91,1	100,0	76,9	3,68	10,03	21,09	78,81	33,37	94,00	4
white lable/unknown	FFP2	1-4-2020	2	5,4	10,5	17,7	86,8	30,1	5,13	10,46	24,20	81,42	33,53	94,00	5
GB2626-2006	FFP2 KN95	1-4-2020	2	78,9	91,7	96,2	99,9	91,7	5,25	10,83	26,04	82,40	33,53	94,00	6
white label	FFP2 KN95	1-4-2020	2	24,0	47,0	71,9	99,9	60,7	5,37	18,69	26,04	83,87	38,74	94,00	7
MoldexT model 8287	FFP2 KN95	1-4-2020	2	72,2	89,9	95,8	99,9	89,5	5,65	20,14	33,11	83,93	39,26	94,00	8
China KN95 FFP2	FFP2 KN96	30-3-2020	3	55,8	81,4	93,9	100,0	82,8	8,37	21,41	37,20	86,79	40,57	94,00	9
selfmade	selfmade OK doek/KN95	30-3-2020	3	37,2	62,0	81,3	98,8	69,8	8,37	22,69	37,65	87,23	41,37	94,00	10
selfmade	OK doek/KN95	30-3-2020	2	34,6	59,7	80,7	99,3	68,6	8,67	24,39	39,37	90,04	42,13	94,00	11
China KN95 FFP2	chinees EN149 2001 FFP2	30-3-2020	2	45,1	64,1	82,9	99,6	72,9	9,15	24,39	41,29	90,52	44,33	94,00	12
China GB2626-2006 KN96 FFP2	chinees EN149 2001 FFP2	30-3-2020	2	20,4	36,7	64,0	99,8	55,3	9,94	26,55	43,71	91,04	44,52	94,00	13
YUBAY	KN95 FFP2	30-3-2020	3	84,9	93,9	98,2	100,0	94,3	11,06	26,55	44,24	91,67	44,52	94,00	14
GROS	KN95	30-3-2020	3	90,1	95,1	97,9	99,9	95,7	11,74	27,80	44,68	91,67	45,02	94,00	15
DONG GUAN Wei sheng medical tech	ZF KN95 ffp2	30-3-2020	3	53,4	80,7	93,4	99,7	81,8	11,74	28,21	44,68	91,67	45,85	94,00	16
Shengzhen Tian Hai Test Tech	KN95 ffp2	30-3-2020	2	43,0	72,3	90,3	100,0	76,4	11,77	28,91	44,85	91,94	46,37	94,00	17
GB2626-2006	KN95	31-3-2020	4	67,1	82,3	91,9	100,0	85,3	12,69	29,28	46,08	92,02	46,44	94,00	18
arun KN95 FFP2	KN95 ffp2	31-3-2020	3	79,1	86,7	93,1	99,9	89,7	13,03	30,24	46,33	92,60	46,44	94,00	19
chirurgisch mondmasker china	KN95	31-3-2020	3	79,4	90,1	96,2	99,9	91,4	13,30	30,36	46,45	92,73	46,44	94,00	20
unknown china	KN95	31-3-2020	3	83,1	93,7	98,0	99,9	93,7	13,39	30,37	47,09	93,26	46,76	94,00	21
Honeywell 5209 FFP2 NRD D	FFP2	31-3-2020	2	87,6	92,0	95,2	99,1	93,5	14,91	30,73	47,45	93,70	47,54	94,00	22
M-safe 4210 FFP2 NR D	FFP2	31-3-2020	2	97,6	98,7	98,6	99,6	98,7	14,98	31,08	47,49	93,75	47,58	94,00	23
GB2626-2006 Putian Jinlilias clowthing weaving FFP2	FFP2	31-3-2020	2	45,6	68,1	86,0	99,9	74,9	15,28	32,13	48,84	93,82	48,33	94,00	24
mix and match N95 10 FFP2	FFP2	31-3-2020	2	34,7	59,7	80,9	99,5	68,7	15,51	33,04	49,42	94,73	48,36	94,00	25
AB Mund-Nasen N93 FFP2	FFP2	31-3-2020	2	9,2	18,7	37,2	92,0	39,3	15,66	33,40	50,67	94,77	48,61	94,00	26
Deltaplus brazil PFF2 (2)	PFF2	31-3-2020	1	90,7	96,5	99,1	100,0	96,6	16,87	33,45	51,25	94,84	48,72	94,00	27
Sayro PFF2-S CA	FFP2	31-3-2020	1	81,6	89,4	95,7	99,9	91,6	17,11	33,45	51,25	94,93	49,02	94,00	28
Romed	FFP2	31-3-2020	1	59,2	76,5	89,6	100,0	81,3	17,56	34,73	51,51	94,97	49,20	94,00	29
Spectrum MX-2005	FFP2	31-3-2020	2	96,6	97,4	98,2	99,8	98,0	17,56	34,95	51,51	95,02	49,63	94,00	30
Chinees hartje logoGB2626-2006 FFP2	FFP2	31-3-2020	2	84,9	92,7	97,3	100,0	93,7	17,70	35,07	52,22	95,02	50,44	94,00	31
noname china KN95 GB2626-2006	KN95	31-3-2020	2	82,1	90,7	96,5	99,7	92,2	17,81	35,30	52,26	95,18	50,53	94,00	32
MH Monarc heathcare GB2626-2006	KN95	31-3-2020	2	18,5	34,7	56,7	98,8	52,2	18,51	35,49	52,26	95,76	50,53	94,00	33
9591 gb2626-2006	FFP2	31-3-2020	2	97,0	98,5	99,3	100,0	98,7	18,76	35,56	52,26	95,97	51,38	94,00	34
love surprise	FFP2	31-3-2020	2	15,3	28,2	43,7	96,2	45,9	18,85	36,74	52,72	96,04	52,19	94,00	35
lianyou9501	FFP2	31-3-2020	2	95,4	98,4	99,5	100,0	98,3	19,75	37,75	53,19	96,05	52,27	94,00	36
Chicare GB2626-2006	FFP2	1-4-2020	3	68,7	82,5	90,0	98,9	85,0	19,78	39,80	54,70	96,08	54,67	94,00	37
white label KN95 FFP2	FFP2	1-4-2020	2	70,9	83,2	91,4	100,0	86,4	20,42	41,35	56,06	96,08	54,70	94,00	38
GB2626-2006 KN95 FFP2 NR D	FFP2	1-4-2020	3	91,1	96,2	98,4	99,8	96,4	21,27	41,96	56,74	96,15	55,25	94,00	39
ZF mark GB2626-2006 KN95 FFP2	FFP2	1-4-2020	1	60,4	87,6	95,2	98,0	85,3	21,72	42,05	57,13	96,22	55,94	94,00	40
white label	FFP2	1-4-2020	2	91,4	95,3	97,7	99,9	96,1	21,82	42,61	57,95	96,25	56,40	94,00	41
Chirurgisch mondmasker special fit	FFP2	1-4-2020	1	66,9	98,2	92,3	100,0	89,3	21,94	44,52	60,50	96,25	56,40	94,00	42
Chinees Ivrou	FFP2	1-4-2020	2	99,5	99,8	99,9	99,3	99,6	22,55	44,79	60,57	96,31	56,83	94,00	43
Fox med	FFP2	1-4-2020	3	86,0	94,1	97,7	96,2	93,5	22,88	44,79	60,75	96,40	56,89	94,00	44
COKO KN95	FFP2	3-4-2020	1	77,6	92,1	97,2	100,0	91,7	23,34	44,92	60,86	96,42	57,29	94,00	45
Holland House fashion No label	FFP2	3-4-2020	1	53,2	77,4	90,5	100,0	80,3	23,97	45,96	60,86	96,48	57,53	94,00	46
JSP Martcare 1020A FFP2 CE 0086	FFP2	3-4-2020	3	83,6	91,3	94,9	98,0	91,9	24,30	46,00	61,30	96,56	57,75	94,00	47
White label KN95 Cup	FFP2	3-4-2020	2	83,0	93,3	97,1	99,4	93,2	24,92	46,00	61,97	96,73	58,62	94,00	48
Mask FFP2 KN95	FFP2 & N95	3-4-2020	2	79,9	93,0	97,8	99,9	92,6	25,15	46,27	63,56	96,76	58,98	94,00	49
Mask FFP2 KN95	FFP2 & N95	3-4-2020	2	37,5	65,6	82,4	99,7	71,3	25,31	46,79	63,85	96,80	58,98	94,00	50
Lian You 9501 KN 95	KN 95	3-4-2020	1	96,8	99,3	99,8	99,9	99,0	25,94	46,98	64,04	96,81	59,12	94,00	51
Naisian 1981 KN 95	KN 95	3-4-2020	2	90,0	96,3	98,6	99,7	96,2	26,51	47,80	64,82	96,87	59,97	94,00	52
BESTG 8295 KN95	KN95	3-4-2020	2	94,7	97,1	98,1	99,8	97,4	27,05	49,36	65,41	96,87	59,99	94,00	53
Health ZK601 KN	KN 95	3-4-2020	2	95,5	98,7	99,6	99,1	98,3	27,05	49,45	66,16	97,17	60,15	94,00	54
FOX FFP2	FFP2	3-4-2020	2	85,7	95,0	98,3	99,3	94,6	27,17	49,72	66,43	97,24	60,68	94,00	55
mask (Blue)	FFP2	3-4-2020	1	74,4	89,0	95,7	99,7	89,7	27,18	50,18	66,96	97,27	60,82	94,00	56
Unbranded white cup	KN95	3-4-2020	2	97,4	99,4	99,8	99,5	99,0	27,26	50,54	67,29	97,27	61,61	94,00	57
Unknown PM2.5 KN 95	FFP2	3-4-2020	2	40,4	64,2	81,1	99,0	71,2	27,80	51,20	67,49	97,28	61,64	94,00	58
Jeliton KN95	FFP2	3-4-2020	2	87,6	95,4	98,1	99,4	95,1	28,09	51,50	67,49	97,60	62,18	94,00	59
No Marking	FFP2	3-4-2020	2	32,1	59,1	79,3	100,0	67,6	28,38	52,09	67,64	97,83	62,28	94,00	60
Unmarked	KN 95	3-4-2020	3	56,4	81,5	93,2	99,4	82,6	30,44	52,44	67,72	97,86	62,42	94,00	61
3M Aura 9322+ CE 2797	FFP2	3-4-2020	1	99,0	99,7	99,9	99,2	99,5	31,24	52,66	68,58	97,95	62,48	94,00	62
Puffin II a	FFP2	3-4-2020	2	75,6	92,4	97,5	99,1	91,2	31,65	52,98	69,51	97,96	62,54	94,00	63
Puffin II b	FFP2	3-4-2020	2	92,3	97,1	99,0	99,7	97,1	31,75	53,65	69,55	97,98	63,03	94,00	64
Bei Bei Safety B702 FFP2 CE0194 (new)	FFP2	3-4-2020	1	95,1	97,7	98,7	99,0	97,6	31,90	53,89	70,07	97,99	63,11	94,00	65
KN95 Mask 20200326 EN149	FFP2	3-4-2020	2	30,4	49,7	69,6	99,4	62,3	31,97	54,21	70,34	98,01	63,96	94,00	66
Lanshiorm KN96	FFP2	3-4-2020	1	97,0	98,8	99,3	99,5	98,7	32,13	55,18	71,71	98,03	64,15	94,00	67
S. Sango XG2020	FFP2	3-4-2020	1	70,4	84,8	93,8	99,2	87,0	32,59	55,70	71,85	98,04	64,68	94,00	68
Zhongshan Guangyuan Medical Instruments Tech	FFP2	3-4-2020	2	57,0	72,7	83,9	99,9	78,4	32,68	56,60	71,92	98,16	65,59	94,00	69
Aszorb	FFP2	6-4-2020	2	94,3	96,9	98,1	99,8	97,3	32,75	56,64	72,30	98,23	65,73	94,00	70
Jinlilaisi	FFP2	6-4-2020	2	22,9	44,9	67,3	99,4	58,6	32,75	56,69	73,24	98,24	65,74	94,00	71
FOX Med FFP2	FFP2	6-4-2020	2	83,3	93,3	96,5	82,4	88,9	32,99	57,38	73,30	98,30	65,86	94,00	72
Vision Textiles	FFP2	6-4-2020	1	46,6	80,0	92,4	98,0	79,2	33,04	58,23	73,39	98,35	65,90	94,00	73
Vision Textiles 3 layer surgical mask	FFP2	6-4-2020	2	59,0	82,4	93,3	99,0	83,4	33,80	58,95	74,02	98,35	65,95	94,00	74
Qingdao Weida	FFP2	6-4-2020	2	84,3	93,8	97,7	96,8	93,2	34,26	59,11	74,77	98,35	66,15	94,00	75
Caritol	FFP2	6-4-2020	2	91,2	96,6	98,9	99,9	96,7	34,56	59,18	75,40	98,36	66,61	94,00	76
Morntrip	FFP2	6-4-2020	2	74,8	88,8	95,7	99,9	89,8	34,69	59,60	75,54	98,37	66,77	94,00	77
FILL IN MASK INFO	FFP2	6-4-2020	1	38,6	74,6	90,1	94,7	74,5	35,47	59,60	75,79	98,45	67,22	94,00	78
GCPC	FFP2	6-4-2020	2	93,6	97,0	98,6	99,7	97,2	35,57	59,63	75,86	98,47	67,22	94,00	79
Zheijang	FFP2	6-4-2020	2	90,6	96,8	99,1	99,9	96,6	36,09	59,63	76,37	98,52	67,48	94,00	80
Prime Asia EN14683 Type 2	FFP2	6-4-2020	2	56,9	81,8	91,8	99,9	82,6	36,32	59,66	76,37	98,53	67,55	94,00	81
Prime Asia EN14683 Type 1	FFP2	6-4-2020	1	55,5	78,3	90,1	96,1	80,0	36,65	59,72	76,76	98,57	67,64	94,00	82
Prime Asia EN14683 Type 2R	FFP2	6-4-2020	1	65,2	82,2	92,6	100,0	85,0	37,05	59,95	76,88	98,57	67,83	94,00	83
Prime Asia YY/T 0969 single use	FFP2	6-4-2020	2	21,9	54,2	77,8	95,8	62,4	37,05	60,48	76,88	98,57	68,47	94,00	84
Vercom KN95 FFP2	FFP2	6-4-2020	2	85,5	94,7	98,2	99,8	94,5	37,24	60,77	76,98	98,57	68,56	94,00	85
Shuter Life CE Medical Mask	FFP2	6-4-2020	2	63,3	84,2	94,3	99,9	85,4	37,48	61,92	77,18	98,59	68,63	94,00	86
San Bang 9051A	FFP2	7-4-2020	2	87,8	94,6	97,7	99,8	95,0	37,73	61,92	77,28	98,62	68,63	94,00	87
Unknown	FFP2	7-4-2020	2	83,0	85,5	94,2	99,9	90,7	38,56	62,02	77,32	98,62	68,66	94,00	88
Unknown (no. 3)	FFP2	7-4-2020	1	84,7	94,6	98,4	99,9	94,4	40,28	62,14	77,57	98,63	68,70	94,00	89
San Bang 9300A (no. 4)	FFP2	7-4-2020	1	84,8	93,5	97,7	100,0	94,0	40,41	62,88	77,61	98,67	68,78	94,00	90
GB2 626-2006	FFP2	6-4-2020	2	68,9	84,2	91,9	99,7	86,2	41,04	63,00	77,74	98,71	69,84	94,00	91
Unknown surgical mask	FFP2	6-4-2020	2	15,7	28,9	46,4	99,1	47,5	41,64	63,92	77,76	98,75	69,98	94,00	92
SH2920V	FFP2	7-4-2020	2	93,0	97,9	99,3	98,0	97,1	42,51	64,15	78,46	98,75	70,10	94,00	93
SW1016	FFP2	7-4-2020	2	79,9	90,3	75,5	98,3	86,0	42,71	64,23	78,53	98,77	70,77	94,00	94
Unknown FFP2	FFP2	7-4-2020	2	18,9	39,8	60,7	99,3	54,7	42,75	64,64	78,53	98,78	71,21	94,00	95
Unknown FFP2	FFP2	7-4-2020	2	77,7	89,2	95,2	99,6	90,4	42,83	64,76	79,31	98,78	71,29	94,00	96
KN95 mask	FFP2	7-4-2020	2	32,6	49,4	67,0	99,7	62,2	42,98	64,87	79,39	98,83	72,43	94,00	97
Huate GB2626	FFP2	7-4-2020	2	94,8	98,1	99,2	99,9	98,0	43,00	64,93	79,43	98,83	72,65	94,00	98
SW1015	FFP2	7-4-2020	2	94,5	97,8	98,3	77,6	92,1	43,00	64,97	79,52	98,84	72,70	94,00	99
Unknown KN95	FFP2	7-4-2020	2	27,8	46,8	66,4	99,6	60,2	43,01	65,56	80,68	98,85	72,80	94,00	100
a.o.hope MT2010-4-2	FFP2	7-4-2020	2	84,7	93,9	97,8	99,6	94,0	44,46	66,35	80,92	98,87	72,82	94,00	101
RX Safety Products RX-DM-N2	FFP2	7-4-2020	2	88,3	92,5	94,7	99,2	93,7	44,50	66,62	81,07	98,88	72,93	94,00	102
Popal KN95	FFP2	7-4-2020	2	90,3	96,3	98,6	100,0	96,3	45,06	66,64	81,14	98,90	73,02	94,00	103
Unknown KN95 mask (witte band)	FFP2	7-4-2020	2	44,5	65,0	81,5	99,9	72,7	45,53	67,72	81,26	98,94	73,07	94,00	104
Unknown KN95 mask (grijze band)	FFP2	7-4-2020	2	49,9	71,3	85,3	99,2	76,4	45,60	68,11	81,47	98,95	73,57	94,00	105
Uvex silv-Air 5220+ (ongebruikt, ongesteriliseerd)	FFP2	7-4-2020	1	98,1	99,3	99,6	100,0	99,2	45,92	68,50	81,55	98,96	73,99	94,00	106
Unknown KN95 mask	FFP2	8-4-2020	2	25,1	42,6	60,5	99,3	56,9	46,00	69,21	81,69	98,99	74,16	94,00	107
Safe 10204	FFP2	8-4-2020	2	81,8	93,0	97,2	99,6	92,9	46,17	70,97	81,83	99,00	74,48	94,00	108
Ueacon KN95 B	FFP2	8-4-2020	2	11,1	46,0	72,3	99,8	57,3	46,20	71,06	82,01	99,00	74,65	94,00	109
Ueacon KN95 C	FFP2	8-4-2020	2	64,4	83,3	92,4	98,5	84,6	46,48	71,07	82,07	99,03	74,90	94,00	110
Chengheng	FFP2	8-4-2020	2	63,1	81,6	89,8	96,7	82,8	46,56	71,16	82,40	99,05	75,08	94,00	111
Maco Pharma ZZM002 (14.60, 10.60, 12.60 en 8.60 re	FFP2 and FFP3	9-4-2020	4	85,9	95,8	98,6	99,7	95,0	46,90	71,28	82,50	99,06	75,76	94,00	112
Unknown KN95 mask	FFP2	9-4-2020	2	93,3	97,1	98,8	100,0	97,3	47,28	71,80	82,52	99,07	75,77	94,00	113
My-T-Gear 302	FFP2	7-4-2020	2	88,0	92,5	94,7	98,9	93,5	47,52	71,98	82,52	99,10	76,17	94,00	114
Naqing Keji GB/T32610-2016 (N95)	FFP2	9-4-2020	2	92,4	98,5	99,2	100,0	97,5	48,44	72,07	82,52	99,12	76,38	94,00	115
Unknown KN95 mask	FFP2	9-4-2020	2	56,3	72,7	83,4	99,8	78,1	48,44	72,11	82,56	99,12	76,43	94,00	116
Celab	FFP2	9-4-2020	2	46,0	77,7	90,3	94,8	77,2	48,60	72,28	82,62	99,12	76,51	94,00	117
Antarctica (no. 2)	FFP2	9-4-2020	2	94,4	97,6	98,9	99,3	97,5	49,60	72,32	82,79	99,12	76,76	94,00	118
ESoundMed (no. 1)	FFP2	9-4-2020	2	96,2	98,5	99,3	99,6	98,4	49,91	72,34	82,90	99,13	76,80	94,00	119
Mukden i tanggin (no. 3)	FFP2	9-4-2020	2	85,9	94,5	98,1	100,0	94,6	50,12	72,60	82,94	99,13	76,82	94,00	120
Boldair AUU MP300V(SL)	FFP3	9-4-2020	2	99,4	99,7	99,8	99,0	99,5	50,15	72,67	82,94	99,14	76,89	94,00	121
Unknown FFP2 mask	FFP2	9-4-2020	2	90,7	97,2	99,2	99,8	96,7	51,19	72,68	82,98	99,15	76,94	94,00	122
Unknown KN95 mask	FFP2	7-4-2020	2	84,9	95,0	98,5	99,9	94,6	51,28	72,69	83,06	99,16	77,11	94,00	123
Caisen N95 mask	FFP2	7-4-2020	2	88,4	95,0	98,0	99,9	95,3	51,55	72,79	83,10	99,17	77,18	94,00	124
Zhongsshan Xinmeite Household Tech Co Ltd (Royal G	No certicifation	7-4-2020	2	71,5	86,8	94,6	99,9	88,2	52,08	73,11	83,28	99,17	77,23	94,00	125
Nuokang Medical KN95	FFP2	10-4-2020	2	95,2	98,1	99,2	99,9	98,1	52,21	73,23	83,34	99,17	77,38	94,00	126
Putian Jinlilais Clothing Weaving Co KN95	FFP2	10-4-2020	2	32,8	59,6	76,9	99,6	67,2	52,22	73,54	83,37	99,18	77,52	94,00	127
Unknown KN95 Mask	FFP2	10-4-2020	1	32,8	59,6	76,9	99,6	67,2	52,40	73,84	83,59	99,18	77,56	94,00	128
Gangshi KN95	FFP2	10-4-2020	2	94,8	98,6	99,4	99,9	98,2	52,66	73,87	83,72	99,18	77,57	94,00	129
Unknown KN95 Mask (groene letters)	FFP2	10-4-2020	1	70,9	85,9	93,9	99,7	87,6	52,73	73,99	83,84	99,19	77,80	94,00	130
Unknown KN95 Mask	FFP2	10-4-2020	1	31,9	55,2	75,4	99,9	65,6	53,06	74,07	83,88	99,20	77,90	94,00	131
KN95 CE0194	FFP2	10-4-2020	1	42,7	74,1	89,2	100,0	76,5	53,18	74,11	83,92	99,22	77,97	94,00	132
Gangshi KN95	FFP2	10-4-2020	1	96,0	98,7	99,4	100,0	98,5	53,27	74,31	84,08	99,24	77,98	94,00	133
Nuokang Medical KN95	FFP2	10-4-2020	1	97,2	99,0	99,7	99,9	98,9	53,32	74,31	84,20	99,25	78,05	94,00	134
Weihui 9801 GB2626-2006 KN95	FFP2	10-4-2020	1	40,3	75,3	92,0	99,7	76,8	53,33	74,49	84,41	99,26	78,05	94,00	135
Daylead KN95 FFP2	FFP2	10-4-2020	2	93,1	98,0	99,4	99,2	97,4	53,37	74,49	84,48	99,26	78,20	94,00	136
EN149-2001+A1.2009	FFP2	13-4-2020	2	79,3	91,9	97,1	99,9	92,1	53,43	74,50	85,17	99,27	78,29	94,00	137
Dazang Medical	KN95	12-4-2020	2	79,7	91,3	96,5	99,9	91,9	54,82	74,56	85,32	99,27	78,37	94,00	138
Protective Mask, groene verpakking FFP2	FFP2	12-4-2020	2	66,1	89,2	96,1	99,5	87,7	54,92	75,07	85,46	99,28	78,45	94,00	139
RYZUR	No certicifation	12-4-2020	2	98,3	98,8	99,2	99,9	99,0	55,01	75,27	85,88	99,30	78,58	94,00	140
JINJIANG - grijs -	No certicifation	7-4-2020	2	96,3	98,6	99,4	100,0	98,6	55,54	75,37	85,99	99,30	78,64	94,00	141
Anteo KN 95 Tiffany FFP2	FFP2	12-4-2020	2	93,3	96,8	98,5	99,4	97,0	55,56	75,44	86,01	99,31	78,65	94,00	142
								#DIV/0!	55,60	75,65	86,13	99,32	78,81	94,00	143
GB2626-2006 KN95 Unknown	FFP2/KN95	7-4-2020	2	90,1	81,5	98,0	100,0	92,4	55,77	75,65	86,19	99,32	79,11	94,00	144
BVD logo GB2626-2006 KN95 Unknown	FFP2/KN95	14-4-2020	1	92,2	96,9	98,8	99,9	96,9	56,07	75,73	86,19	99,32	79,13	94,00	145
IMG Europe FFP2	FFP2	14-4-2020	1	86,1	92,7	95,9	99,5	93,5	56,31	75,87	86,28	99,33	79,24	94,00	146
huisimei gb2626-2006	kn95	14-4-2020	1	78,4	89,1	95,2	99,5	90,6	56,37	75,91	86,47	99,33	79,25	94,00	147
China No logo no brand		15-4-2020	1	63,1	81,0	91,1	99,2	83,6	56,51	76,07	86,67	99,33	79,29	94,00	148
China KN 95		15-4-2020	1	96,3	98,4	99,0	99,2	98,2	56,71	76,13	86,70	99,33	79,51	94,00	149
chineese unknown KN95	FFP2	16-4-2020	2	33,0	59,6	77,7	99,9	67,6	56,85	76,41	87,15	99,34	79,72	94,00	150
chineese unknown KN95	FFP2	16-4-2020	2	26,5	49,4	67,7	99,7	60,8	56,94	76,52	87,58	99,34	79,81	94,00	151
chineese unknown KN95	FFP2	16-4-2020	2	86,1	92,0	94,6	99,7	93,1	56,95	76,60	88,00	99,34	79,89	94,00	152
Staal 1: Baltisse (Kangmin - (K)N95)	FFP2	16-4-2020	1	92,7	96,9	98,5	99,1	96,8	57,01	76,85	88,00	99,36	79,95	94,00	153
Staal 2: Overheid (Industrias saver S.A. - N95 - Life 109	FFP2	16-4-2020	1	94,2	97,3	98,1	100,0	97,4	57,17	77,23	88,10	99,36	79,95	94,00	154
Staal 3: Leger van Aarschot (Particulate Respirator - K	FFP2	16-4-2020	1	89,5	95,5	97,8	100,0	95,7	57,32	77,38	88,13	99,36	80,03	94,00	155
Staal 4: Vokla - Made for MBI (KN95)	FFP2	16-4-2020	1	41,0	62,1	77,3	99,9	70,1	57,32	77,63	88,15	99,37	80,18	94,00	156
Staal 5: Fluvia (KN95)	FFP2	16-4-2020	1	88,3	93,7	96,0	99,8	94,5	57,72	77,67	88,15	99,38	80,27	94,00	157
Staal 6: Dalian Wanli - Zhajian Allta Industry Co. LTD (K	FFP2	16-4-2020	1	80,0	90,1	95,2	100,0	91,3	58,45	77,89	88,37	99,38	80,27	94,00	158
Staal 7: Baltisse - Chenheng V9501 (KN95)	FFP2	16-4-2020	1	78,9	89,3	94,6	99,8	90,6	58,94	77,90	88,37	99,39	80,37	94,00	159
Sample B 11 Oud	FFP2	16-4-2020	2	66,8	82,7	92,6	99,7	85,4	58,97	78,16	88,45	99,39	80,74	94,00	160
Sample A nr Nieuw	FFP2	16-4-2020	2	73,3	87,5	94,9	99,8	88,9	59,12	78,32	88,49	99,40	80,79	94,00	161
Sample D	FFP2	16-4-2020	2	52,2	75,4	91,6	99,9	79,8	59,18	78,33	88,59	99,41	80,85	94,00	162
Sample C 11 Roud	FFP2	16-4-2020	1	63,7	84,6	92,9	99,7	85,2	59,70	78,33	89,04	99,41	81,25	94,00	163
KN95 Mask	FFP2	10-4-2020	1	78,1	91,9	96,6	99,9	91,6	59,75	78,35	89,10	99,41	81,34	94,00	164
Test 1		16-4-2020	1	86,5	93,5	97,0	100,0	94,2	60,24	78,45	89,19	99,41	81,35	94,00	165
Test 2		16-4-2020	1	82,4	91,8	24,2	98,2	74,2	60,42	78,52	89,22	99,43	81,35	94,00	166
Test 3		16-4-2020	1	96,6	99,0	99,7	99,9	98,8	60,57	78,55	89,31	99,43	81,66	94,00	167
Staal 1: unknown KN95	FFP2	16-4-2020	1	87,9	96,0	98,5	99,8	95,6	61,15	78,61	89,38	99,45	81,75	94,00	168
Staal 2: unknown NK95	FFP2	16-4-2020	1	86,7	95,1	98,0	100,0	94,9	61,17	78,61	89,54	99,45	81,78	94,00	169
Sample 1 (Fox Medical)	Chi II R	16-4-2020	1	52,1	72,3	83,1	99,6	76,8	61,57	78,61	89,55	99,46	82,09	94,00	170
Sample 1 (Fox Medical)	Chi II R	16-4-2020	1	46,2	66,3	78,5	99,5	72,6	61,72	78,70	89,56	99,47	82,12	94,00	171
Sample 1 (Fox Medical)	Chi II R	16-4-2020	1	46,9	66,6	77,6	98,6	72,4	61,81	78,96	89,57	99,47	82,12	94,00	172
Sample 2 (Firma Louter groen)	Chi II R	16-4-2020	1	51,2	74,0	84,5	98,8	77,1	62,03	79,12	89,58	99,48	82,16	94,00	173
Sample 2 (Firma Louter groen)	Chi II R	16-4-2020	1	52,4	75,7	86,2	99,5	78,5	62,03	79,37	89,59	99,48	82,30	94,00	174
chineese KN95	FFP2	16-4-2020	1	67,3	87,2	94,3	99,9	87,1	62,15	80,02	89,61	99,48	82,35	94,00	175
Sample 1 (Nuokang)	FFP II	16-4-2020	1	17,1	33,0	48,8	99,5	49,6	62,29	80,71	89,61	99,48	82,57	94,00	176
Sample 1 (Nuokang)	FFP II	16-4-2020	1	73,4	83,9	89,3	100,0	86,7	63,05	80,76	89,62	99,48	82,60	94,00	177
Sample 2 (Nuokang)	FFP II	16-4-2020	1	14,9	29,3	44,2	98,6	46,8	63,08	80,83	89,63	99,48	82,63	94,00	178
Sample 1 (Gangshi)	FFP II	16-4-2020	1	56,9	74,5	83,7	99,5	78,6	63,26	80,96	89,63	99,49	82,77	94,00	179
Sample 1 (Gangshi)	FFP II	16-4-2020	1	45,9	64,9	83,9	99,6	73,6	63,30	80,96	89,64	99,50	82,80	94,00	180
Sample 2 (Gangshi)	FFP II	16-4-2020	1	22,5	32,1	39,4	96,2	47,6	63,54	81,02	89,78	99,50	83,02	94,00	181
Virtue health 2020-1XG CE FFP2	FFP2/N95	21-4-2020	1	91,0	96,5	98,4	99,1	96,3	63,61	81,17	89,83	99,51	83,15	94,00	182
Sure & MeMN001-1 CE N95	FFP2/N95	21-4-2020	1	25,9	51,2	70,1	99,4	61,6	63,61	81,44	90,00	99,52	83,28	94,00	183
NAFY CEFFP2	FFP2	21-4-2020	1	55,6	72,1	82,9	99,5	77,5	63,64	81,49	90,06	99,52	83,40	94,00	184
medicom 2092_WN FFP2 IIR	FFP2	22-4-2020	2	93,0	97,9	99,0	99,9	97,5	63,65	81,54	90,13	99,53	83,59	94,00	185
self made OR dress	FFP2	22-4-2020	1	59,1	59,9	58,0	90,0	66,8	63,65	81,55	90,23	99,53	83,62	94,00	186
Face mask surgical green	FFP2	22-4-2020	1	65,4	85,3	94,9	99,6	86,3	63,66	81,62	90,24	99,54	83,67	94,00	187
Face mask surgical FOX med type IIR	FFP2	22-4-2020	1	51,3	72,3	88,1	98,5	77,6	64,06	81,79	90,26	99,54	83,68	94,00	188
1/FT	FFP2	22-4-2020	2	95,9	98,5	99,1	99,8	98,3	64,36	82,14	90,26	99,55	83,72	94,00	189
2/XL	FFP2	22-4-2020	2	27,2	58,2	77,3	96,1	64,7	64,44	82,14	90,27	99,55	83,79	94,00	190
3/HS - 1	FFP2	22-4-2020	2	46,2	78,5	92,0	99,8	79,1	64,53	82,20	90,27	99,55	83,97	94,00	191
3/HS - 2	FFP2	22-4-2020	2	46,5	75,1	88,1	99,8	77,4	64,69	82,20	90,42	99,56	84,20	94,00	192
4/ZY	FFP2	22-4-2020	2	61,2	80,8	90,6	99,6	83,0	64,74	82,21	90,53	99,56	84,49	94,00	193
5/TY	FFP2	22-4-2020	2	68,8	86,8	94,2	99,9	87,4	64,81	82,29	90,58	99,56	84,62	94,00	194
6/MF-D	FFP2	22-4-2020	1	99,9	99,6	99,1	98,8	99,3	64,89	82,41	90,71	99,57	85,01	94,00	195
7/MF-P	FFP2	22-4-2020	1	96,8	93,8	82,9	72,3	86,5	65,19	82,45	90,73	99,57	85,01	94,00	196
unknown	FFP3	15-4-2020	1	36,1	56,6	77,2	100,0	67,5	65,22	82,61	90,84	99,57	85,01	94,00	197
KN95 C GB2626-2006	FFP2	15-4-2020	1	82,0	95,5	99,2	100,0	94,2	65,23	82,67	90,95	99,57	85,05	94,00	198
TYPE 2	KN95	15-4-2020	1	51,5	76,4	90,9	100,0	79,7	65,28	83,08	91,14	99,58	85,07	94,00	199
Mondmasker bol wit B	FFP2	15-4-2020	1	5,6	27,8	56,1	96,3	46,4	65,28	83,23	91,14	99,58	85,10	94,00	200
, mondkapje wit	KN95	15-4-2020	1	11,8	30,7	50,7	69,1	40,6	65,37	83,26	91,30	99,58	85,16	94,00	201
FluCare Premium Earloop FM	KN95	15-4-2020	1	59,7	82,6	93,0	99,9	83,8	66,09	83,36	91,38	99,58	85,21	94,00	202
FFP2 A Wit HUI YOU	KN95	15-4-2020	1	88,5	95,7	98,3	99,4	95,5	66,43	83,36	91,40	99,58	85,31	94,00	203
FFP2 C Wit No Name	KN95	15-4-2020	1	96,1	98,6	99,4	99,9	98,5	66,79	83,93	91,62	99,58	85,31	94,00	204
9591 Wit PM2.5	KN95	15-4-2020	1	96,1	98,6	99,4	99,9	98,5	66,87	84,16	91,63	99,59	85,33	94,00	205
Lian You	KN95	15-4-2020	1	92,2	97,2	98,9	99,7	97,0	66,88	84,21	91,74	99,59	85,40	94,00	206
Love Surprise Wit	KN95	15-4-2020	1	5,2	10,8	21,1	96,3	33,4	67,15	84,23	91,74	99,59	85,44	94,00	207
Dispo Medical KN 95 HL	KN95	15-4-2020	1	86,1	93,5	97,2	100,0	94,2	67,26	84,41	91,77	99,59	85,93	94,00	208
Dispo Medical FFP2 SAFE 10204	KN95	15-4-2020	1	98,1	99,2	99,5	99,8	99,2	67,35	84,41	91,83	99,59	86,02	94,00	209
Dispo Medical KN 95 Ultra Sonic	KN95	15-4-2020	1	89,0	95,6	98,1	100,0	95,7	67,73	84,54	91,83	99,60	86,20	94,00	210
KN 95 Vokia	KN95	15-4-2020	1	61,7	76,6	86,7	100,0	81,3	68,21	84,63	91,91	99,60	86,30	94,00	211
health GB2626-2006 Zk601	KN95	15-4-2020	1	92,8	96,0	97,2	98,3	96,1	68,56	84,78	91,94	99,60	86,31	94,00	212
GB2626-2006 KN 95 met groene rand	KN95	15-4-2020	1	62,3	78,3	88,6	100,0	82,3	68,69	84,81	92,00	99,60	86,31	94,00	213
SUP Air Wit 23305	KN95	15-4-2020	1	96,3	98,9	99,6	99,9	98,7	68,75	84,83	92,00	99,61	86,31	94,00	214
3M 1861+	KN95	15-4-2020	1	81,3	89,7	94,8	99,9	91,4	68,77	84,94	92,03	99,61	86,39	94,00	215
Halyard Kimberley PFRP2-62408	KN95	15-4-2020	1	98,5	99,6	99,9	100,0	99,5	68,80	85,11	92,20	99,62	86,45	94,00	216
Blauw Chinese KN 95 0004-20200317	KN95	15-4-2020	1	88,8	93,9	96,8	99,6	94,8	68,92	85,30	92,28	99,63	86,49	94,00	217
Blauw AUMA COM Surgical Mask Sanjiau	KN95	15-4-2020	1	64,8	81,0	90,4	99,7	84,0	69,16	85,46	92,38	99,63	86,50	94,00	218
2-laags Membraan V2	KN95	15-4-2020	1	97,0	99,1	99,4	99,8	98,8	69,90	85,56	92,39	99,63	86,54	94,00	219
KN95 FFP2 YX011LAJANZHI	KN95	20-4-2020	6	86,9	94,2	97,3	99,9	94,6	69,90	85,94	92,57	99,63	86,54	94,00	220
Dental Air Wit	KN95	20-4-2020	4	91,2	97,7	99,2	99,9	97,0	70,35	86,10	92,57	99,64	86,67	94,00	221
Aersmash Wit	KN95	20-4-2020	2	63,6	81,0	89,8	100,0	83,6	70,54	86,11	92,62	99,64	86,73	94,00	222
SFG Face Mask N95 FFP2 2020KN95	KN95	20-4-2020	2	35,5	56,6	71,7	100,0	65,9	70,76	86,57	92,63	99,64	86,74	94,00	223
Freek Endtz SureTorq	KN95	20-4-2020	2	75,1	87,6	93,9	99,5	89,0	70,93	86,67	92,90	99,65	86,88	94,00	224
AND MAGPIE	KN95	20-4-2020	2	85,4	93,4	97,1	99,9	93,9	70,94	86,71	92,91	99,65	87,03	94,00	225
3M VFlex 1802 CE2797	KN95	22-4-2020	3	88,7	94,6	96,9	99,8	95,0	71,34	86,75	93,00	99,66	87,04	94,00	226
KN95 Wit GB7626-2006	KN95	22-4-2020	2	8,4	26,6	52,3	98,6	46,4	71,35	86,76	93,09	99,66	87,15	94,00	227
Asmtelring 100.000	KN95	22-4-2020	1	72,0	91,6	97,0	100,0	90,1	71,39	86,85	93,21	99,66	87,42	94,00	228
Asmtelring Sungo	KN95	22-4-2020	1	79,6	95,3	98,4	100,0	93,3	71,53	86,86	93,26	99,66	87,59	94,00	229
Maco Pharma ZZM002EUKO912 FFP2	KN95	22-4-2020	2	95,9	99,0	99,6	99,9	98,6	71,92	86,98	93,37	99,66	87,62	94,00	230

Zhengzhou Ruipy	KN95	22-4-2020	2	43,0	44,8	51,3	96,9	59,0	71,97	86,99	93,78	99,66	87,73	94,00	231
Winkel Han	KN95	22-4-2020	2	11,7	24,4	44,7	97,3	44,5	71,97	87,19	93,83	99,67	87,84	94,00	232
Groene doos KN95	KN95	22-4-2020	1	98,3	99,2	98,9	94,9	97,8	72,09	87,24	93,88	99,67	87,91	94,00	233
Bamboo	KN95	22-4-2020	1	65,3	83,4	91,7	100,0	85,1	72,21	87,24	93,88	99,67	87,98	94,00	234
Nomad	KN95	22-4-2020	1	84,3	94,3	97,9	100,0	94,1	72,76	87,48	93,93	99,68	88,03	94,00	235
RYZUR Groen masker	KN95	22-4-2020	1	98,7	98,9	97,4	93,8	97,2	73,30	87,57	94,06	99,68	88,21	94,00	236
GLL D	KN95	22-4-2020	1	48,4	75,7	88,4	99,7	78,1	73,34	87,64	94,21	99,68	88,26	94,00	237
KN95 Wit GB7626-2006	KN95	22-4-2020	2	8,369703155	26,55477672	52,26009831	98,56943475	46,4	73,42	88,14	94,24	99,68	88,66	94,00	238
Asmtelring 100.000	KN95	22-4-2020	2	71,96593173	91,55336732	96,96163923	100	90,1	73,62	88,30	94,26	99,68	88,86	94,00	239
Asmtelring Sungo	KN95	22-4-2020	2	79,59462907	95,25780218	98,35070881	100	93,3	74,36	88,77	94,29	99,68	88,89	94,00	240
Maco Pharma ZZM002EUKO912 FFP2	KN95	22-4-2020	2	95,92625642	99,00208942	99,55163401	99,89094875	98,6	74,44	89,00	94,47	99,69	89,01	94,00	241
Zhengzhou Ruipy	KN95	22-4-2020	2	42,99887197	44,78735048	51,25155639	96,87114953	59,0	74,67	89,11	94,47	99,70	89,22	94,00	242
Winkel Han	KN95	22-4-2020	2	11,73889636	24,39036583	44,67699225	97,27045494	44,5	74,80	89,15	94,57	99,70	89,33	94,00	243
Groene doos KN95	KN95	22-4-2020	1	98,29584068	99,23132439	98,92884468	96,08294931	98,1	75,06	89,19	94,60	99,70	89,46	94,00	244
Willson 9004	KN95	22-4-2020	1	90,01003718	96,9413355	98,6993114	99,8156682	96,4	75,17	89,20	94,61	99,70	89,58	94,00	245
Bamboo	KN95	22-4-2020	1	65,27590354	83,36178159	91,74268731	99,8156682	85,0	75,62	89,26	94,68	99,71	89,70	94,00	246
Nomad	KN95	22-4-2020	1	84,2946803	94,30538531	97,88123124	99,95391705	94,1	76,97	89,37	94,68	99,71	89,71	94,00	247
RYZUR Groen masker	KN95	22-4-2020	1	98,68220178	98,85672391	97,35742452	94,83870968	97,4	77,57	89,52	94,73	99,71	89,79	94,00	248
GLL D	KN95	22-4-2020	1	48,44229271	75,65096858	88,3703137	99,44700461	78,0	77,69	89,66	94,75	99,71	90,12	94,00	249
Leishide PM2.5	KN95	24-4-2020	2	85,63755582	93,19661006	96,32818713	99,87304274	93,8	77,72	89,83	94,81	99,71	90,12	94,00	250
Juyie surgical mask	KN95	24-4-2020	2	61,5717427	77,88791399	86,27800477	99,66144731	81,3	77,84	89,89	94,85	99,71	90,39	94,00	251
Face mask KN95-A FFP2 NR	KN95	24-4-2020	2	72,76311577	86,86291693	92,56862369	99,91536183	88,0	78,13	90,04	94,87	99,71	90,44	94,00	252
Halipo surgical mask 200c IIR	KN95	24-4-2020	2	57,72079094	73,54137973	81,83485873	99,71045965	78,2	78,44	90,08	94,88	99,72	90,47	94,00	253
Unifree surgical mask 100a IIR	KN95	24-4-2020	2	31,23557691	64,63564413	79,38625351	98,62468332	68,5	78,89	90,09	94,90	99,72	90,50	94,00	254
Si Ji JIE 500Cz	KN95	24-4-2020	2	97,43320052	99,45041506	99,62010692	99,82449982	99,1	78,90	90,18	94,94	99,72	90,54	94,00	255
KIASHI FFP2 KN95	KN95	24-4-2020	2	14,97588591	35,29841163	52,22354945	99,26289926	50,4	79,11	90,27	94,97	99,72	90,56	94,00	256
Ultrasonic CE FFP2 EN149	KN95	24-4-2020	2	87,01641739	94,26968565	96,8672293	99,90710636	94,5	79,25	90,30	94,97	99,73	90,62	94,00	257
Safety Armor Swam FFP2	KN95	24-4-2020	2	63,53508238	78,33216879	85,1676536	99,94949495	81,7	79,38	90,69	95,07	99,73	90,64	94,00	258
Swam 2 Lei.SHI.DE	KN95	24-4-2020	2	63,26446702	79,36716184	86,13012425	99,87277354	82,2	79,46	91,29	95,13	99,73	90,66	94,00	259
KN95 Respiratoir Zsen	KN95	24-4-2020	2	88,2668034	95,20574307	97,58955224	99,83940043	95,2	79,59	91,34	95,17	99,73	90,95	94,00	260
KN95 Varnax	KN95	24-4-2020	2	54,81549252	74,06809758	83,27919734	99,71910112	78,0	79,59	91,38	95,18	99,74	91,01	94,00	261
Weiban Mask KN95 (Cupola)	KN95	24-4-2020	2	55,60054095	78,33044648	87,58432351	99,95629371	80,4	79,71	91,53	95,20	99,74	91,15	94,00	262
daily respirator	KN95	28-4-2020	2	5,128120638	20,143694	46,0785433	97,16805942	42,1	79,86	91,55	95,21	99,74	91,29	94,00	263
merk 1 JarRouf code ADC HV	KN95	30-4-2020	2	3,01995149	10,02535023	26,04369761	95,0195988	33,5	79,94	91,55	95,21	99,74	91,30	94,00	264
merk 2 KN95 merk petra ADC HV	KN95	30-4-2020	2	98,09536954	99,38821273	99,74977298	100	99,3	79,97	91,73	95,24	99,75	91,36	94,00	265
merk 3 KN95 Frank SH	KN95	30-4-2020	2	17,56469806	33,45082745	51,51233852	99,58677686	50,5	80,13	91,82	95,31	99,75	91,40	94,00	266
merk 4 KN95 Frank BL 9186073	KN95	30-4-2020	2	63,65285425	74,31229531	82,52482617	99,31949643	80,0	80,62	91,88	95,31	99,75	91,42	94,00	267
merk 5 Disp.BERNTH	KN95	30-4-2020	2	69,90166943	84,40832172	91,83132481	100	86,5	80,67	91,90	95,44	99,76	91,64	94,00	268
merk 6 Disp.GRTJ	KN95	30-4-2020	2	37,05335277	61,92463253	76,37079179	99,18339571	68,6	81,25	91,90	95,58	99,76	91,65	94,00	269
shinzen Horb Technology	KN95	29-4-2020	2	53,05842024	70,96689656	79,43232637	99,58402662	75,8	81,56	91,91	95,60	99,77	91,69	94,00	270
city sparkling	KN95	29-4-2020	2	21,82468989	51,49575128	67,48719174	99,16804403	60,0	81,82	91,99	95,69	99,77	91,72	94,00	271
Lian You hartje logo	KN95	29-4-2020	2	69,16100811	86,66983427	94,96504623	99,57366541	87,6	81,86	92,00	95,73	99,77	91,86	94,00	272
RIPE	KN95	29-4-2020	2	93,56935703	95,58501232	98,46320223	99,75968759	96,8	81,94	92,04	95,74	99,77	91,90	94,00	273
tomson newt FFP2	KN95	29-4-2020	2	24,91559849	52,97581232	75,85933967	98,35251046	63,0	82,03	92,09	95,79	99,77	91,95	94,00	274
SPRO medical FFP2	KN95	29-4-2020	2	97,08515741	99,24937576	99,69727548	99,81424149	99,0	82,08	92,10	95,88	99,77	92,06	94,00	275
SPRO medical FFP2	KN95	29-4-2020	2	96,91606372	99,37483254	99,79315225	99,97552619	99,0	82,22	92,36	95,89	99,78	92,07	94,00	276
S.Sango (1) FFP2 CE2703	KN95	29-4-2020	2	56,93983764	73,22987848	83,33711691	99,63833635	78,3	82,34	92,46	96,00	99,78	92,23	94,00	277
Soliclean FFP2	KN95	29-4-2020	2	98,80457737	99,46659039	99,61256148	99,87944545	99,4	82,40	92,46	96,01	99,78	92,26	94,00	278
technotrading protective mask (3) FFP2	KN95	29-4-2020	2	57,17289491	72,67320022	82,06676576	99,69670711	77,9	82,73	92,50	96,07	99,78	92,40	94,00	279
Gh kn95 (4) FFP2	KN95	29-4-2020	2	85,81000114	93,36861284	96,41861761	99,56672444	93,8	82,90	92,53	96,14	99,79	92,42	94,00	280
soliclean (5) FFP2	KN95	29-4-2020	2	83,28336968	92,73308418	95,9959123	99,78336222	92,9	82,96	92,54	96,21	99,79	92,50	94,00	281
merk 1 JarRouf code ADC HV	KN95	30-4-2020	1	3,01995149	10,02535023	26,04369761	95,0195988	33,5	83,05	92,68	96,21	99,79	92,56	94,00	282
merk 2 KN95 merk petra ADC HV	KN95	30-4-2020	1	98,09536954	99,38821273	99,74977298	100	99,3	83,05	92,72	96,22	99,80	92,63	94,00	283
merk 3 KN95 Frank SH	KN95	30-4-2020	1	17,56469806	33,45082745	51,51233852	99,58677686	50,5	83,28	92,73	96,30	99,80	92,65	94,00	284
merk 4 KN95 Frank BL 9186073	KN95	30-4-2020	1	63,65285425	74,31229531	82,52482617	99,31949643	80,0	83,31	92,97	96,33	99,80	92,83	94,00	285
merk 5 Disp.BERNTH	KN95	30-4-2020	1	69,90166943	84,40832172	91,83132481	100	86,5	83,40	92,99	96,42	99,80	92,93	94,00	286
merk 6 Disp.GRTJ	KN95	30-4-2020	1	37,05335277	61,92463253	76,37079179	99,18339571	68,6	83,43	93,01	96,43	99,80	92,95	94,00	287
chinese KN95 FFP2	KN95	30-4-2020	2	8,667972513	22,68884666	37,65223095	96,47914275	41,4	83,47	93,04	96,46	99,81	93,10	94,00	288
TH2210 chinese KN95 FFP2	KN95	30-4-2020	2	90,69853533	96,15226125	98,04613106	99,87004548	96,2	83,63	93,14	96,48	99,81	93,16	94,00	289
konin med chinese KN95 FFP2	KN95	30-4-2020	2	86,26172058	93,04250915	95,3093038	95,97141001	92,6	83,95	93,16	96,50	99,81	93,17	94,00	290
J&C KN95 FACE mask FFP2 (J&C #1)	KN95	30-4-2020	2	98,63071525	99,33901682	99,58809975	99,96597482	99,4	84,01	93,16	96,50	99,81	93,21	94,00	291
J&C KN95 protective mask FFP2 (J&C #2)	KN95	30-4-2020	2	56,06738319	71,98175242	82,61862523	99,62572304	77,6	84,28	93,19	96,52	99,82	93,30	94,00	292
Bowin Disp.protective mask (surgical)	KN95	30-4-2020	2	90,45363585	97,55137338	98,98439204	99,32595089	96,6	84,29	93,20	96,55	99,82	93,30	94,00	293
Airtao AM02-KN95 RM	KN95	30-4-2020	2	95,62161043	98,08250101	98,86393821	99,3556182	98,0	84,29	93,27	96,63	99,82	93,39	94,00	294
divers	KN95	30-4-2020	2	47,28465386	71,0705245	83,59352824	98,36206897	75,1	84,30	93,27	96,64	99,82	93,43	94,00	295
divers	KN95	30-4-2020	6	52,66196415	72,59553212	82,7890174	99,51637674	76,9	84,71	93,29	96,64	99,82	93,48	94,00	296
divers	KN95	30-4-2020	10	48,59582378	66,6414913	77,57277545	99,45166857	73,1	84,72	93,37	96,69	99,83	93,50	94,00	297
Medline, None24508, FFP2 NR-EN149	KN95	1-5-2020	2	94,9579823	97,87411871	99,04809472	99,97941963	98,0	84,72	93,38	96,70	99,83	93,53	94,00	298
Moldex, 3255, FFP3 NR D-EN149:2001 CE0121	KN95	1-5-2020	2	68,5627184	85,106468	91,29740519	98,75152253	85,9	84,74	93,39	96,72	99,83	93,54	94,00	299
KN95 mask LMX-520	KN95	1-5-2020	2	13,02525491	31,08293834	53,18528195	99,51122375	49,2	84,77	93,40	96,75	99,83	93,63	94,00	300
DR core+ GB2626-2006 KN95 model 9550	KN95	1-5-2020	2	85,91009521	93,18981693	96,6391058	99,92661448	93,9	84,86	93,51	96,78	99,83	93,64	94,00	301
Health GB2626-2006 ZK601 KN95	KN95	1-5-2020	2	96,41849647	97,14043993	97,55187371	99,00435594	97,5	84,90	93,52	96,79	99,84	93,66	94,00	302
Standard GB2626-2006	KN95	1-5-2020	2	94,37432857	97,48684736	98,58327023	99,89633725	97,6	84,90	93,53	96,87	99,84	93,68	94,00	303
KN95 CE:EN149: 2001+A1 : 2009	KN95	1-5-2020	2	64,5295453	80,83250657	89,37515395	99,92862241	83,7	84,95	93,71	96,93	99,84	93,72	94,00	304
Disposable face mask 3ply type II	KN95	1-5-2020	2	33,79583527	52,4433844	67,4916897	98,70786517	63,1	85,39	93,73	96,96	99,84	93,76	94,00	305
Sandel FFP2 NR - 95002 EN149:2001 + A1 :2009 CE008	KN95	1-5-2020	2	91,73895315	96,81831022	98,65379731	100	96,8	85,41	93,78	96,96	99,84	93,79	94,00	306
Sandel FFP2 NR - 95002 EN149:2001 + A1 :2009 CE008	KN95	1-5-2020	2	89,2070536	95,73924418	97,98768839	99,90163934	95,7	85,47	93,80	96,98	99,84	93,89	94,00	307
Meixin MX-2005 EN 149:2001+A1:2009 CE0086	KN95	1-5-2020	2	98,94046997	99,69247624	99,6461166	96,04247104	98,6	85,64	93,83	97,05	99,84	93,92	94,00	308
JSP Martcare 1020A FFP2 EN149:2001	KN95	1-5-2020	1	92,38652503	96,54283514	98,18279256	98,34710744	96,4	85,71	93,85	97,05	99,85	93,93	94,00	309
JSP Martcare 1020A FFP2 EN149:2001 (Mask 2)	KN95	1-5-2020	1	87,45862214	94,10524522	97,20234223	100	94,7	85,81	93,86	97,06	99,85	93,95	94,00	310
Meixin MX-2005 EN 149:2001+A1:2009 CE0086 (Mask	KN95	1-5-2020	1	98,96799184	99,34889912	99,37927356	97,28033473	98,7	85,87	93,92	97,06	99,85	93,97	94,00	311
RYK 3D GB2626-2006	KN95	1-5-2020	2	96,01595089	99,6579526	99,77442166	99,8661149	98,8	85,91	94,05	97,11	99,85	93,98	94,00	312
MODEL HY001 Type HY-06.1	KN95	1-5-2020	2	64,69032642	81,49458354	90,83857842	99,79029359	84,2	85,93	94,08	97,15	99,85	94,00	94,00	313
Model HY001 Type HY-02	KN95	1-5-2020	2	56,71144256	76,12947511	88,1482157	99,74017876	80,2	85,98	94,09	97,16	99,85	94,09	94,00	314
Model HY001 Type HY-06.2	KN95	1-5-2020	2	18,75601174	42,04916098	63,84767557	99,121243	55,9	86,06	94,10	97,20	99,86	94,11	94,00	315
Tomson Newt KN95	KN95	6-5-2020	2	90,82559952	95,93573086	97,80156052	99,4100295	96,0	86,06	94,11	97,21	99,86	94,12	94,00	316
tomson Newt KN95 (mask 2)	KN95	6-5-2020	2	90,62207811	95,83230596	97,93409541	99,61439589	96,0	86,10	94,20	97,22	99,86	94,15	94,00	317
Chirurgisch Masker 2R Zo Gear	KN95	7-5-2020	2	42,70879175	62,88037189	74,77427354	99,56521739	70,0	86,26	94,27	97,23	99,86	94,18	94,00	318
KN95 Songzhe (1)	KN95	7-5-2020	2	64,43882323	78,45140335	86,47301313	99	82,1	86,48	94,31	97,23	99,86	94,19	94,00	319
FFP2 Shenzen Leihuo (2)	KN95	7-5-2020	2	52,20806282	67,71745216	76,75738007	99,27404719	74,0	86,65	94,31	97,26	99,86	94,20	94,00	320
KN95 Hwaxia (3)	KN95	7-5-2020	2	66,88200478	84,77649022	91,99813639	96,4	85,0	86,85	94,43	97,28	99,87	94,24	94,00	321
3-ply Face mask class 1 U-tech (4)	KN95	7-5-2020	2	53,33101682	68,50160076	78,53227861	98,2428115	74,7	87,02	94,50	97,28	99,87	94,26	94,00	322
3-ply Medical face mask class II -Wuxian Kangje (5)	KN95	7-5-2020	2	56,51177043	73,1087485	82,50341469	99,05660377	77,8	87,07	94,52	97,36	99,87	94,40	94,00	323
KN95 DNEI ME-KN950I (7)	KN95	7-5-2020	2	73,62265761	86,09544115	92,20164965	100	88,0	87,13	94,60	97,36	99,87	94,46	94,00	324
Self Made	KN95	15-4-2020	2	13,30126101	30,3579498	52,25600056	81,4159292	44,3	87,25	94,61	97,38	99,87	94,50	94,00	325
KN95 Staat &Co3	KN95	7-5-2020	2	77,83559786	88,14000504	92,62009419	99,73591549	89,6	87,45	94,61	97,39	99,87	94,52	94,00	326
KN95 Staat &Co4	KN95	7-5-2020	2	21,26860102	34,95010854	44,85017626	93,82022472	48,7	87,46	94,67	97,45	99,87	94,54	94,00	327
KN95 Staat &Co3 (Sterilized)	KN95	7-5-2020	2	53,31506049	71,16055181	79,52139054	99,06976744	75,8	87,46	94,67	97,52	99,87	94,55	94,00	328
KN95 Staat &Co4 (Sterilized)	KN95	7-5-2020	2	15,51217732	33,40396018	47,49188258	98,03001876	48,6	87,49	94,75	97,55	99,87	94,57	94,00	329
FFP-2	KN95	7-5-2020	2	64,06403491	81,17433968	88,44742701	99,42857143	83,3	87,56	94,98	97,59	99,87	94,58	94,00	330
Civil Mask	KN95	7-5-2020	2	60,24185554	75,90616386	84,07980456	99,3442623	79,9	87,63	95,03	97,68	99,87	94,59	94,00	331
GB262-2006KN95	KN95	7-5-2020	2	64,73794531	88,29735533	95,07373091	100	87,0	87,65	95,04	97,71	99,88	94,64	94,00	332
KN95 GB2626-2006	KN95	7-5-2020	2	61,14692681	82,21305245	90,73384961	98,51851852	83,2	87,75	95,06	97,71	99,88	94,67	94,00	333
GB2626-2006KN95	KN95	7-5-2020	2	58,94106122	77,63009169	87,15321717	99,66216216	80,8	87,80	95,12	97,72	99,88	94,69	94,00	334
1003 2626-2006KN95	KN95	7-5-2020	2	65,19435296	78,6973125	86,18925193	99,31623932	82,3	87,89	95,15	97,74	99,88	94,74	94,00	335
243 S5	KN95	7-5-2020	2	21,71642309	41,9605744	57,12844488	98,00724638	54,7	88,02	95,21	97,78	99,88	94,75	94,00	336
NRW Test Laurens (244 S1)	KN95	7-5-2020	2	84,72166394	92,4573651	94,9720914	91,66666667	91,0	88,02	95,26	97,78	99,88	94,84	94,00	337
2EX Test Laurens (244 S2)	KN95	7-5-2020	2	68,21062647	82,13627882	88,12926507	99,46808511	84,5	88,15	95,26	97,80	99,88	94,91	94,00	338
Nieuw Test Laurens (244 S3)	KN95	7-5-2020	2	87,74854881	93,26662782	94,74979822	98,75	93,6	88,25	95,29	97,81	99,88	94,94	94,00	339
Nieuw Test Laurens (244 S4)	KN95	7-5-2020	2	41,64207391	69,21451206	81,07105924	99,33510638	72,8	88,27	95,32	97,88	99,89	94,94	94,00	340
Nieuw Test Zellingen (244 S5)	KN95	7-5-2020	2	95,63386752	98,52528005	99,1225617	98,67374005	98,0	88,27	95,34	97,88	99,89	94,99	94,00	341
2e X test Pelie (244 S6)	KN95	7-5-2020	2	45,53337542	63,91617773	74,01995197	99,59785523	70,8	88,41	95,36	97,91	99,89	94,99	94,00	342
2e x X (244 S7)	KN95	7-5-2020	2	36,65226521	63,00251706	75,7865054	99,2	68,7	88,52	95,37	97,93	99,89	95,01	94,00	343
Personeel (244 S8)	KN95	7-5-2020	2	82,34477351	92,96903348	96,21089159	99,79757085	92,8	88,63	95,38	97,94	99,89	95,11	94,00	344
X (244 S9)	KN95	7-5-2020	2	89,34398315	95,15293636	96,72416076	83,92857143	91,3	88,65	95,39	97,98	99,89	95,23	94,00	345
Test nieuw argos (244 S10)	KN95	7-5-2020	2	76,97130644	85,56481455	89,09775673	99,71590909	87,8	88,78	95,47	97,99	99,89	95,33	94,00	346
Nieuw Test Laurens (244 S11)	KN95	7-5-2020	2	33,04128231	64,76237792	78,46246006	98,84615385	68,8	88,84	95,49	97,99	99,89	95,50	94,00	347
Test 2e keer sterilisatie naamloos (244 S12	KN95	7-5-2020	2	23,34000258	46,2739407	61,2988626	96,41509434	56,8	88,97	95,52	98,00	99,89	95,51	94,00	348
Test nieuw Vivia 2 (244 S13)	KN95	7-5-2020	2	55,0115931	82,14162682	90,23619229	99,26315789	81,7	89,21	95,56	98,04	99,89	95,56	94,00	349
Nieuw Test Bussum 3 (244 S14)	KN95	7-5-2020	2	70,76277601	86,10557769	91,39596974	99,25093633	86,9	89,33	95,59	98,05	99,90	95,56	94,00	350
Afheur +2 Vivvum groep (244 S15)	KN95	7-5-2020	2	35,57005715	58,94529694	71,92237631	100	66,6	89,34	95,74	98,07	99,90	95,62	94,00	351
2e Test Aafje (244 S16)	KN95	7-5-2020	2	87,13463078	93,38288338	95,58490455	99,45799458	93,9	89,55	95,74	98,08	99,90	95,65	94,00	352
Nieuw Test Argos (244 S17)	KN95	7-5-2020	2	31,6514077	59,17628082	73,38503861	99,375	65,9	89,96	95,81	98,10	99,90	95,71	94,00	353
2e x Test Aafje (244 S18)	KN95	7-5-2020	2	84,27535535	91,52825706	93,82878972	99,4140625	92,3	90,01	95,83	98,12	99,90	95,71	94,00	354
2e x test Aafje (244 S19)	KN95	7-5-2020	2	82,8971775	89,51945552	91,6152768	98,4496124	90,6	90,08	95,87	98,12	99,90	95,74	94,00	355
Evri Moya R302-mkp2sl	KN95	10-4-2020	2	94,67290302	96,60706493	97,0451456	93,70026525	95,5	90,11	95,94	98,14	99,90	95,79	94,00	356
Nikki 6502	KN95	10-4-2020	2	92,70951122	95,36066007	97,38730398	99,79977117	96,3	90,20	95,97	98,14	99,90	95,86	94,00	357
On Point 5010	KN95	10-4-2020	2	60,57436375	86,98875935	95,31067902	98,36575875	85,3	90,35	95,99	98,17	99,90	95,99	94,00	358
6200 Greenline	KN95	10-4-2020	2	87,48843501	94,09209136	97,27932696	99,83314794	94,7	90,45	96,03	98,17	99,91	96,00	94,00	359
QSA	KN95	10-4-2020	2	94,24607208	95,381536	98,32463946	100	97,0	90,62	96,03	98,18	99,91	96,07	94,00	360
Airmax	KN95	10-4-2020	2	98,07589885	98,79747534	99,02481093	98,62227325	98,6	90,63	96,04	98,19	99,91	96,08	94,00	361
1206 GB2626-2006KN95	KN95	8-5-2020	2	96,02222749	98,68667388	99,24687185	99,75609756	98,4	90,70	96,15	98,22	99,91	96,15	94,00	362
8014 GB2626-2006KN95	KN95	8-5-2020	2	91,92592518	97,11253383	98,41136185	100	96,9	90,72	96,23	98,22	99,91	96,19	94,00	363
IMD	IMD DM9501 GB2626-2006KN95	8-5-2020	2	57,31982977	76,06874564	83,84166986	99,77876106	79,3	90,73	96,29	98,26	99,91	96,25	94,00	364
LMX-520	KN95 Mask LMX-520 FFP2	8-5-2020	2	17,70185259	35,48720552	46,33495507	96,5625	49,0	90,83	96,31	98,32	99,92	96,28	94,00	365
Ref. MJH ZSUC	KN95 Mask	8-5-2020	2	73,29659402	92,10198807	96,48084581	100	90,5	90,90	96,36	98,32	99,92	96,30	94,00	366
RF-200K	KN95 Mask	11-5-2020	2	64,88868885	75,87102186	82,51546025	99,86702128	80,8	91,00	96,38	98,33	99,92	96,31	94,00	367
R2GO RF-200	KN95 Mask	11-5-2020	2	67,35186157	77,90105529	85,45559782	99,55919395	82,6	91,10	96,54	98,33	99,92	96,33	94,00	368
MASCARF R2GO	KN95 Mask	11-5-2020	2	1,186782484	4,179386682	9,217566678	78,80690738	23,3	91,13	96,54	98,35	99,92	96,36	94,00	369
V0 Zero Dr. K Mask	KN95 Mask	11-5-2020	2	12,69183627	30,37187868	47,45029719	94,96644295	46,4	91,20	96,55	98,35	99,92	96,37	94,00	370
MASCARF R2GO Fitted with R2GO RF-200	KN95 Mask	11-5-2020	2	62,14862457	73,87395349	81,5531281	98,87640449	79,1	91,23	96,61	98,37	99,93	96,40	94,00	371
KN95 Mask	KN95 Mask	11-5-2020	2	84,94757622	95,31857102	98,22195796	99,87179487	94,6	91,23	96,64	98,38	99,93	96,42	94,00	372
KN95 Mask kids	KN95 Mask	11-5-2020	2	83,94912561	93,13596664	96,63475592	99,85358712	93,4	91,35	96,80	98,41	99,93	96,44	94,00	373
KN95 Janlilaisi / Tab 2	KN95 Mask	11-5-2020	2	28,37550575	52,66470909	69,50658735	99,37565036	62,5	91,74	96,81	98,43	99,93	96,50	94,00	374
Tobrix / Tab 10	KN95 Mask	11-5-2020	2	34,25938084	53,89225595	68,57939144	99,09638554	64,0	91,93	96,82	98,44	99,93	96,58	94,00	375
ZWI Disposables / Tab 23	KN95 Mask	11-5-2020	2	84,00910227	93,16484779	96,70063744	99,85380117	93,4	92,17	96,85	98,45	99,93	96,58	94,00	376
Foppen / Tab 28	KN95 Mask	11-5-2020	2	71,3394092	84,23208466	90,71232559	99,67105263	86,5	92,17	96,94	98,46	99,93	96,59	94,00	377
Medica / Tab 29	KN95 Mask	11-5-2020	2	87,06779411	95,29000009	97,93618492	99,47552448	94,9	92,20	96,94	98,46	99,93	96,66	94,00	378
Tomson Newt KN95 (Mask 1 from test #236)	KN95 Mask	11-5-2020	2	96,2473888	98,92673822	99,3829483	99,84076433	98,6	92,33	96,94	98,47	99,93	96,74	94,00	379
Tomson Newt KN95 (Mask 2 from test #236)	KN95 Mask	11-5-2020	2	96,47164894	98,99466827	99,45623342	99,65437788	98,6	92,38	97,00	98,48	99,93	96,80	94,00	380
Tomson Newt FFP2 (Mask from test #216)	KN95 Mask	11-5-2020	2	31,96684942	56,68749569	70,33990041	97,60383387	64,1	92,39	97,06	98,49	99,93	96,81	94,00	381
Civil Mask Medi Care	KN95 Mask	11-5-2020	2	84,71400183	94,74754114	97,15913619	99,72826087	94,1	92,64	97,06	98,58	99,93	96,82	94,00	382
Civil Mask Jie Kang	KN95 Mask	11-5-2020	2	19,78108404	41,34962111	54,70427817	93,25842697	52,3	92,71	97,10	98,59	99,93	96,84	94,00	383
Civil Mask Vshield	KN95 Mask	11-5-2020	2	19,75074658	35,55769085	47,08627546	91,03671706	48,4	92,71	97,11	98,60	99,94	96,86	94,00	384
KN95 PMWhite	KN95 Mask	11-5-2020	2	88,83978774	98,01907872	99,22959092	99,65831435	96,4	92,76	97,11	98,63	99,94	96,95	94,00	385
Sunsmed Protective Civil Mask	KN95 Mask	11-5-2020	2	27,17508812	53,65163148	65,40803898	83,87096774	57,5	93,04	97,14	98,63	99,94	96,99	94,00	386
Nieuw "Eendenbek"	KN95 Mask	11-5-2020	2	96,34701213	99,30880366	99,75762917	99,79674797	98,8	93,04	97,18	98,64	99,94	96,99	94,00	387
Nieuw "Eendenbek"	KN95 Mask	11-5-2020	2	50,12415174	78,60893332	89,03767085	99,3872549	79,3	93,09	97,22	98,65	99,94	97,00	94,00	388
Nieuw "Eendenbek"	KN95 Mask	11-5-2020	2	47,51959355	78,15510002	89,22149	99,66666667	78,6	93,29	97,24	98,65	99,94	97,03	94,00	389
"PINTURA"	KN95 Mask	11-5-2020	2	74,44214863	91,98831445	95,88882455	99,68051118	90,5	93,31	97,24	98,69	99,95	97,06	94,00	390
"ANTHURA"	KN95 Mask	11-5-2020	2	72,09263201	90,1762021	95,13344691	99,48453608	89,2	93,57	97,26	98,70	99,95	97,06	94,00	391
"SINPUL"	KN95 Mask	11-5-2020	2	98,91832976	99,78026889	99,92023561	99,70845481	99,6	93,57	97,26	98,74	99,95	97,16	94,00	392
"KEES WEELE"	KN95 Mask	11-5-2020	2	52,73032104	74,49497758	82,56361923	99,14529915	77,2	93,66	97,36	98,80	99,95	97,22	94,00	393
Sichuan Lezhi Guijun Sanitary Material Co., LTD -KN95	KN95 Mask	11-5-2020	2	82,72832311	96,03270315	98,38485585	99,63235294	94,2	93,81	97,49	98,81	99,95	97,26	94,00	394
Shenzhen Leqi Intelligent Technology CO. LTD- KN95 M	KN95 Mask	11-5-2020	2	65,22739395	86,75311022	95,24308235	99,67948718	86,7	93,81	97,55	98,82	99,95	97,30	94,00	395
Jiangmen Dalfis Technology Co., Ltd. - Model KZ-001	KN95 Mask	11-5-2020	2	42,51161565	71,05801957	81,68963017	96,81372549	73,0	94,01	97,57	98,86	99,96	97,38	94,00	396
Huata HT9510	KN95 Mask	12-5-2020	2	97,59451489	98,81114869	99,21506063	99,70414201	98,8	94,20	97,66	98,86	99,96	97,40	94,00	397
Huata HT9510	KN95 Mask	12-5-2020	2	98,03570828	99,12155287	99,37637564	99,87405542	99,1	94,25	97,74	98,90	99,96	97,41	94,00	398
unknown	unknown	13-5-2020	3	43,00912821	55,69617782	66,15706259	98,57357357	65,9	94,31	97,78	98,93	99,96	97,42	94,00	399
unknown	GB262-2006KN95	13-5-2020	2	74,66826595	79,12414135	83,06226082	97,85894207	83,7	94,36	97,78	98,93	99,96	97,43	94,00	400
unknown	KN95 GB2626-2006	13-5-2020	2	58,45210599	78,55219258	88,49248736	99,89384289	81,3	94,37	97,85	98,94	99,96	97,45	94,00	401
unknown	GB2626-2006KN95	13-5-2020	2	28,09010901	44,52131344	60,57180366	97,82608696	57,8	94,53	97,86	98,95	99,97	97,53	94,00	402
unknown	1003 2626-2006KN95	13-5-2020	1	27,25809577	47,79913205	64,82461712	100	60,0	94,66	97,87	98,97	99,97	97,53	94,00	403
unknown	KN95 Mask	13-5-2020	1	80,66849748	92,49861836	96,77585614	99,64664311	92,4	94,67	97,91	98,98	99,97	97,53	94,00	404
unknown	KN95 Mask	13-5-2020	1	50,14545351	71,79966096	82,97851751	99,77011494	76,2	94,83	97,98	98,98	99,97	97,54	94,00	405
unknown	KN95 Mask	13-5-2020	1	87,44976581	92,4578482	94,90032901	99,77011494	93,6	94,85	97,99	98,98	99,97	97,54	94,00	406
unknown	KN95 Mask	13-5-2020	1	70,54419056	78,96482808	85,88235294	99,50331126	83,7	94,96	98,02	98,99	99,97	97,59	94,00	407
unknown	KN95 Mask	14-5-2020	2	59,74774358	77,22656304	86,6702901	99,33333333	80,7	95,01	98,06	99,02	99,97	97,60	94,00	408
unknown	KN95 Mask	14-5-2020	2	36,32465015	50,17994225	61,96983977	97,96460177	61,6	95,05	98,08	99,04	99,98	97,85	94,00	409
unknown	KN95 Mask	14-5-2020	2	37,72668834	52,08866185	63,55832943	96,80073126	62,5	95,07	98,11	99,05	99,98	97,96	94,00	410
unknown	KN95 Mask	14-5-2020	2	63,60595967	87,2355095	94,4662096	99,93946731	86,3	95,20	98,17	99,05	100,00	97,96	94,00	411
unknown	KN95 Mask	14-5-2020	2	62,02577878	78,61193432	88,00305057	99,84836998	82,1	95,44	98,30	99,06	100,00	97,98	94,00	412
unknown	KN95 Mask	14-5-2020	2	93,80778171	97,77582684	98,98072646	99,60087083	97,5	95,54	98,34	99,07	100,00	97,98	94,00	413
UFA	KN95	15-5-2020	2	27,054135	46,004148	60,85941444	91,66666667	56,4	95,57	98,39	99,12	100,00	97,99	94,00	414
UFA	KN95	15-5-2020	2	27,054135	46,004148	60,85941444	91,66666667	56,4	95,62	98,43	99,14	100,00	98,00	94,00	415
UFA (Sample 2)	KN95	15-5-2020	2	83,46818606	93,15574241	96,51813918	99,70817121	93,2	95,63	98,47	99,15	100,00	98,03	94,00	416
UFA (Sample 3)	KN95	15-5-2020	2	67,72922837	83,08266996	90,27005947	99,57627119	85,2	95,88	98,47	99,20	100,00	98,11	94,00	417
UFA (Sample 4)	KN95	15-5-2020	2	80,62229729	91,38473387	95,60034444	100	91,9	95,93	98,50	99,22	100,00	98,11	94,00	418
UFA (Sample 5)	KN95	15-5-2020	2	81,93929177	97,26098459	96,68640544	100	94,0	95,93	98,53	99,22	100,00	98,13	94,00	419
UFA (Sample 6)	KN95	15-5-2020	2	89,32676667	95,48520376	98,96892245	99,66887417	95,9	95,94	98,55	99,22	100,00	98,17	94,00	420
Face Mask Badge 1	KN95	15-5-2020	2	61,8061665	75,37454781	84,410764	99,47807933	80,3	95,95	98,56	99,22	100,00	98,19	94,00	421
Face Mask Badge 2	KN95	15-5-2020	2	49,5959528	64,92599906	76,98148449	99,71014493	72,8	96,02	98,57	99,22	100,00	98,21	94,00	422
Huizhou - GB2626-2006	KN95	15-5-2020	2	93,65785549	97,23685561	98,73587951	99,87341772	97,4	96,02	98,57	99,23	100,00	98,25	94,00	423
"Neusmondmasker"	KN95	15-5-2020	2	32,68282979	57,38295318	73,29631499	99,54648526	65,7	96,11	98,59	99,24	100,00	98,29	94,00	424
Shenzen Core Imago Co. Ltd	KN96	15-5-2020	2	75,16782541	86,57385986	92,91158537	100	88,7	96,12	98,61	99,25	100,00	98,32	94,00	425
Jiandi KN95 Respirator	KN95	15-5-2020	2	83,40016258	91,88454569	96,14404341	98,58695652	92,5	96,22	98,69	99,27	100,00	98,34	94,00	426
Tiadakang	KN95	15-5-2020	2	92,20150453	97,21614463	98,80317419	99,02723735	96,8	96,25	98,71	99,28	100,00	98,40	94,00	427
BioFil MY3D2 FFP2	KN95	15-5-2020	2	99,02760532	99,74797544	99,88356862	100	99,7	96,30	98,72	99,30	100,00	98,43	94,00	428
HT9510	(Sample 1)	18-5-2020	2	81,85587561	89,82605998	94,06270054	99,7002997	91,4	96,34	98,73	99,31	100,00	98,47	94,00	429
HT9510	(Sample 2)	18-5-2020	2	87,25265916	94,52443302	97,22796856	99,9382716	94,7	96,34	98,79	99,32	100,00	98,49	94,00	430
Masker 1	Unwashed	18-5-2020	2	16,87307883	35,07426263	49,4241362	91,94029851	48,3	96,35	98,80	99,38	100,00	98,50	94,00	431
Multi-Purpose protective Face mask	KN95	18-5-2020	2	99,28929893	99,76318256	99,84068627	98,83040936	99,4	96,42	98,81	99,38	100,00	98,53	94,00	432
Civil Haoyang Mask	Sample 1	18-5-2020	2	13,39116233	30,24495639	41,28771505	95,1754386	45,0	96,47	98,81	99,38	100,00	98,57	94,00	433
Civil Haoyang Mask	Sample 2	18-5-2020	2	9,94182299	21,40501732	33,10956365	90,51724138	38,7	96,54	98,81	99,39	100,00	98,58	94,00	434
Protective Maski BFE>95%	KN95	18-5-2020	2	53,26748135	72,11256811	82,00757576	99,81718464	76,8	96,56	98,83	99,41	100,00	98,59	94,00	435
GB2626-2006	KN95	18-5-2020	2	79,46297637	90,04220689	94,68134821	99,87113402	91,0	96,56	98,86	99,41	100,00	98,59	94,00	436
San Bang 9051A	FFP2	18-5-2020	2	91,23028197	95,99014132	98,17277979	99,90825688	96,3	96,79	98,86	99,42	100,00	98,60	94,00	437
JL-C3008	FFP2	18-5-2020	2	68,80428843	82,19703006	89,61482365	99,67741935	85,1	96,80	98,86	99,43	100,00	98,63	94,00	438
GIME 9122	FFP2	18-5-2020	2	97,32440245	98,29670169	98,64181276	98,15789474	98,1	96,92	98,93	99,44	100,00	98,64	94,00	439
Allcare Disposable surgical face mask	Civil	18-5-2020	2	17,81153955	37,75297004	52,72379101	97,23557692	51,4	96,98	98,94	99,44	100,00	98,65	94,00	440
unknown	unknown	14-5-2020	2	63,60595967	87,2355095	94,4662096	99,93946731	86,3	97,00	98,99	99,44	100,00	98,67	94,00	441
unknown	unknown	14-5-2020	2	62,02577878	78,61193432	88,00305057	99,84836998	82,1	97,03	99,00	99,44	100,00	98,68	94,00	442
unknown	unknown	14-5-2020	2	93,80778171	97,77582684	98,98072646	99,60087083	97,5	97,09	99,00	99,46	100,00	98,69	94,00	443
Mask # 24	KN95	22-5-2020	2	44,49838567	59,59960119	67,63864694	99,56843403	67,8	97,16	99,00	99,49	100,00	98,69	94,00	444
Mask # 25	KN95	22-5-2020	2	71,92441568	84,80530764	90,25952872	99,95704467	86,7	97,32	99,04	99,52	100,00	98,74	94,00	445
Mask # 26	KN95	22-5-2020	2	71,39358845	84,53995389	90,22847053	99,82517483	86,5	97,40	99,08	99,55	100,00	98,78	94,00	446
Mask # 27	Other	22-5-2020	2	99,53415773	99,82760744	99,88852637	100	99,8	97,43	99,12	99,55	100,00	98,80	94,00	447
Mask # 28	FFP2	22-5-2020	2	82,22045806	91,9004056	95,43758573	92,59615385	90,5	97,59	99,23	99,55	100,00	98,81	94,00	448
Mask # 29	Other	22-5-2020	2	80,13343591	93,38589634	96,79321995	99,94343891	92,6	97,60	99,23	99,59	100,00	98,83	94,00	449
Mask # 30	Other	22-5-2020	2	96,54075738	98,79339555	99,44352908	100	98,7	98,04	99,23	99,59	100,00	98,83	94,00	450
Mask #31	FFP2	22-5-2020	2	95,93563672	97,98549123	98,46385895	99,71617786	98,0	98,08	99,25	99,61	100,00	98,94	94,00	451
Mask # 32	FFP 2	22-5-2020	2	92,63979615	95,36749847	96,29785383	98,87218045	95,8	98,08	99,25	99,61	100,00	98,96	94,00	452
Mask # 34	FFP2	22-5-2020	2	87,45574303	94,07547361	96,54713482	99,93297587	94,5	98,10	99,29	99,62	100,00	98,96	94,00	453
Model 1 FFP3	KN95	22-5-2022	2	31,75018722	60,76867501	73,24051743	98,83990719	66,1	98,10	99,31	99,63	100,00	99,01	94,00	454
Model 2 FFP3 2020	KN95	22-5-2022	2	95,05337731	98,56167202	99,41742009	99,62941847	98,2	98,13	99,34	99,65	100,00	99,04	94,00	455
Model 3 CE FFP3	KN95	22-5-2022	2	99,55001912	99,81641752	99,93012167	99,96632997	99,8	98,26	99,35	99,65	100,00	99,04	94,00	456
Model 4 FFP3 (Enkel)	KN95	22-5-2022	3	95,57070697	98,81234266	99,55409643	99,9327957	98,5	98,30	99,37	99,68	100,00	99,08	94,00	457
Model 5 FFP3	KN95	22-5-2022	4	88,02227102	94,43175402	97,3750965	99,8046875	94,9	98,30	99,39	99,70	100,00	99,10	94,00	458
Model 6 FFP3	KN95	22-5-2022	5	66,43022983	90,27140793	96,43426295	99,88636364	88,3	98,51	99,39	99,75	100,00	99,16	94,00	459
Tomson Newt Protective Mask	KN95	22-5-2022	2	91,12525144	95,86542782	98,10088119	99,92343032	96,3	98,63	99,41	99,75	100,00	99,24	94,00	460
shell with Self Imbibition Filter	KN95	22-5-2022	2	88,63049846	94,04779664	97,05466374	99,62593516	94,8	98,68	99,45	99,76	100,00	99,31	94,00	461
shell Self Imbibition Filter	KN95	22-5-2022	2	57,32402316	72,79424347	84,20122888	100	78,6	98,68	99,47	99,77	100,00	99,31	94,00	462
FFP1	Mask	22-5-2022	2	3,677678942	9,655414796	18,67741254	87,22707424	29,8	98,80	99,58	99,79	100,00	99,33	94,00	463
IMD	KN95	22-5-2022	2	54,91835577	74,49143243	86,01062192	99,81167608	78,8	98,92	99,64	99,81	100,00	99,38	94,00	464
JMFT	KN95	22-5-2022	2	85,40586597	93,80016935	97,44655582	99,93498049	94,1	98,94	99,66	99,83	100,00	99,43	94,00	465
FPG	KN95	22-5-2022	2	25,30588193	50,53696371	67,89574062	92,72890485	59,1	98,97	99,69	99,84	100,00	99,44	94,00	466
Type A FFP2	KN95	22-5-2022	2	24,2998544	60,47747237	78,30503555	99,89506821	65,7	99,01	99,71	99,85	100,00	99,46	94,00	467
Type B FFP2	KN95	22-5-2022	2	90,89574333	96,37585214	98,40071948	100	96,4	99,03	99,73	99,87	100,00	99,46	94,00	468
Unknown	KN95	22-5-2022	2	87,65360386	96,03256875	98,53520708	100	95,6	99,29	99,75	99,88	100,00	99,49	94,00	469
N95 particular respirator	KN95	22-5-2022	2	71,34899115	86,97535239	93,64737441	99,67948718	87,9	99,36	99,76	99,89	100,00	99,58	94,00	470
Tongjian Neuclip IN	KN95	22-5-2022	2	88,15071423	96,36200798	98,61911847	99,32975871	95,6	99,52	99,78	99,89	100,00	99,62	94,00	471
Tongjian Neusclip OUT	KN95	22-5-2022	2	53,42670446	76,85181228	87,86432589	99,90421456	79,5	99,53	99,81	99,90	100,00	99,66	94,00	472
MASK Disposable Protective	KN95	22-5-2022	2	94,00710214	98,33743585	99,48575626	100	98,0	99,55	99,82	99,92	100,00	99,81	94,00	473

Medline	KN95	22-5-2022	2	68,76500622	84,93856978	91,97828709	99,55116697	86,3	99,86	99,83	99,93	100,00	99,82	94,00	474
Taidakang	N95 (GB2626-2006) Non-medical	22-5-2022	2	90,20049553	97,06062075	98,75425565	100	96,5							
												mean	83,2957		
												SD	16,06156		
