# Supplementary material for: Can sterilization of disposable face masks be an alternative for imported face masks? A nationwide field study including 19 sterilization departments and 471 imported brand types during COVID-19 shortages
Source: PLoS One. 2021 Sep 14;16(9):e0257468. doi: 10.1371/journal.pone.0257468 (PMC8439445; doi:10.1371/journal.pone.0257468)

Testing pressure drop over filter material **Supplemental file 8**

#	special case (brand)	output	pressure [Pa]	pressure drop [delta Pa]	reason (0.3/0.5/1/5 mu)
1	bolisi	0,917	-233,3333333	-233,3333333	scoorde 99/99/100/100
2	IVROU China shape	0,83	541,3333333	541,3333333	scoorde 99/99/100/100
3	IVROU 3M shape	0,74	457,3333333	457,3333333	scoorde 99/99/100/100
4	chinese KN95	4,217	3702,533333	3702,533333	scoorde 99/99/100/100
5	RYK 3D protecctice KN95	0,8	513,3333333	513,3333333	scoorde 99/99/100/100
6	DrCare+ moswl 9550	0,91	616	616	scoorde 99/99/100/100
7	Tomson Newt	0,705	424,6666667	424,6666667	scoorde 99/99/100/100
8	EAC	0,84	550,6666667	550,6666667	scoorde 100/100/100/100
9	Unknown	0,509	241,7333333	241,7333333	scoorde 99.6/99.8/100/100/
10	Ryzur (Wit)	0,732	449,8666667	449,8666667	scoorde 100/100/100/100
11	Air Queen	0,68	401,3333333	401,3333333	scoorde 98/100/100/100
12	Zhonglang	1,009	708,4	708,4	scoorde 97/100/100/100
13	Purvigor	1,169	857,7333333	857,7333333	scoorde 95/99/100/100
14	KN95 (Blue)	0,65	373,3333333	373,3333333	scoorde 99/100/100/100
15	airmax	0,761	476,9333333	476,9333333	scoorde 99/100/100/100
16	1206 GB2626-2006KN95	0,722	440,5333333	440,5333333	scoorde 99/100/100/100
17	Newt KN95 (Mask 1 from te	0,692	412,5333333	412,5333333	scoorde 95/99/100/100
18	Newt KN95 (Mask 2 from te	0,69	410,6666667	410,6666667	scoorde 99/100/100/100
19	Huata HT9510	1,045	742	742	scoorde 98/100/100/100
20	Huata HT9510	1,052	748,5333333	748,5333333	scoorde 99/100/100/100
21	Multi Purpose Face Mask	0,89	597,3333333	597,3333333	scoorde 99/100/100/100
22	GIME 9122	0,853	562,8	562,8	scoorde 95/99/100/100
23	Mask #27 (3m 8833)	0,85	560	560	scoorde 96/99/100/100
24	Model 3 CE FFP3	0,56	289,3333333	289,3333333	scoorde 99/100/100/100
25	Kinyoka	4,5	3966,67	3966,67	scoorde 99/100/100/100
26	white label	3,7	3220,00	3220,00	scoorde 98/100/100/100
27	white label	4,1	3593,33	3593,33	scoorde 99/100/100/100

mean all	0	pressure drop [delta Pa]	in bar	shape	good	middle	bad
1	0,7	251,0666667	0,260777344	cone	0,260777	0	0
2	0,7	261,3333333	0,271441102	cone	0,271441	0	0
3	0,7	298,6666667	0,233145507	wide beak model	0,233146	0	0
4	0,7	382,6666667	0,397467328	cone	0,397467	0	0
5	0,7	410,6666667	0,426550303	cone	0,42655	0	0
6	0,7	420	0,436244628	cone	0,436245	0	0
7	0,7	421,8666667	0,438183493	cone	0,438183	0	0
8	0,7	434	0,450786116	cone	0,450786	0	0
9	0,7	449,8666667	0,467266468	cone	0,467266	0	0
10	0,7	459,2	0,476960793	cone	0,476961	0	0
11	0,7	466,6666667	0,484716253	cone	0,484716	0	0
12	0,7	486,2666667	0,505074336	cone	0,505074	0	0
13	0,7	522,6666667	0,542882204	cone	0,542882	0	0
14	0,7	550,6666667	0,571965179	cone	0,571965	0	0
15	0,7	560	0,581659504	cone	0,58166	0	0
16	0,7	569,3333333	0,591353829	cone	0,591354	0	0
17	0,7	572,1333333	0,594262127	cone	0,594262	0	0
18	0,7	606,6666667	0,630131129	cone	0,630131	0	0
19	0,7	625,3333333	0,64951978	cone		0,64952	0
20	0,7	717,7333333	0,745493598	cone		0,745494	0
21	0,7	751,3333333	0,780393168	cone		0,780393	0
22	0,7	757,8666667	0,787179196	cone		0,787179	0
23	0,7	867,0666667	0,900602799	cone		0,900603	0
24	0,7	3220	3,344542149	cone			3,344542149
25	0,7	3593	3,731968926	cone			3,731968926
26	0,7	3711,866667	3,85543308	cone			3,85543308
27	0,7	3976	4,129782479	cone			4,129782479

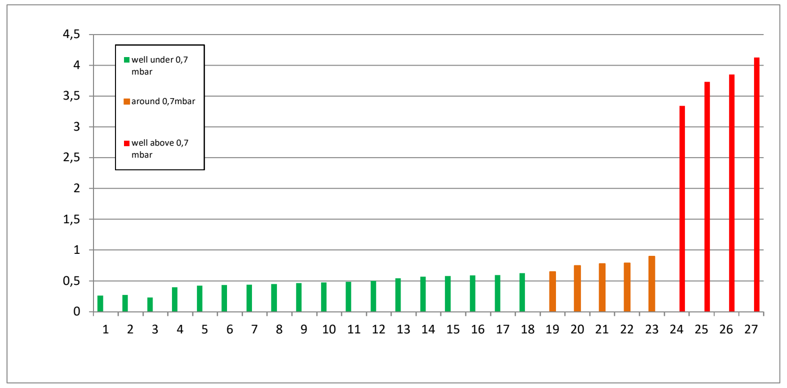

Supplement: S8 File — (PDF) [file pone.0257468.s008.pdf]
