# Supplementary material for: Can sterilization of disposable face masks be an alternative for imported face masks? A nationwide field study including 19 sterilization departments and 471 imported brand types during COVID-19 shortages
Source: PLoS One. 2021 Sep 14;16(9):e0257468. doi: 10.1371/journal.pone.0257468 (PMC8439445; doi:10.1371/journal.pone.0257468)
Supplement: Attachment — Submitted filename: Reviewer Comments_ID PONE-D-21-05058_R1.docx [file pone.0257468.s010.docx]

**Response to Reviewers’ Comments**

**Ref: Submission ID:** PONE-D-21-05058

“Sterilization of disposable face masks with respect to COVID-19 shortages; a nation-wide field study including 19 sterilization departments”.

We like to thank the reviewers for their valuable feedback. The responses to the comments follow the below remarks.

1. Is the manuscript technically sound, and do the data support the conclusions?

The manuscript must describe a technically sound piece of scientific research with data that supports the conclusions. Experiments must have been conducted rigorously, with appropriate controls, replication, and sample sizes. The conclusions must be drawn appropriately based on the data presented.

Reviewer #1: Partly

Reviewer #2: Yes

2. Has the statistical analysis been performed appropriately and rigorously?

Reviewer #1: N/A

Reviewer #2: Yes

3. Have the authors made all data underlying the findings in their manuscript fully available?

The [PLOS Data policy](https://urldefense.proofpoint.com/v2/url?u=http-3A__www.plosone.org_static_policies.action-23sharing&d=DwMGaQ&c=XYzUhXBD2cD-CornpT4QE19xOJBbRy-TBPLK0X9U2o8&r=XGDLKDP8wMEG6Uf-dzGknRQSssQkKhk6UCISNKPIEgM&m=CQCWrgTYYFqwlUUKWLfnce4IMG70o5M7D1r6Icu2tM0&s=CzlxdibLb3sVQvidVdz1Z55wC_wMRuHHHEhKHRj74H0&e=) requires authors to make all data underlying the findings described in their manuscript fully available without restriction, with rare exception (please refer to the Data Availability Statement in the manuscript PDF file). The data should be provided as part of the manuscript or its supporting information, or deposited to a public repository. For example, in addition to summary statistics, the data points behind means, medians and variance measures should be available. If there are restrictions on publicly sharing data—e.g. participant privacy or use of data from a third party—those must be specified.

Reviewer #1: Yes

Reviewer #2: Yes

4. Is the manuscript presented in an intelligible fashion and written in standard English?

PLOS ONE does not copyedit accepted manuscripts, so the language in submitted articles must be clear, correct, and unambiguous. Any typographical or grammatical errors should be corrected at revision, so please note any specific errors here.

Reviewer #1: Yes

Reviewer #2: Yes

5. Review Comments to the Author

**Comments and responses:**

**Comment 1: Academic Editor**

Based on reviewer 1 comment and my own evaluation I feel the experimental design, and the results obtained could not justify the conclusions. Further tests and characterizations were required to justify the major conclusions. Therefore I reject the submission however if the authors are willing to undertake additional tests and revisions as suggested by the reviewers, they can submit the revised version as a new submission at later point of time.

a) use of dry particles, b) mechanisms of sterilization for different masks not discussed, and (c) effects of sterilization on the electrostatic charge not considered. I tend to concur with the reviewer 1, that the authors oversimplified the experimental treatment, and in the process may have overstated the conclusions.

Amitava Mukherjee, ME, Ph.D.

**Response:**

We like to thank Professor Amitava Mukherjee for taking the time to discuss this decision with the authors over email. We did look critically at our conclusions and agree with the editor that restating is needed as there are more factors at play when implementing a new process. Therefore we rephrased the section about safe implementation of the 121 sterilization process to: *“The data from the six different installations show that the 121°C sterilization process can be safely implemented as long as proper testing of each batch is possible and the process and logistics are well controlled. The new PFE testing method proved to be accurate enough to determine degeneration of the mask material after sterilization and to determine the material quality of imported face masks. As FTIR, XRD and DSC indicate that all layers from both masks are made from PP, future testing is needed to determine if differences in PFE outcome after sterilization with 121 °C steam or H_2_O_2_ plasma sterilization can be explained by the level of crystallinity and the orientation and dimensions of the fibres and potential proprietary treatment in the layers of the face mask.”*

In addition we improved the conclusion in the abstract to be more aligned with the actual study as we investigated the mask material and not the complete mask as a hole.

We are happy that we were invited to explain our point of view and to provide answers to the questions of the reviewers in the following section.

**Reviewer #1**

**Comment 1:**

The manuscript titled “sterilization of disposable face masks with respect to COVID-19 shortage: a nationwide field study including 19 sterilisation departments” was submitted by Straten et al. The idea of the manuscript is simple to assess the quality of sterilized and imported face masks for the safety of use. It has some practical meaning under the current pandemic situation. However, the assessment that authors took was somewhat simplified such as only using the dry particles, not mentioned the mechanisms of sterilization for different masks, which could significantly affect the outcomes for different type of masks and make the assessment less meaningful.

**Response 1:**

The reviewer correctly mentioned that we took a simplified approach with dry particles as this kind of test setup can be easily build and is still accurate enough to identify absolute and relative changes in filtration capacity. This approach, as a methodology, was already used and published in 3 of our previous articles as well as validated against the standard continues flow system as described in our proposed work. Moreover, 19 hospitals including our Ministry of Health, Welfare and Sports used our approach to ensure proper functioning of over 80 million new face masks entering the Netherlands. All those parties were convinced that this approach was the best next thing that proofed to be reliable enough in times no other test facilities were available.

For all tested masks it is indicated in the supplemental excel lists how often sterilization was done, in what kind of hospital setting and what method was used. Maybe this was missed by reviewer 1. Therefore we now clarified the conditions better at the beginning of each supplemental file

Regarding the proposed test as well as the mechanisms of sterilization that we want to emphasize that we created a setup that is able to test the PFE and used it to answer 3 important research questions. However, within the study we presented we are not so much interested in the reason why deterioration of mask material happens and what type of effects play an important role of deterioration during sterilization. The important message the world need to see is: (1) that there are differences in PFE outcomes when different sterilization methods are used and (2) The PFE standard that is indicated on labels of hastily developed foreign masks is often wrong so when you think the mask protects you well it can happen that the mask is not filtering more than 15% of the relevant particle size instead of the 94% indicated on the label. In other extreme cases the breathability is so poor that air cannot pass the mask material at al.

We used the same mask to compare sterilization methods and we compared sterilized masks against new masks. Those are all comparison studies executed with one test setup with dry particles that give a very clear outcome regarding the PFE differences and therefore indicate objectively differences between sterilization methods and mask types despite the fact that dry particles may behave different than wet droplets as mentioned in the discussion (although our validation shows that this is not really the case). Therefore we think that the outcomes do have an important meaning within the context. Moreover, the difference in PFE outcome between new foreign masks and sterilized FFP2 masks most likely increases in favor of reprocessed masks when hospitals start to use the best sterilization method for their mask in any further crisis.

**Comment 2:**

It would be great if the author can discuss the effect of electrostatic charge on the PFE, which is thought to play a major role in the sterilization. It is obvious that either 121 sterilization or H2O2 plasma is difficult to recover the mask’s electrostatic charges. It would be further better if author can include this type of data by measuring the electrostatic charge related to the PFE.

**Response 2:**

Regarding the mechanisms of sterilization and effects of sterilization on the electrostatic charge, we can imagine that this is a very interesting research field of the specific reviewer. However, should the absence of those very interesting follow up studies that look into the pore size (those masks have no pores as they are made from spin bond stacked fibers) or complex measurements that indicate electrostatic fields inside masks be added to satisfy the reviewer? Those are very interesting but complex studies on itself and from our point of view those suggested studies do not add much as it does not help in answering the presented research questions or to improve our conclusion. However, as we do see the importance of the suggested studies we now added to the discussion: ”*As high quality FFP2 masks do react differently on different sterilisation methods, it is expected that the electrostatic charge of a mask has a major effect on the PFE especially for smaller lighter particles. Although not part of this explorative study, it is very interesting to investigate how either 121 ^0^C sterilization or H_2_O_2_ plasma affects the mask’s electrostatic charges and how this is related to the fiber orientation, pore size and openings between the stacked layers.”*

**Comment 3:**

Could author provide any data for the pore sizes for different masks used for the test? It would be great if it can be included in the material and method sections.

**Response 3:**

Although not part of this study it is interesting to relate pore size and electrostatic discharge to the PFE of a face mask, therefore we now added to the discussion: ” *Although not part of this explorative study, it is very interesting to investigate how either 121 ^0^C sterilization or H_2_O_2_ plasma affects the mask’s electrostatic charges and how this is related to the fiber orientation, pore size and openings between the stacked layers.”*

**Comment 4:**

Page 11, line 198, the authors mentioned that there were 5 brands in the Table 2, but there were only two brands with different treatments. More exactly say, you have two Table 2/II in the manuscript.

**Response 3:**

Unfortunately we misplaced table 1 and 2 after we removed a redundant table from an earlier version. We corrected and relabeled table I and table II and checked the references to the tables.

**Reviewer #2:**

The current manuscript discusses about the efficiency of different methods to reuse the masks during the COVID-19 pandemic. This article is the need of the hour. However, there are a few suggestions added below to improve the manuscript.

**Comment 1:**

Rewrite the outcome of the research mentioned at the end of the abstract. “……. can result……”

**Response 1:**

We corrected and rephrased this sentence to : “*Although the PFE of tested face masks from different sterilization departments remained efficient, using different types of sterilization equipment can result in different PFE outcomes*”.

**Comment 2:**

Line 92-94: Why UV-B sterilization was not used? Kindly mention the reason.

**Response 2:**

We added the following sentence in the Introduction: “*UV-B sterilization was not considered as this method is not yet commonly used and readily available with hospital sterilization departments*”.

**Comment 3:**

Line156: Mention the European Norm number

**Response 3:**

We changed this to: “*Test Setup validation to the European Norm EN 13274-7.*”.

**Comment 4:**

Table 1 caption has to be rewritten as the table is not self explanatory. (Percentage of ?)

**Response 4:**

Unfortunately the two tables were wrongly labeled after table 1 was removed in an earlier version. We now removed Table 1 in that section of the text and corrected the labelling and structure.

**Comment 5:**

Line 206: Mention the Norm NEN-149

**Response 5:**

We changed this to: “*Test Setup validation to the European Norm EN 13274-7*”.

6. PLOS authors have the option to publish the peer review history of their article ([what does this mean?](https://urldefense.proofpoint.com/v2/url?u=https-3A__journals.plos.org_plosone_s_editorial-2Dand-2Dpeer-2Dreview-2Dprocess-23loc-2Dpeer-2Dreview-2Dhistory&d=DwMGaQ&c=XYzUhXBD2cD-CornpT4QE19xOJBbRy-TBPLK0X9U2o8&r=XGDLKDP8wMEG6Uf-dzGknRQSssQkKhk6UCISNKPIEgM&m=CQCWrgTYYFqwlUUKWLfnce4IMG70o5M7D1r6Icu2tM0&s=SXJNAwKn_bMybhcVk44vYRCk7cATNo-VFgVP1KbKiAw&e=)). If published, this will include your full peer review and any attached files.

If you choose “no”, your identity will remain anonymous but your review may still be made public.

**Do you want your identity to be public for this peer review?** For information about this choice, including consent withdrawal, please see our [Privacy Policy](https://urldefense.proofpoint.com/v2/url?u=https-3A__www.plos.org_privacy-2Dpolicy&d=DwMGaQ&c=XYzUhXBD2cD-CornpT4QE19xOJBbRy-TBPLK0X9U2o8&r=XGDLKDP8wMEG6Uf-dzGknRQSssQkKhk6UCISNKPIEgM&m=CQCWrgTYYFqwlUUKWLfnce4IMG70o5M7D1r6Icu2tM0&s=b0zDE9cFV29WwZuQxICvEzb4Svo7kB4XhZJ5MNzyKUk&e=).

Reviewer #1: No

Reviewer #2: **Yes:** Dr Jastin Samuel
