# Supplementary material for: Can sterilization of disposable face masks be an alternative for imported face masks? A nationwide field study including 19 sterilization departments and 471 imported brand types during COVID-19 shortages
Source: PLoS One. 2021 Sep 14;16(9):e0257468. doi: 10.1371/journal.pone.0257468 (PMC8439445; doi:10.1371/journal.pone.0257468)
Supplement: Attachment — Submitted filename: PLOS.docx [file pone.0257468.s011.docx]

The manuscript titled “sterilization of disposable face masks with respect to COVID-19 shortage: a nationwide field study including 19 sterilization departments” may be accepted after major revision. The comments are as follows:

1. The language of the manuscript should be improved throughout.
2. The authors need to strengthen the scientific interpretations of the facts thoroughly.
3. Disinfection processes for reuse have not been described for all types of masks.
4. Studies not showing how to remove retention of the virus in the mask.
