# Supplementary material for: Can sterilization of disposable face masks be an alternative for imported face masks? A nationwide field study including 19 sterilization departments and 471 imported brand types during COVID-19 shortages
Source: PLoS One. 2021 Sep 14;16(9):e0257468. doi: 10.1371/journal.pone.0257468 (PMC8439445; doi:10.1371/journal.pone.0257468)
Supplement: Attachment — Submitted filename: Response to Reviewers_Rebuttal_ID PONE-D-21-05058_R3.docx [file pone.0257468.s012.docx]

**Response to Reviewers’ Comments**

**Ref: Submission ID:** PONE-D-21-05058

“Can sterilization of disposable face masks be an alternative for imported face masks? A nationwide field study including 19 sterilization departments and 471 imported brand types during COVID-19 shortages”.

We would like to thank all the reviewers for their valuable feedback and for recognizing the urgency and relevance of our study.

**Comments and responses:**

**Academic Editor:** After careful consideration, we feel that it has merit but does not fully meet PLOS ONE’s publication criteria as it currently stands.

**Response:** To improve the quality of our work in order to better meet the publication criteria we revised the manuscript according to the responses raised by the academic editor and reviewers point by point.

**Comment 1:**	Please ensure that your manuscript meets PLOS ONE's style requirements, including those for file naming.

**Response:**	We changed the manuscript to meet the style requirements including the Title page which was adjusted with title in sentence case, authors with numbers in superscript. The physical address of the corresponding author was removed and initials included. The titles and subtitles in the manuscript were changed to Level 1, 2 and 3 headings with bold type fonts in sizes 18, 16 and 14. We renamed the Supplemental files according to the PLOS-ONE standards.

We also improved the style and formatted the content of all S-series Supplemental files for easier interpretation.

This round of comments by the reviewers made us realize that the title could be improved and changed this to:

“Can sterilization of disposable face masks be an alternative for imported face masks? A nationwide field study including 19 sterilization departments and 471 imported brand types during COVID-19 shortages”.

We believe this title is now a better cover for this research.

**Comment 2:**	In your Methods section, please clarify how imported masks were selected to be included in the analysis; and how many samples were tested for each brand.

**Response:**	Thank you for this valuable comment**.** To clarify this further we now added to the main text in the methods section in Lines 197 – 207:

“*To determine how many samples of imported face masks were needed, the variance of the PFE and pressure drop was determined on three imported face masks. Ten measurements conducted on each face mask type (S2 Supplemental file 2) showed that the largest variance was found in the 0.3 μm particle size category of 0.6%, 1.1% and 0.3% of the mean values respectively. The pressure drop measurements showed a variance of 0.7%, 1.8% and 1.8% of the mean values respectively. This low variance indicated consistent behavior of the filter materials. Combined with the importance of a short processing time it was determined to measure a minimum of two masks of each type that was provided by the clients. The averaged values are listed in S3 Supplemental file 3. In case a deviation of more than 10 % was found in the 0.3 μm category, two additional masks were tested and the supplier was notified. This data was excluded from the study.*”

We now added to the Results section in lines 280 - 283:

“*In two occasions the PFE data of the two tested masks of the same type deviated more than 10% (i.e. PFE of 67% vs 84% at 0.3 μm). Closer inspection revealed that the two masks had a different appearance as one had an additional logo in the shape of a heart”*

In the discussion we improved the section in line 362:

“*During the study period, it was observed two times that within a single batch of imported face masks, the quality and layout of the masks were different, despite being wrapped in the same packaging with the same printed PFE standard. This suggests that multiple factories were supplying to one brand.”*

**Reviewer #1:** The present manuscript discusses about the efficiency of different methods to reuse the masks, which is the critical need of the hour and can save both, the time and money during this pandemic time. The manuscript, in my opinion is acceptable for publication.

**Response:** We thank the reviewer for emphasizing the efficiency of our methods and the support for publication of this study.

**Reviewer #2:** The test conducted in this paper tests the used face masks for particle size range 0.3 to 5 micron. The low efficiency of the processed face masks at 0.3 microns is concerning and contradicts the conclusion.

**Response:**	We fully agree that it is concerning that the PFE efficiency after processing at 0.3 micron becomes very low in some cases. As this is one of the most important messages of this article, we now better aligned the conclusion by adding to the first sentence in line 390:

”*Sterilization of disposable face masks by means of standardized steam sterilization at 121°C could be an alternative against face mask shortages due to COVID-19 if the fit does not change and the filter materials are not significantly affected by the heat.”*

**Comment:** Furthermore, the difference in efficiency after reprocessing among the different brands concludes that only masks of a particular brand could be reused.

**Response:** As we fully agree that we can be more specific. We now added to the conclusions in lines 392 - 394:

“*The varying efficiency after reprocessing amongst different brands shows that only quality masks of particular brands such as 3M Aura 1862, 3M Aura 1873 and My-T-Gear 301 are suitable for limited reuse*”.

**Comment:** I am also concerned about the sample size and whether it accurately represents the entire population. There is no sample size test conducted to identify the required sample size to detect the variance to be checked.

**Response:**

This is a very good comment. Unfortunately the situation and nature of this study did not allow us to foresee from what parts of the world, face masks were coming from and how many would reach our emergency test location. What we did, but not previously described in our work, is that we tested the variance of 3 different foreign, imported masks types by measuring 10 samples of each. This indicated a very low variance allowing us to set the sample size for each incoming batch of masks on two. In our response on comment 2 of the editor you can see the changes we made to the methods section. Furthermore, it proved to be true that in some cases manufacturers filled their boxes with masks coming from different manufacturers. This occurred twice in our study. Therefore, we improved this section in the discussion in lines 359-364, to be more specific:

“*During the study period, it was observed two times that within a single batch of imported face masks, the quality and layout of the masks were different, despite being wrapped in the same packaging with the same printed PFE standard. This suggests that multiple factories were supplying to one brand. In other cases, some masks (Figure 6) showed almost complete lack of air penetration due to the use of wrong materials or manufacturing processes.*”

**Reviewer #3:** The manuscript titled “sterilization of disposable face masks with respect to COVID-19 shortage: a nationwide field study including 19 sterilization departments” may be accepted after major revision. The comments are as follows:

**Comment 1:** The language of the manuscript should be improved throughout.

**Response:** The manuscript was revised by a native speaker and checked on language errors.

**Comment 2:** The authors need to strengthen the scientific interpretations of the facts thoroughly.

**Response:** We agree and now strengthened the scientific approach by explaining the methods that were used for sampling of the masks more concrete. This also allowed us to be more specific in the discussion about the obtained data from the results. Also we were able to improve the main conclusion by better explaining the limitations and boundary conditions when choosing for reprocessing masks. (see our responses above with comments to the editor and reviewer #2 for more details about the specific modifications made to the methods, results and conclusions)

**Comment 3:**. Disinfection processes for reuse have not been described for all types of masks.

**Response:** As we understand that it can be confusing that we used the sterilization equipment to decontaminate the masks in our new approach we now explicitly explain this in the first sentence of the section under Reprocessing by 121°C steam at CSA Services Sterilization in line 128 with:

“*Within this new reprocessing approach, decontamination was done solely by steam sterilization”*

**Comment 4:** Studies not showing how to remove retention of the virus in the mask.

**Response:**	In our earlier work we investigated if steam sterilization at 121°C is able to inactivate the coronavirus [9,14,15]. We agree with the reviewer that the retention is not described nor investigated as we were focusing on the inactivation during reprocessing only. As this is an important topic worth investigating, we included in the limitations the following sentence in lines 380-382:

*“**Although the study used a validated reprocessing method based on 121°C sterilization to inactivate the virus in the mask, the retention of the inactivated virus has not been studied and should be investigated further in future studies”.*
